# Modeling the influence of bacteria concentration on the mechanical properties of self-healing concrete (SHC) for sustainable bio-concrete structures

**DOI:** 10.1038/s41598-024-58666-8

**Published:** 2024-04-10

**Authors:** Kennedy C. Onyelowe, Ali F. H. Adam, Nestor Ulloa, Cesar Garcia, Alexis Ivan Andrade Valle, María Gabriela Zúñiga Rodríguez, Andrea Natali Zarate Villacres, Jamshid Shakeri, Lewechi Anyaogu, Mohammadreza Alimoradijazi, Nakkeeran Ganasen

**Affiliations:** 1 Department of Civil Engineering, Michael Okpara University of Agriculture, Umudike, Nigeria; 2 Department of Civil Engineering, University of the Peloponnese, 26334 Patras, Greece; 3 Department of Civil Engineering, College of Engineering Technologies, Al-Qubbah, Libya; 4 Facultad de Mecánica, Escuela Superior Politécnica de Chimborazo (ESPOCH), Panamericana Sur km. 1½, 060155 Riobamba, Ecuador; 5 Facultad de Ingeniería, Arquitectura, Universidad Nacional de Chimborazo (UNACH), 060501 Riobamba, Ecuador; 6 Facultad de Ingeniería, Ingeniería Civil, Universidad Nacional de Chimborazo (UNACH), 060501 Riobamba, Ecuador; 7 Heritage and the City, Universitat Politècnica de València, 46022 España, Valencia Spain; 8 Department of Mining Engineering, Faculty of Engineering, Hamedan University of Technology, Hamadan, Iran; 9 Department of Civil Engineering, School of Engineering and Engineering Technology, Federal University of Technology, Owerri, Nigeria; 10 Department of Computer Engineering, Faculty of Engineering, K. N. Toosi University of Technology, Tehran, Iran; 11 Department of Civil Engineering, SRM Institute of Science and Technology, Chennai, India; 12 Grupo de Investigación y Desarrollo de Nanotecnología, Materiales y Manufactura (GIDENM), Escuela Superior Politécnica de Chimborazo, Panamericana Sur Km. 1½, 060155 Riobamba, Ecuador

**Keywords:** Metaheuristic machine learning (MML), Response surface methodology (RSM), GWO, MVO, MFO, PSO, and WOA, Bio-concrete, Bacteria concentration, Self-healing concrete (SHC), Engineering, Materials science

## Abstract

In this research paper, the intelligent learning abilities of the gray wolf optimization (GWO), multi-verse optimization (MVO), moth fly optimization, particle swarm optimization (PSO), and whale optimization algorithm (WOA) metaheuristic techniques and the response surface methodology (RSM) has been studied in the prediction of the mechanical properties of self-healing concrete. Bio-concrete technology stimulated by the concentration of bacteria has been utilized as a sustainable structural concrete for the future of the built environment. This is due to the recovery tendency of the concrete structures after noticeable structural failures. However, it requires a somewhat expensive exercise and technology to create the medium for the growth of the bacteria needed for this self-healing ability. The method of data gathering, analysis and intelligent prediction has been adopted to propose parametric relationships between the bacteria usage and the concrete performance in terms of strength and durability. This makes is cheaper to design self-healing concrete structures based on the optimized mathematical relationships and models proposed from this exercise. The performance of the models was tested by using the coefficient of determination (R^2^), root mean squared errors, mean absolute errors, mean squared errors, variance accounted for and the coefficient of error. At the end of the prediction protocol and model performance evaluation, it was found that the classified metaheuristic techniques outclassed the RSM due their ability to mimic human and animal genetics of mutation. Furthermore, it can be finally remarked that the GWO outclassed the other methods in predicting the concrete slump (Sl) with R^2^ of 0.998 and 0.989 for the train and test, respectively, the PSO outclassed the rest in predicting the flexural strength with R^2^ of 0.989 and 0.937 for train and test, respectively and the MVO outclassed the others in predicting the compressive strength with R^2^ of 0.998 and 0.958 for train and test, respectively.

## Introduction

**Figure 1:**
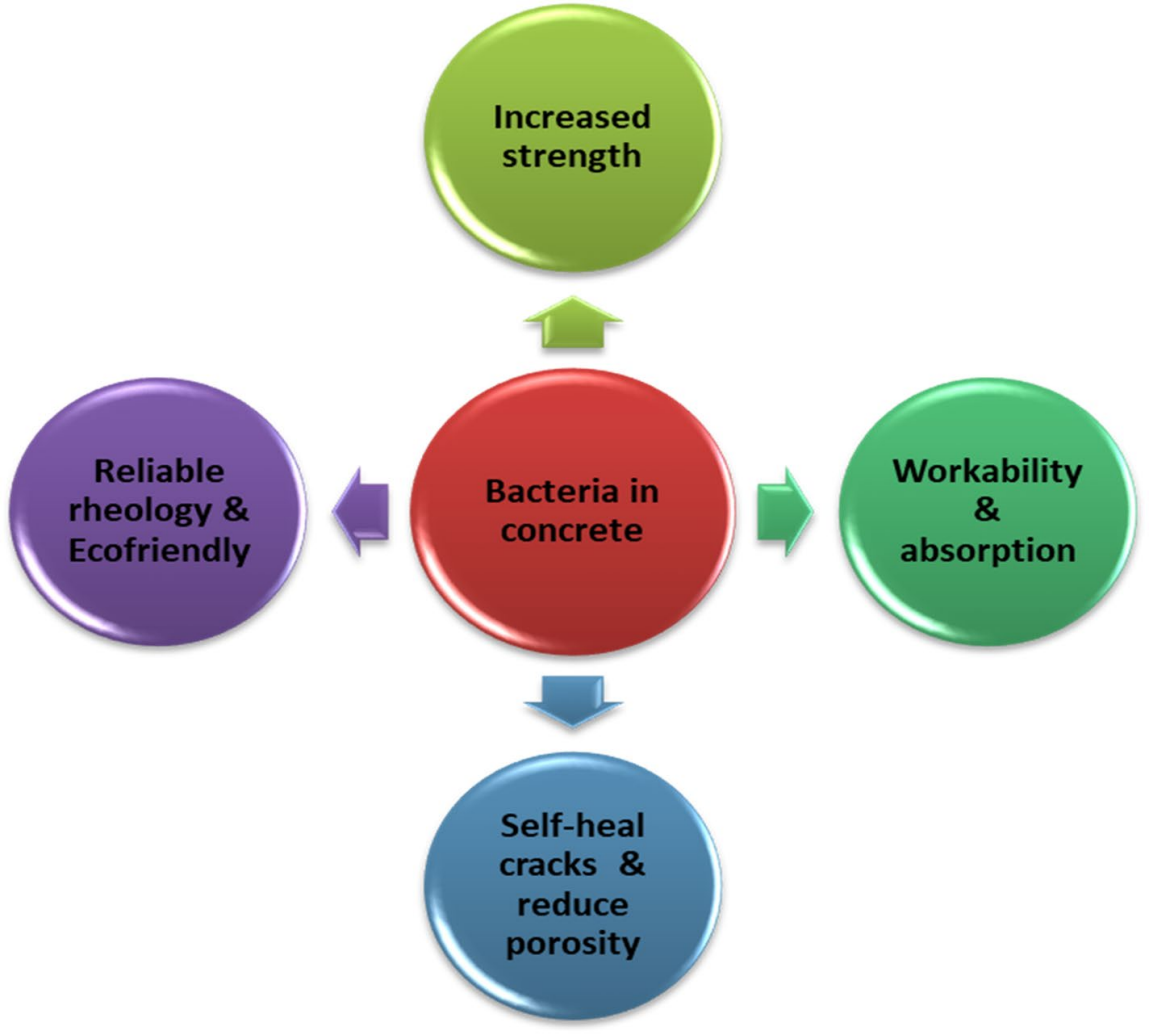
Structural benefits of bacillus subtilis in concrete.

Self-healing concrete is a type of concrete that has the ability to repair cracks and damage on its own^1^, (see Fig. 1). One of the key components of self-healing concrete is the incorporation of bacteria, typically of the species Bacillus or Sporosarcina, along with a calcium-based healing agent, such as calcium lactate or calcium carbonate^2^. The bacteria in self-healing concrete remain dormant until cracks occur in the concrete^3,4^. When water or moisture enters the cracks, it reactivates the bacteria^5^. The bacteria then consume the calcium lactate or calcium carbonate and produce limestone (calcium carbonate) as a byproduct^6^. This limestone fills the cracks, effectively healing the concrete^7^. The concentration of bacteria in self-healing concrete can have an influence on its strength and healing efficiency^8^. Here are some points to consider: Healing capacity: Higher concentrations of bacteria generally result in greater healing capacity^9^. This means that a higher number of bacteria can produce more limestone and effectively fill a larger number of cracks, improving the self-healing capability of the concrete^10^. Crack width: The concentration of bacteria can also impact the ability to heal wider cracks. If the concentration is too low, the bacteria may not be able to produce enough limestone to effectively close wider cracks^11^. In such cases, additional measures may be required to enhance the healing process, such as incorporating fibers or other materials to bridge wider cracks^12^. Strength considerations: While higher concentrations of bacteria can improve the self-healing capacity^13^, they may also have an impact on the strength of the concrete^14^. Excessive bacterial concentrations can potentially interfere with the cement hydration process or affect the overall structural integrity of the concrete^15^. Therefore, it is important to find the right balance between healing efficiency and maintaining adequate concrete strength^16^. Optimization: The optimal concentration of bacteria in self-healing concrete depends on various factors such as the specific bacteria used, the type of healing agent, the crack width expected, and the desired strength requirements^3,17^. Extensive research and testing are necessary to determine the most suitable concentration for a particular application^18^. It’s worth noting that the concentration of bacteria is just one factor in the overall design and performance of self-healing concrete^1,19^. Other factors, such as the selection of healing agents, the mix design, and the curing conditions, also play crucial roles in achieving the desired self-healing properties while maintaining the required strength and durability of the concrete structure^20^. Overall, the concentration of bacteria in self-healing concrete can influence its healing capacity, ability to close cracks of different widths, and potentially impact its strength^21^. The strength ranges can be either low (below 20 MPa), medium (20–40 MPa), high (40–80 MPa), or ultra-high (80–120 MPa and above)^22^. Careful consideration and optimization of bacterial concentrations are necessary to ensure effective self-healing while maintaining the structural integrity of the concrete^23^.

Medium-strength self-healing concrete is a type of concrete with a strength range of 20–40 MPa, that has the ability to repair cracks and damage autonomously without the need for human intervention^7,24^. It incorporates various healing mechanisms to restore its structural integrity and durability^25^. Here are some common techniques used in medium-strength self-healing concrete: Microencapsulated Healing Agents: This method involves incorporating tiny capsules filled with healing agents such as polymers, epoxy resins, or mineral-based materials into the concrete mixture^26^. When cracks form, these capsules rupture, releasing the healing agents into the cracks^27^. The agents then react with the surrounding environment to form a solid material that seals the crack^28^. Vascular Systems: Inspired by the human circulatory system, vascular systems in concrete involve embedding a network of hollow tubes or channels within the concrete^6,29^. These channels are filled with healing agents, such as chemical grouts or mineral solutions^30^. When cracks occur, the channels rupture, releasing the healing agents into the cracks^31^. The agents then harden and fill the voids, restoring the concrete’s integrity^11,32^. Autogenous Healing: Autogenous healing is a natural self-healing process of concrete^33^. It occurs when the unhydrated cement particles present in the concrete mix react with water in the presence of moisture, such as rainwater or humidity^4,19,34^. This reaction results in the formation of calcium carbonate, which fills the cracks and improves the concrete’s strength. Shape Memory Polymers: Shape memory polymers have the ability to return to their original shape after being deformed^18^. When used in self-healing concrete, embedded shape memory polymer fibers or particles can be activated by external stimuli, such as temperature or moisture changes^2^. Once activated, these polymers expand, closing the cracks and restoring the concrete’s integrity^12^. Medium-strength self-healing concrete offers several advantages, including enhanced durability, extended service life, and reduced maintenance costs^6^. It can be used in various applications, such as building construction, infrastructure projects, and transportation systems, where crack formation and damage are common^33^. However, it’s important to note that self-healing concrete is still a developing technology, and its widespread adoption and commercial availability may vary^34^. The concentration of bacteria in self-healing concrete can have an influence on its rheology and workability^35^. Rheology refers to the flow and deformation behavior of concrete, while workability refers to its ease of handling and placing during construction^34^. Here’s how the bacteria concentration can affect these properties: Viscosity: The bacteria and their metabolic byproducts can affect the rheology of the self-healing concrete^31–35^. Higher concentrations of bacteria can lead to increased viscosity, making the concrete more resistant to flow^35^. This increased viscosity can affect the workability of the concrete, making it more difficult to handle and place^23^. Water demand: Self-healing concrete typically requires a specific water-to-cement ratio for proper hydration and bacterial activity^25^. Higher bacterial concentrations may increase the water demand of the concrete mix, as bacteria require water for their metabolic processes^36^. This increased water demand can impact the workability of the concrete by requiring additional water to maintain the desired consistency^23–26^. Set time: Bacteria in self-healing concrete can influence the concrete’s setting time, which is the time it takes for the concrete to harden and gain strength^31^. Higher concentrations of bacteria can potentially accelerate or delay the setting time, depending on the specific bacterial species used and the conditions of the concrete mix^15^. Changes in setting time can affect the workability of the concrete by altering the available time for handling and placing^16^. Segregation and bleeding: Excessive bacterial concentrations can cause segregation and bleeding in the concrete mix^12^. Segregation refers to the separation of coarse aggregates from the mortar, while bleeding refers to the upward movement of water to the surface of freshly placed concrete^9^. Both segregation and bleeding can negatively impact the workability and homogeneity of the concrete, leading to potential structural issues^34^. To mitigate the potential negative effects of higher bacterial concentrations on rheology and workability, it is important to carefully optimize the mix design and construction practices^36^. The use of appropriate admixtures, such as superplasticizers, can help improve the flow and workability of self-healing concrete while maintaining the desired bacterial concentration^37^. It’s worth noting that the influence of bacteria concentration on rheology and workability is just one aspect to consider in the design and performance of self-healing concrete^1^. Other factors, such as the selection of bacteria, healing agents, aggregate gradation, and overall mix design, also play important roles^3^. Proper testing and evaluation are necessary to determine the optimal bacterial concentration that balances self-healing capabilities with acceptable rheology and workability characteristics^15–18^. Bacteria used in self-healing concrete are typically of the genus Bacillus or Sporosarcina^36^. These bacteria have the ability to produce a specific enzyme called urease, which plays a key role in the self-healing process^28^. Incorporation: The bacteria are introduced into the concrete during the mixing process^21^. They are usually in the form of spores, which remain dormant until conditions are favorable for growth^37^. Activation: When cracks form in the concrete, water seeps in and comes into contact with the bacterial spores. This triggers the activation of the bacteria^4–9^. Urease production: Once activated, the bacteria start to metabolize nutrients present in the concrete^38^. As a byproduct of their metabolism, they produce the enzyme urease^7,38^. Urease reaction: Urease catalyzes the hydrolysis of urea, which is commonly added to the concrete as a nutrient source for the bacteria^6^. This hydrolysis reaction produces calcium carbonate and ammonia^11^. Calcium carbonate formation: The calcium ions present in the concrete react with the produced carbonate ions to form calcium carbonate crystals^35–38^. These crystals fill the cracks, effectively sealing them. Self-healing: As the calcium carbonate crystals form and grow, they gradually fill the cracks and restore the integrity of the concrete^7–9,29^. This process can occur over a period of several weeks, depending on the extent of the damage^1^. It’s important to note that self-healing concrete utilizing bacteria is still an emerging technology and not yet widely implemented in construction projects^14,27^. Extensive research is ongoing to optimize the performance, durability, and practicality of this approach^31,39^. In self-healing concrete, the concentration of bacteria and the activation reaction are important factors that influence the effectiveness of the self-healing process^31^. Bacteria Concentration: The concentration of bacteria in self-healing concrete can vary depending on the specific application and desired outcome^26^. Generally, a higher concentration of bacteria leads to a more efficient healing process^38^. However, it’s important to strike a balance because an excessively high concentration may lead to competition for nutrients and limited space for bacterial growth^39^. The typical range of bacteria concentration in self-healing concrete is between 10^6 and 10^8 colony-forming units (CFU) per gram of concrete^40^. This concentration is achieved by adding specific amounts of bacteria-containing solution or powder during the concrete mixing process^7,15^. Activation Reaction: The activation of bacteria in self-healing concrete occurs when water enters the cracks and comes into contact with the dormant bacterial spores^19^. The presence of water triggers the germination of spores and the subsequent growth of bacteria^2–4^. Once activated, the bacteria start to consume nutrients present in the concrete, metabolize them, and produce the enzyme urease^6–11^. This urease production is an essential part of the self-healing process. The reaction can be summarized as follows:

1 $$ {\text{Bacteria }} + {\text{ Water }} + {\text{ Nutrients }} \to {\text{ Growth }} + {\text{ Urease}}\;{\text{Production}} $$

The bacteria use the nutrients, such as urea, as a food source for their growth and metabolism^8,40^. As a byproduct of their metabolism, they release urease enzyme, which initiates the hydrolysis of urea^6,9^. It’s worth noting that the specific activation reaction can vary depending on the type of bacteria used and the formulation of the self-healing concrete^10,20–24^. Different bacterial strains may have different nutrient requirements or metabolic pathways, but the general principle of bacterial growth and urease production remains consistent^7^. Optimizing both the bacteria concentration and the activation reaction is crucial for achieving effective self-healing properties in concrete^8^. Ongoing research aims to refine these parameters and develop standardized guidelines for incorporating bacteria into self-healing concrete^41–43^. The use of bacteria in self-healing concrete has the potential to enhance the sustainability of concrete structures in several ways: Extended Lifespan: Self-healing concrete reduces the need for frequent repairs and maintenance, thus extending the lifespan of concrete structures^3,42^. This leads to reduced resource consumption and waste generation associated with the construction and repair processes^44^. Reduced Material Consumption: By autonomously repairing cracks, self-healing concrete minimizes the need for additional materials, such as repair mortars or epoxy resins^45^. This can contribute to resource conservation and reduce the carbon footprint associated with the production and transportation of these materials^3^. Energy Savings: The self-healing process eliminates or reduces the need for manual intervention and repair work, which can be energy-intensive^35,42^. It eliminates the energy required for repair activities, such as drilling, patching, or replacing damaged concrete elements^43^. Improved Durability: Self-healing concrete can enhance the durability and resilience of structures^44^. By sealing cracks promptly, it prevents the ingress of water, chemicals, and other harmful substances that can lead to further deterioration^45^. This reduces the likelihood of structural failures and the need for major repairs or replacements^46^. Reduced Environmental Impact: The self-healing process of bacteria in concrete relies on the use of natural microorganisms^47^. Compared to traditional repair methods that may involve the use of synthetic materials or chemicals, self-healing concrete with bacteria has the potential for a lower environmental impact^48,49^. However, it’s important to consider some potential sustainability challenges associated with bacteria use in self-healing concrete: Energy and Resource Requirements: The production and cultivation of bacteria and the incorporation of bacteria into concrete require energy and resources^42,49^. The sustainability benefits of self-healing concrete need to be balanced against the environmental impacts associated with bacterial cultivation and incorporation processes^6,35^. Bacterial Viability: Ensuring the long-term viability and performance of bacteria in concrete structures is a challenge^49^. Factors such as harsh environmental conditions, nutrient availability, and competition with other microorganisms can affect the survival and effectiveness of bacteria over time^48^. Ongoing research is focused on optimizing bacterial strains and formulations to enhance their viability and longevity^49^. Regulatory Considerations: The use of bacteria in construction materials may involve regulatory considerations related to safety, health, and environmental impacts^2,17^. It’s important to conduct thorough assessments to ensure that the use of bacteria in self-healing concrete aligns with applicable regulations and standards^43–45^. Overall, while bacteria-based self-healing concrete offers potential sustainability benefits, further research and development are needed to optimize its performance, evaluate its life cycle impacts, and address any associated challenges^48^. Hence, this research work is focused on applying the metaheuristic machine learning and the symbolic response surface methodology methods in the prediction of the strengths of the bacterial-based self-healing concrete for use in the design and production of optimized materials-based bio-concrete at optimal rate of bacteria concentration. The research on "Modeling the influence of bacteria concentration on the mechanical properties of self-healing concrete (SHC) for sustainable bio-concrete structures" holds significant importance in several ways: Advancing Sustainable Construction: Self-healing concrete (SHC) offers a promising solution to extend the service life of concrete structures, thereby reducing the need for frequent repairs and replacements. By incorporating bacteria into concrete mixes to facilitate self-healing, the research contributes to the development of sustainable construction practices that minimize resource consumption and environmental impact. Enhancing Structural Integrity: Understanding the influence of bacteria concentration on the mechanical properties of SHC is essential for ensuring the structural integrity and performance of bio-concrete structures. By modeling these relationships, the research can provide valuable insights into optimizing the design and production of SHC to achieve desired mechanical properties and durability. Promoting Innovation in Concrete Technology: The incorporation of bacteria into concrete mixes represents an innovative approach to address common issues such as cracks and deterioration in concrete structures. By studying the effects of bacteria concentration on mechanical properties, the research contributes to the advancement of concrete technology and encourages the adoption of novel materials and methods in construction. Mitigating Maintenance Costs: Self-healing concrete has the potential to significantly reduce maintenance costs associated with concrete structures by autonomously repairing cracks and damage over time. By quantifying the relationship between bacteria concentration and mechanical properties, the research can help optimize SHC formulations to maximize healing efficiency and minimize maintenance requirements, leading to cost savings for infrastructure owners and operators. Improving Long-Term Durability: The durability of concrete structures is crucial for ensuring their long-term performance and resilience against environmental factors such as moisture, chemical exposure, and freeze–thaw cycles. By investigating how bacteria concentration influences mechanical properties, the research contributes to improving the long-term durability of bio-concrete structures, thereby extending their service life and reducing life-cycle costs. Addressing Infrastructure Challenges: Cracking and deterioration are common challenges faced by concrete infrastructure worldwide, leading to safety concerns, service disruptions, and costly repairs. By developing self-healing concrete technologies, the research addresses these challenges proactively, offering a sustainable and cost-effective solution to enhance the resilience and longevity of infrastructure systems. In summary, the research on modeling the influence of bacteria concentration on the mechanical properties of self-healing concrete for sustainable bio-concrete structures has significant implications for advancing sustainable construction practices, improving structural integrity, promoting innovation in concrete technology, mitigating maintenance costs, enhancing long-term durability, and addressing critical infrastructure challenges. More important to consider is the deployment of machine learning to forecast the behavior of the SHC for sustainable design of its properties.

## Methodology

### Data collection and preliminary analysis

**Figure 2:**
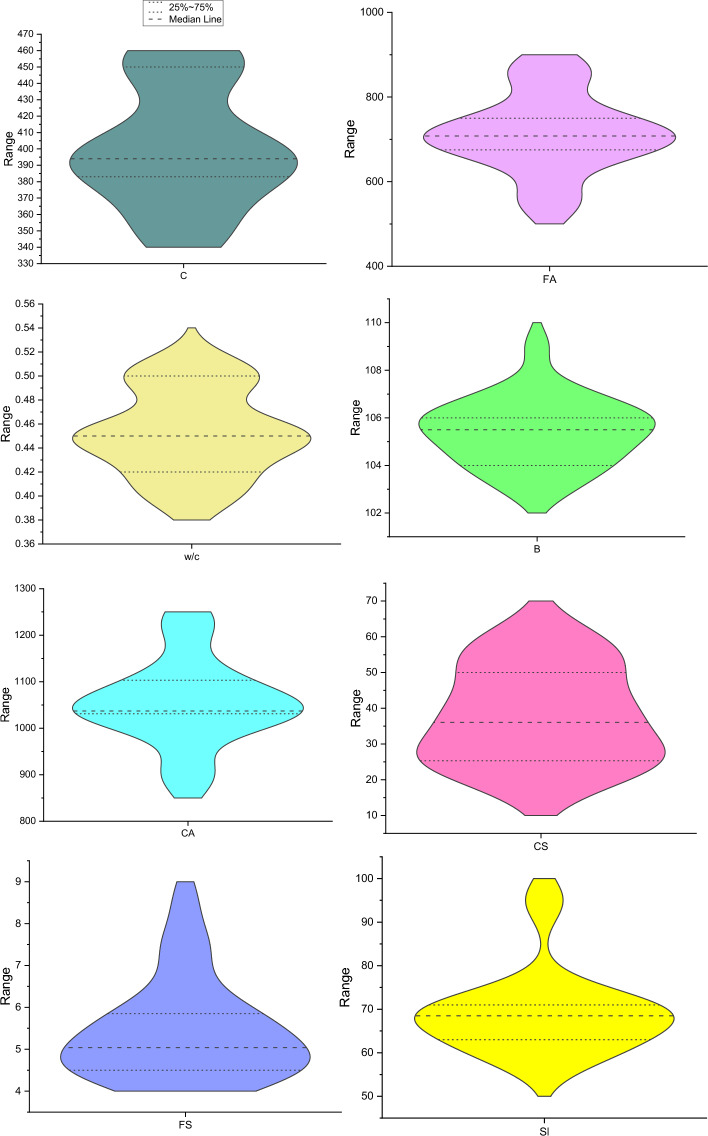
Violin plot of outputs and effective parameters.

**Figure 3:**
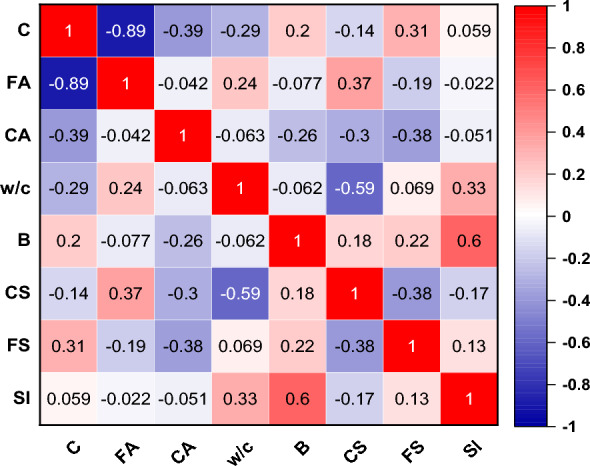
The correlation matrix of inputs.

The database has been collected from a previous research paper^49^, prepared, shuffled and used in the various models presented in this research paper. Table 1 presents the descriptive statistics of the outputs (compressive strength, slump and flexural strength) and the influential factors. This shows the minimum, maximum, mean, standard deviation, and variance of the collected entries of the SHC. Moreover, for the purpose of visually illustrating the statistical representation of the data, Fig. 2 exhibits a violin plot. This particular graphical depiction comprises both a boxplot and a density plot. The upper and lower boundary lines effectively demonstrate the span between the lower quartile (Q1) and the upper quartile (Q3). Meanwhile, the central line corresponds to the 95% confidence interval. The relationship between the independent variables known as the regressors (C, FA, w/c, B, and CA) and the studied mechanical properties of the bacterial-inspired self-healing concrete is also illustrated through the vibratory nodes of the violin. The width of the violin plots at any given point, which represents the probability density of the data at that value is more robust with FA, w/c, B, and CA showing the contributory strength they possessed in the studied mix. This shows that wider sections indicate higher density, while narrower sections such as in C, indicate lower density. It gives a visual representation of the distribution of the data. Figure 3 shows the correlation matrix between the input and the output parameters. It also presents the internal consistency between the inputs and the outputs. This further shows that it was w/c and B that showed good effects to only CS and SI, with degrees of 0.59 and 0.6, respectively. This further strengthened the need for machine learning predictions to achieve optimized mechanical properties of the bacterial-inspired self-healing concrete, regardless of the lack of internal consistency between the regressors and the targets.

**Table 1:** Descriptive statistics of outputs and influential factors.

Parameters	Unit	Sign	Min	Max	Mean	SD	Variance
Input	kg/m^3^	C	350.000	456.000	401.320	36.666	1344.418
	kg/m^3^	FA	555.000	861.000	713.268	91.209	8319.014
	kg/m^3^	CA	900.000	1227.500	1055.409	91.372	8348.753
	%	w/c	0.400	0.500	0.453	0.035	0.001
	Cells/ml	B	103.000	109.000	105.410	1.436	2.063
Output	MPa	CS	18.670	61.790	37.874	13.518	182.744
	MPa	FS	4.120	8.600	5.427	1.174	1.378
	mm	Sl	57.000	96.000	69.180	9.570	91.584

### Research plan

Linear multivariate Regression Model (LMR), Response Surface Methodology (RSM), Gray Wolf Optimization (GWO), Multi-Verse Optimization (MVO), Moth-Flame Optimization (MFO), Particle Swarm Optimization (PSO) and Whale Optimization Algorithm (WOA) were deployed to forecast the effect of bacteria concentration on the mechanical properties of medium-strength self-healing concrete (MSSHC) for the production of bio-concrete for the design and construction of green and sustainable structures. GWO, MVO, MFO, PSO, and WOA are advanced metaheuristic techniques in machine learning known for their precision, robustness and speed in model execution.

#### LMR

**Figure 4:**
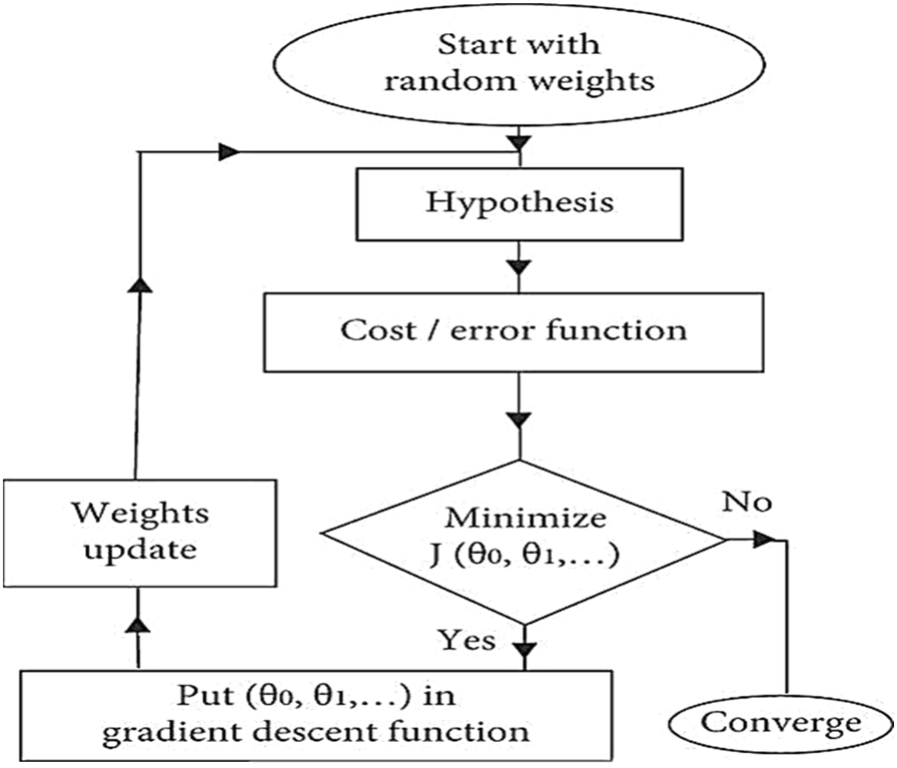
LMR flowchart.

Linear multivariate regression (LMR) is a statistical technique used to model the relationship between multiple independent variables and a dependent variable. The flowchart is illustrated in Fig. 4. In LMR, the goal is to find a linear equation that best predicts the value of the dependent variable based on the values of the independent variables. This technique is commonly used in various fields, including economics, finance, social sciences, and more, to analyze and predict the relationship between multiple variables. Linear multivariate regression is an extension of simple linear regression to multiple independent variables. In simple linear regression, we have one dependent variable and one independent variable, whereas in multivariate regression, we have one dependent variable and multiple independent variables. The goal of multivariate linear regression is to estimate the coefficients that best fit the observed data. This is typically done by minimizing the sum of squared differences between the observed and predicted values of the dependent variable. The estimation of coefficients is often done using methods like Ordinary Least Squares (OLS), where the coefficients are chosen to minimize the sum of squared residuals. Multivariate regression analysis allows us to understand the relationship between the dependent variable and multiple independent variables simultaneously. It's widely used in various fields, including economics, social sciences, engineering, and many others, for predictive modeling, hypothesis testing, and understanding the impact of independent variables on the dependent variable.

#### RSM

**Figure 5:**
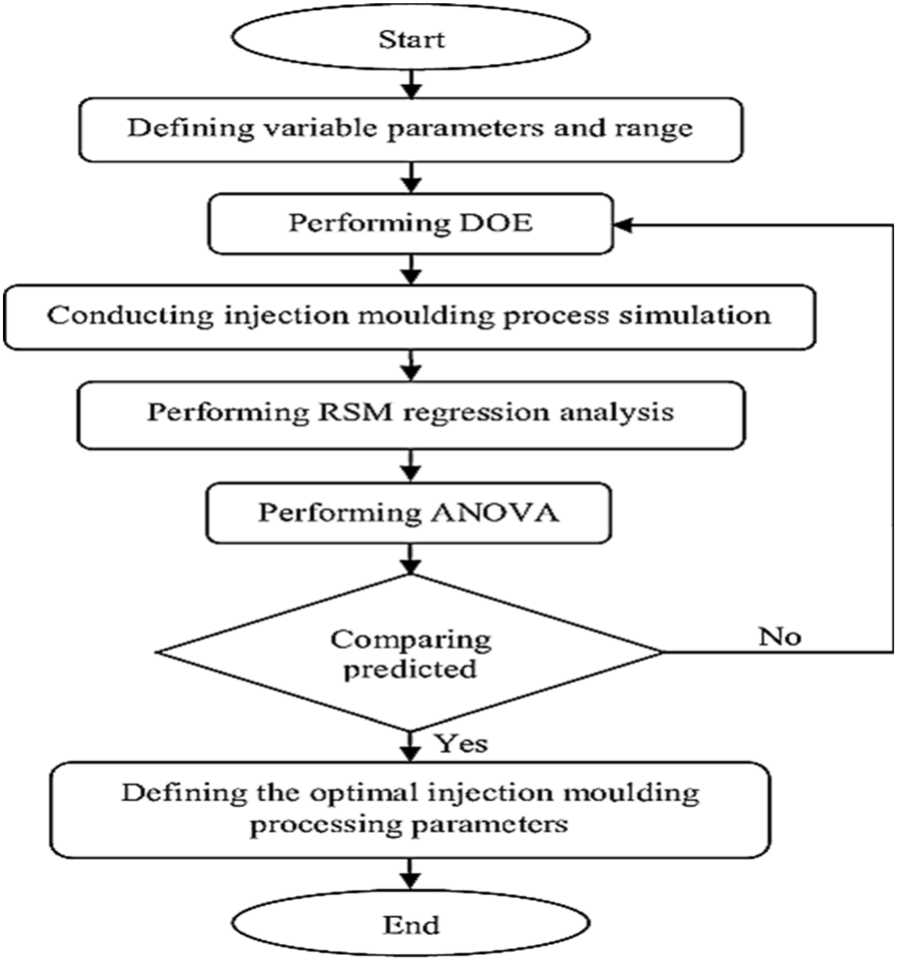
RSM flowchart.

Response Surface Methodology (RSM) is a collection of statistical and mathematical techniques used to model and analyze the relationship between a set of controlled independent variables and the observed response of a system. The flowchart is illustrated in Fig. 5. RSM is often used in the field of engineering, chemistry, and other physical sciences to optimize processes, improve product quality, and understand the interactions between input variables. One of the key features of RSM is its ability to construct and analyze mathematical models that describe the relationship between the input variables and the system response. These models can help in predicting optimal process conditions and understanding the behavior of complex systems. RSM typically involves conducting a series of experiments to systematically vary the input variables and observe the corresponding changes in the system response. The data gathered from these experiments is then analyzed to develop a predictive model that can be used to optimize the system's performance. Overall, RSM provides a systematic and efficient approach for optimizing processes and understanding the relationships between input variables and system responses.

#### GWO

**Figure 6:**
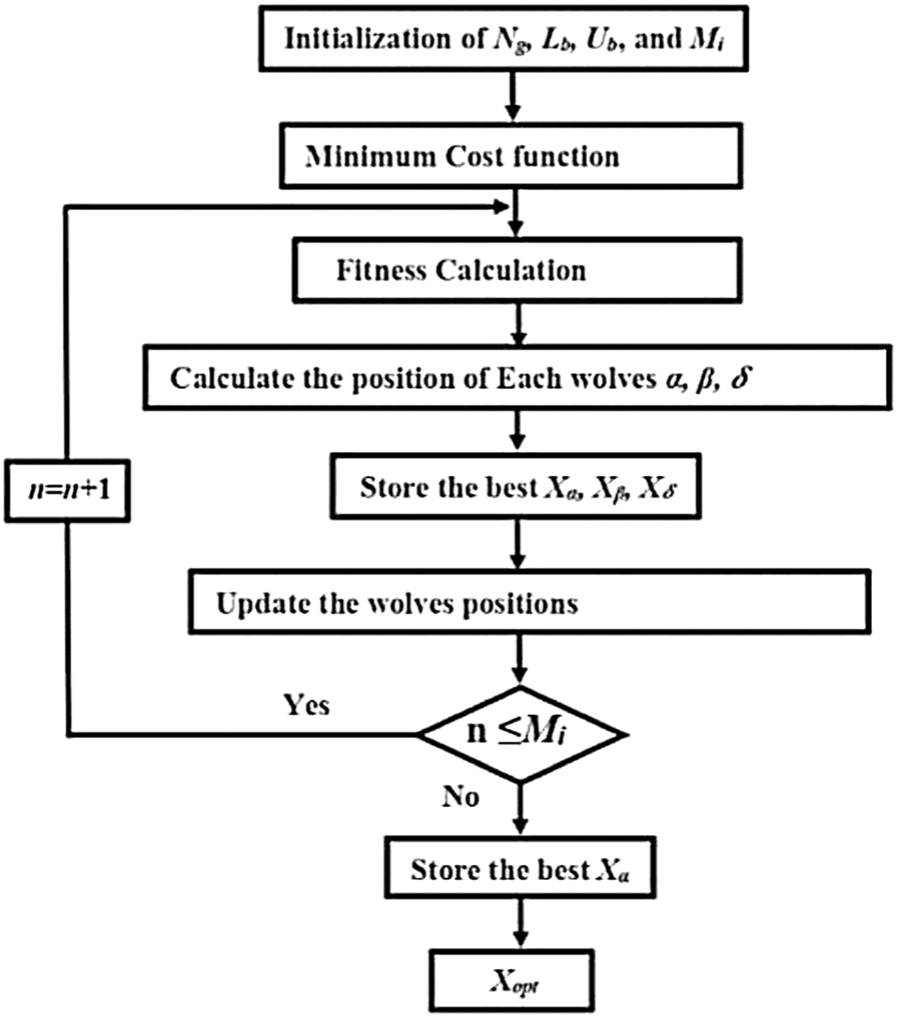
GWO flowchart.

Gray Wolf Optimization (GWO) is a nature-inspired optimization algorithm that is based on the social hierarchy and hunting behavior of gray wolves. The flowchart is illustrated in Fig. 6. It is a metaheuristic algorithm used to solve optimization problems and is inspired by the hunting and leadership hierarchy of gray wolf packs. In GWO, the population of candidate solutions is divided into four types of wolves: alpha, beta, delta, and omega. These wolves represent the best solution, the second-best solution, the third-best solution, and the worst solution, respectively. The positions of the wolves are updated iteratively based on the hunting and social behavior of the gray wolves. The algorithm involves simulating the way that a pack of wolves collaborates to hunt and track down prey, with the goal of converging towards an optimal solution. The concept of alpha, beta, delta, and omega wolves is used to guide the search process towards the best solution. GWO has been applied to various optimization problems in fields such as engineering, computer science, and finance. Its effectiveness and efficiency have made it a popular choice for solving complex optimization problems.

#### MVO

**Figure 7:**
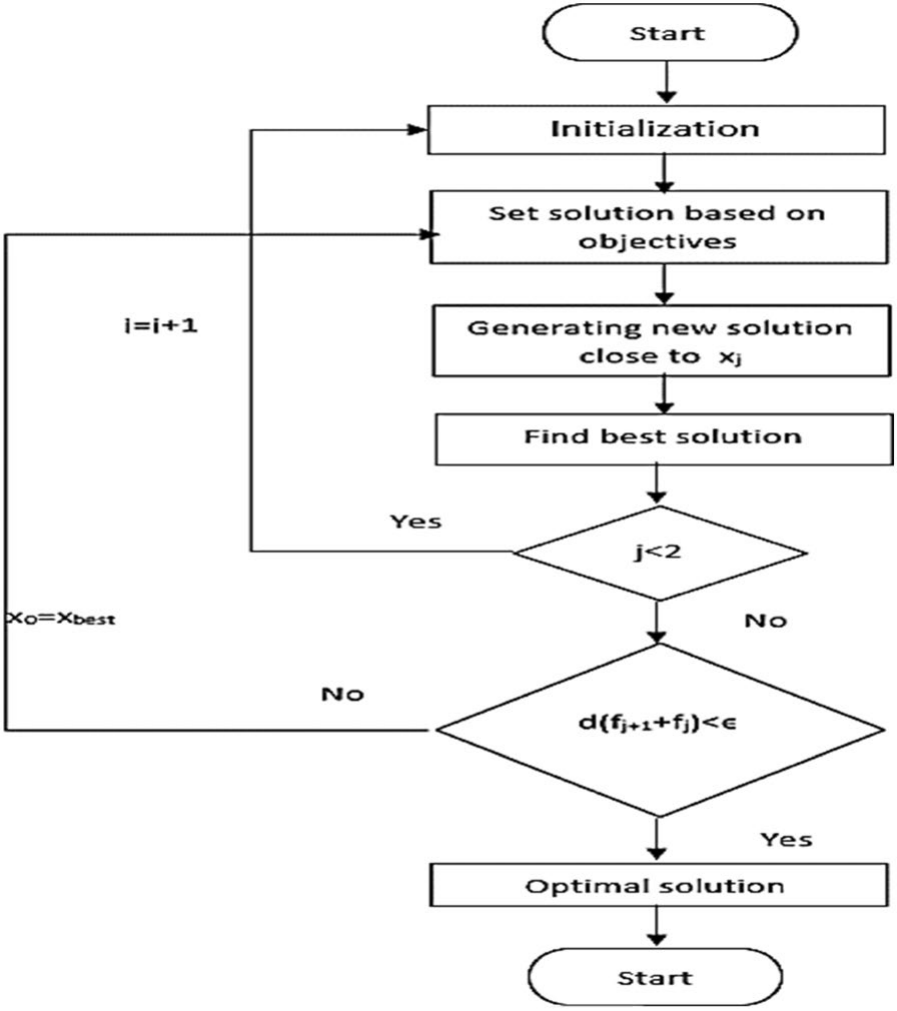
MVO flowchart.

The Multiverse Optimization Algorithm (MOA) is a relatively new metaheuristic optimization algorithm inspired by the concept of the multiverse from theoretical physics. The flowchart is illustrated in Fig. 7. It's a population-based algorithm that draws inspiration from the idea of multiple universes coexisting simultaneously, each representing a potential solution to the optimization problem. In MOA, a population of candidate solutions is represented as a collection of universes. Each universe corresponds to a potential solution to the optimization problem. These universes evolve over iterations through various operators, including expansion, contraction, and merging, which are inspired by physical phenomena such as expansion and contraction of the universe. MOA aims to explore the solution space efficiently by allowing universes to explore different regions and exchange information with each other. This exploration–exploitation balance helps in finding high-quality solutions to optimization problems across a wide range of domains. The algorithm's key components include: Initialization: Generating an initial population of universes (candidate solutions) randomly or using some heuristic. Evaluation: Assessing the fitness of each universe (solution) in the population based on the objective function of the optimization problem. Evolutionary Operators: Applying operators inspired by physical phenomena to evolve the universes over generations. These operations include expansion, contraction, and merging. Selection: Selecting universes for the next generation based on their fitness. This could involve strategies like elitism or stochastic selection. Termination Criteria: Determining when to stop the algorithm, usually based on reaching a maximum number of iterations, finding a satisfactory solution, or exhausting computational resources. MOA has been applied to various optimization problems, including continuous, discrete, and combinatorial optimization tasks. Its effectiveness depends on parameter settings, problem characteristics, and tuning strategies. While it's not guaranteed to find the global optimum, MOA often provides competitive results compared to other metaheuristic algorithms.

#### MFO

**Figure 8:**
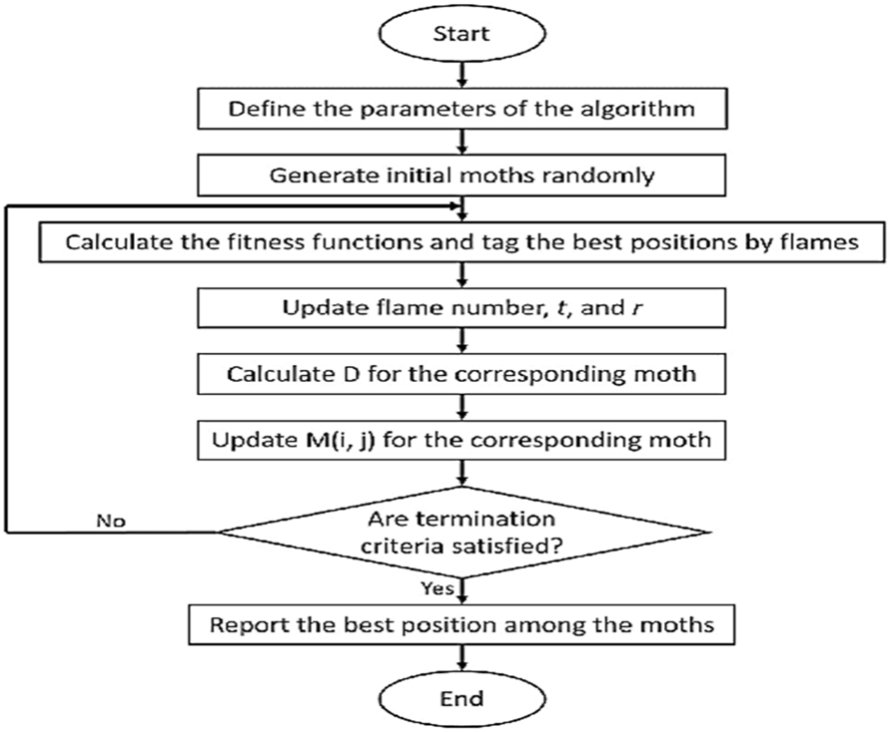
MFO flowchart.

Moth Flame Optimization (MFO) is a nature-inspired metaheuristic optimization algorithm inspired by the behavior of moths in the presence of a flame. The flowchart is illustrated in Fig. 8. It was proposed as a population-based optimization algorithm by Xin-She Yang in 2018. The basic idea of MFO lies in mimicking the behavior of moths when they are attracted to a flame. Moths exhibit a behavior of moving towards the light source while keeping a certain distance to avoid being burned. This behavior forms the basis of the algorithm's exploration and exploitation strategy. Here's a simplified overview of how MFO works: Initialization: Randomly initialize a population of moths (solutions) in the search space. Attraction to Light: Moths are attracted to the brightest light source, representing a high-quality solution in the search space. The brightness of the light source is determined by the fitness of the solutions. Flight towards the Light: Each moth adjusts its position based on the position of the brightest light source (i.e., the best solution found so far) while maintaining a certain distance to avoid being too close to the light source (to prevent convergence to local optima). Moth Encirclement: Some moths may move too close to the light source. To prevent premature convergence, a fraction of the moths are selected for encirclement, where they are forced to move randomly to explore new regions of the search space. Updating Light Intensity: The intensity of the light source (fitness of the best solution) may decrease over iterations to simulate the diminishing attractiveness of the light source as moths gather around it. Termination Criteria: The algorithm stops when a termination condition is met, such as reaching a maximum number of iterations or finding a satisfactory solution. MFO has been applied to various optimization problems, including both continuous and discrete optimization tasks. Like other metaheuristic algorithms, its performance depends on parameter settings, problem characteristics, and tuning strategies. While it may not guarantee finding the global optimum, MFO often provides competitive results and can be particularly effective for certain types of problems.

#### PSO

**Figure 9:**
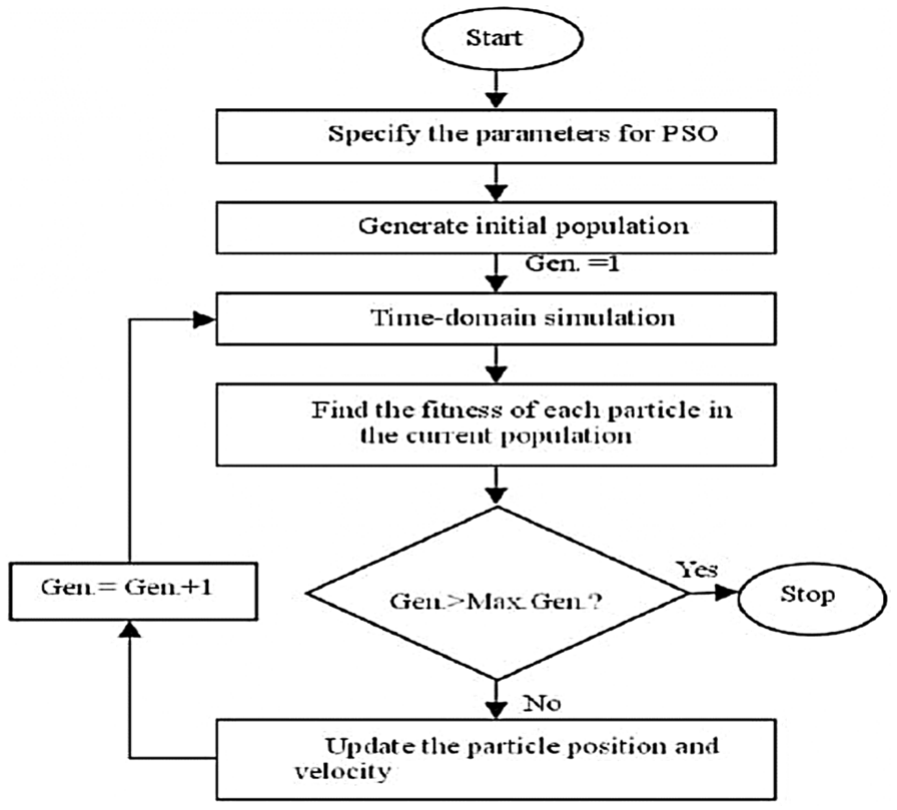
PSO flowchart.

Particle Swarm Optimization (PSO) is a population-based stochastic optimization algorithm inspired by the social behavior of birds flocking or fish schooling. The flowchart is illustrated in Fig. 9. It was originally developed by Dr. Eberhart and Dr. Kennedy in 1995 and has since become a popular optimization technique used to solve a wide range of problems in various fields. In PSO, the potential solutions to an optimization problem, called particles, are treated as a swarm. Each particle adjusts its position in the search space according to its own flying experience as well as the flying experiences of other particles in the swarm. The movement of particles is influenced by their own best-known position and the best-known position in the entire swarm. The algorithm iteratively improves the candidate solutions by adjusting the velocity and position of each particle based on its own experience and the experience of its neighbors. As the iterations progress, the particles move through the search space, gradually converging towards the optimal solutions. PSO has been widely applied in fields such as engineering, computer science, finance, and many others, to solve optimization problems, including function optimization, neural network training, and feature selection, among others. Its simplicity, ease of implementation, and effectiveness in finding near-optimal solutions have contributed to its popularity.

#### WOA

**Figure 10:**
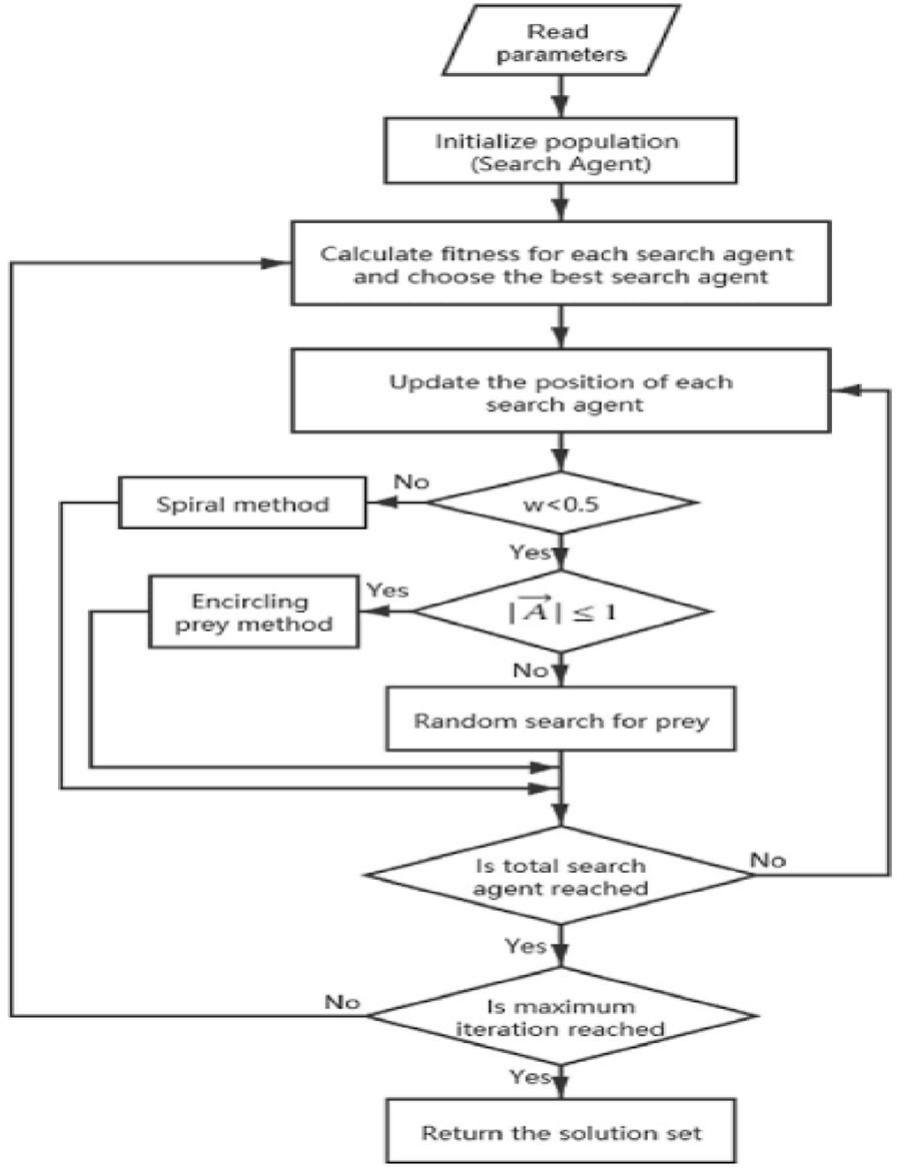
WOA flowchart.

The Whale Optimization Algorithm (WOA) is a nature-inspired optimization algorithm proposed by Seyedali Mirjalili in 2016. The flowchart is illustrated in Fig. 10. It is inspired by the social behavior of humpback whales during hunting. In the Whale Optimization Algorithm, the search process is modeled after the bubble-net feeding behavior of humpback whales. The algorithm mimics the hunting behavior of whales and their communication to encircle and catch prey. WOA operates by iteratively updating the position of a population of candidate solutions, representing potential prey, based on the behavior of virtual whales. The key features of the WOA include the utilization of exploration and exploitation phases to balance global and local search, and the use of mathematical equations that imitate the encircling behavior of whales. WOA has been applied to solve optimization problems in various domains, including engineering, data science, and other fields. Its effectiveness and ability to quickly converge to near-optimal solutions have made it a subject of interest for researchers and practitioners in the field of optimization. WOA is one of several nature-inspired optimization algorithms that draw inspiration from natural phenomena to develop efficient optimization techniques.

#### Performance evaluation

The models are further tested for run efficiency by using selected error metrics as follows; the coefficient of determination (R^2^), root mean squared errors (RMSE), mean absolute errors (MAE), mean squared errors (MSE), variance accounted for (VAF) and the coefficient of error (CE). The mathematical expressions of the validation indices are presented in Eqs. (2–7).

2 $$ R^{2} = 1 - \frac{{\mathop \sum \nolimits_{i = 1}^{N} \left( {X_{i - \Pr edicted} - X_{i - Measured} } \right)^{2} }}{{\mathop \sum \nolimits_{i = 1}^{N} \left( {X_{Mean - Measured} - X_{i - Measured} } \right)^{2} }} $$

3 $$ RMSE = \sqrt {\frac{{\mathop \sum \nolimits_{i = 1}^{N} \left( {X_{i - \Pr edicted} - X_{i - Measured} } \right)^{2} }}{N}} $$

4 $$ MAE = \frac{{\mathop \sum \nolimits_{i = 1}^{N} \left| {X_{i - \Pr edicted} - X_{i - Measured} } \right|}}{N} $$

5 $$ {\text{MSE}} = \frac{1}{N}\mathop \sum \limits_{i = 1}^{N} \left( {X_{i - \Pr edicted} - X_{i - Measured} } \right)^{2} $$

6 $$ VAF = \left( {1 - \frac{{VAR\left( {X_{\Pr edicted} - X_{Measured} } \right)}}{{VAR\left( {X_{Measured} } \right)}}} \right) $$

7 $$ CE = 1 - \frac{{\mathop \sum \nolimits_{k = 1}^{N} \left( {X_{predicted} - X_{Measured} } \right)^{2} }}{{\mathop \sum \nolimits_{k = 1}^{N} \left( {X_{predicted} - \overline{{X_{predicted} }} } \right)^{2} }} $$

## Results and analysis

### Response surface methodology analysis for the compressive strength

**Figure 11:**
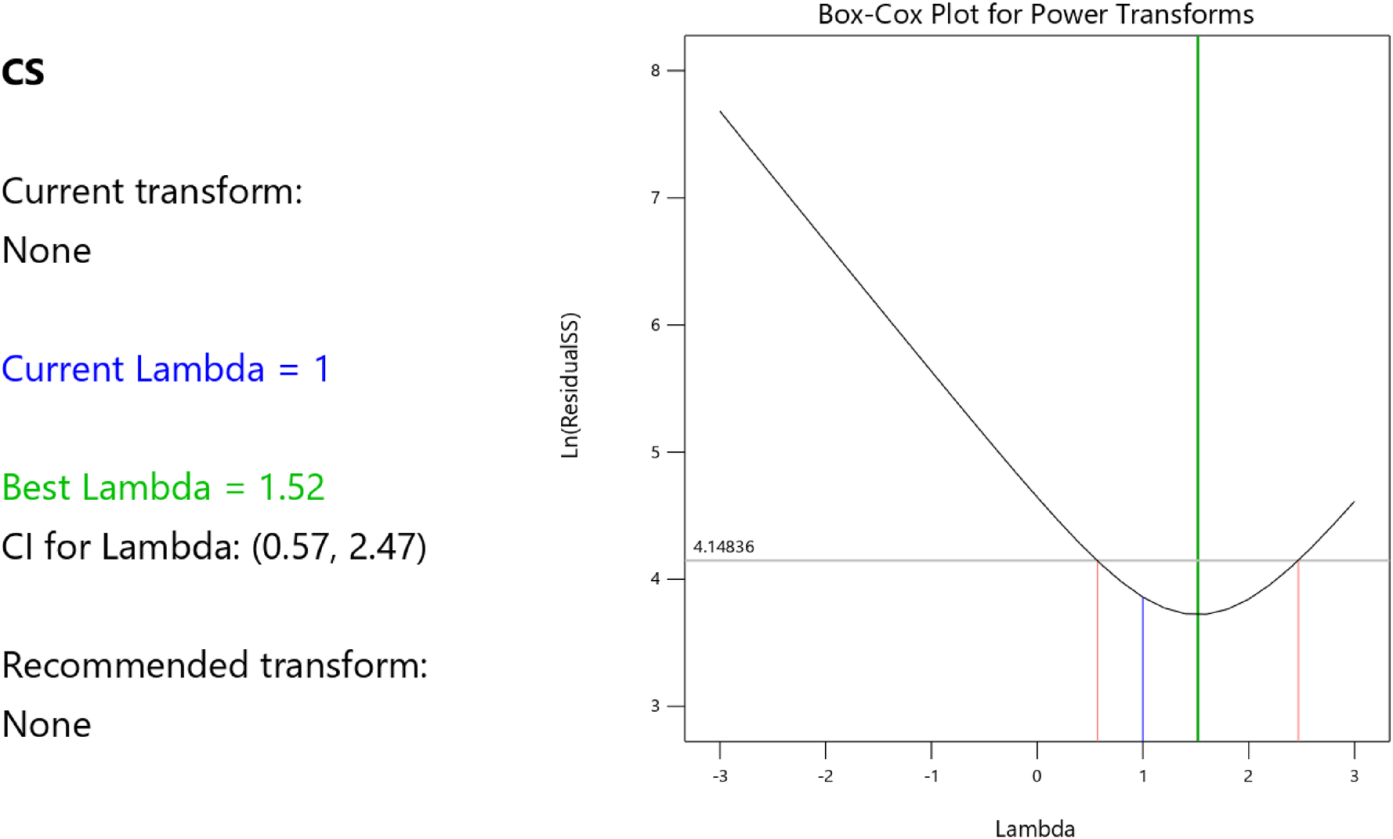
Compressive strength box-cox model plot.

**Figure 12:**
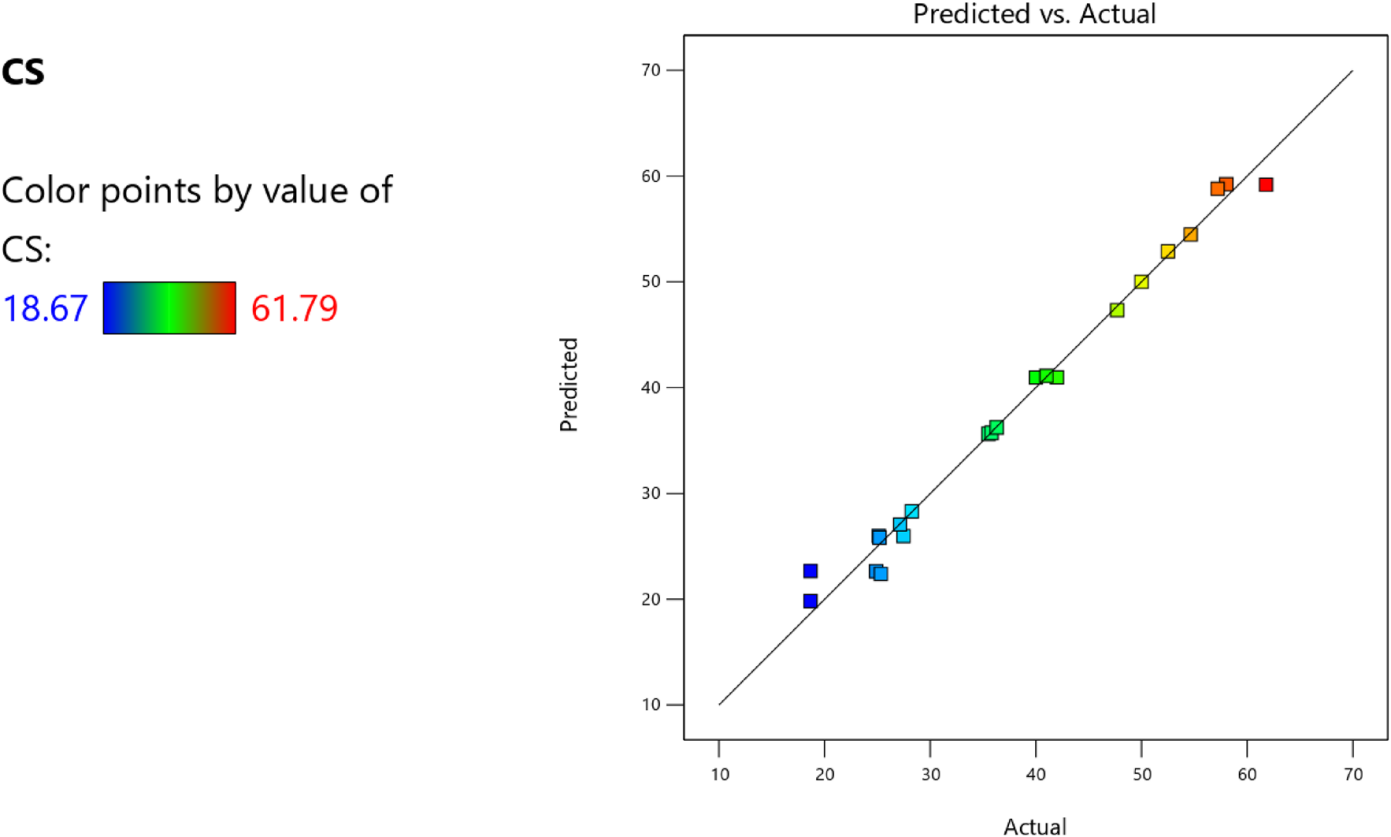
Compressive strength predicted versus actual scatter plot.

**Figure 13:**
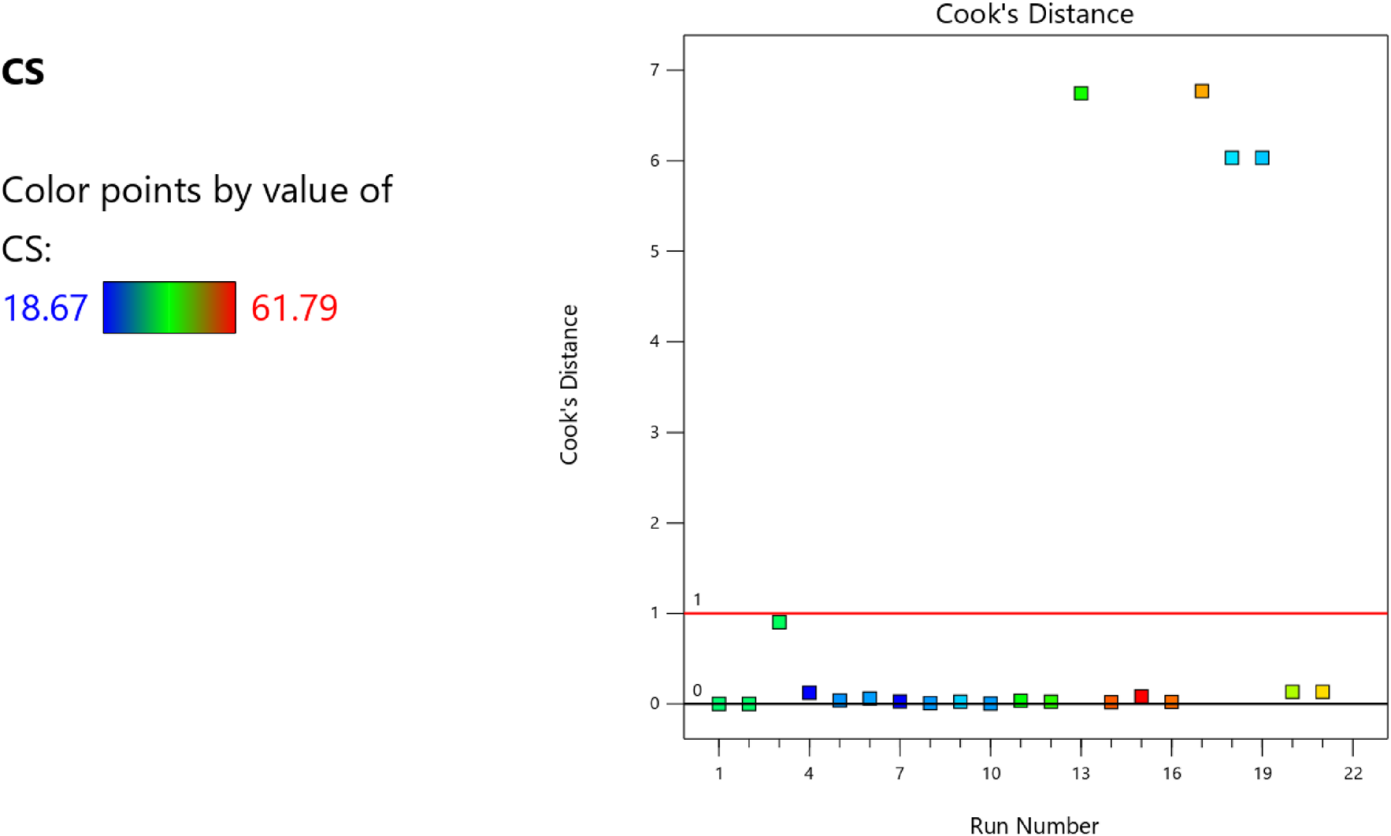
Compressive strength model cook’s distance.

**Figure 14:**
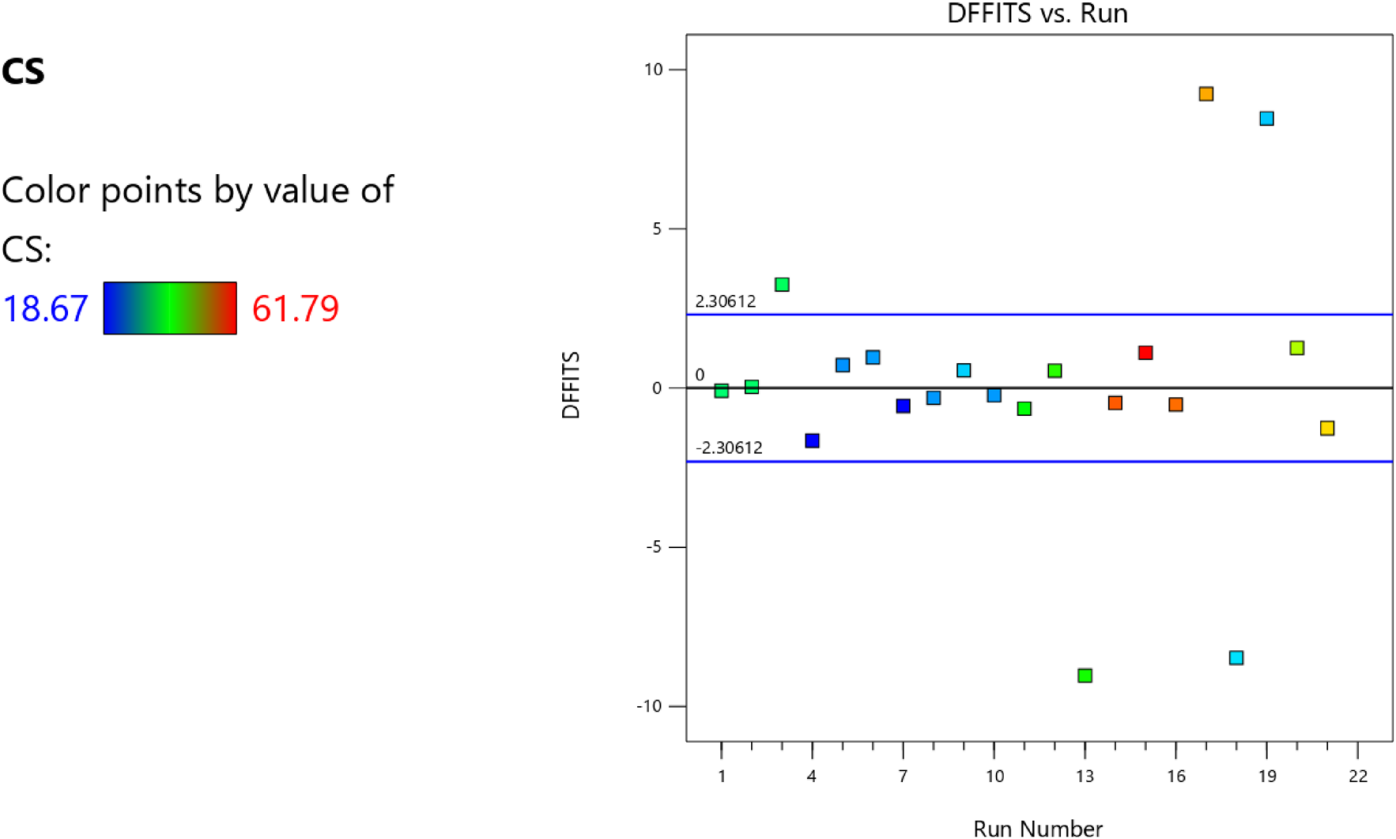
Compressive strength model DFFITS versus run plot.

**Figure 15:**
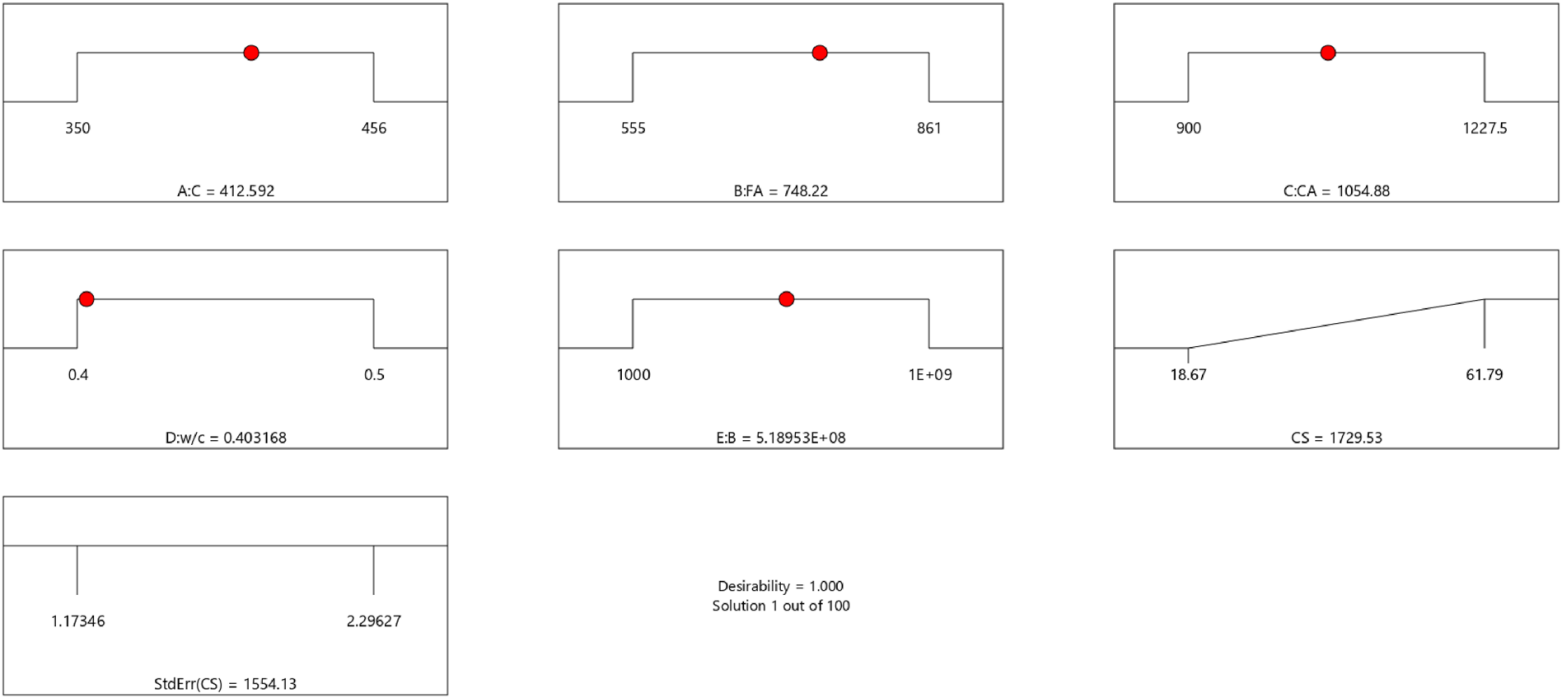
Desirability level of the selected optimized solution.

**Figure 16:**
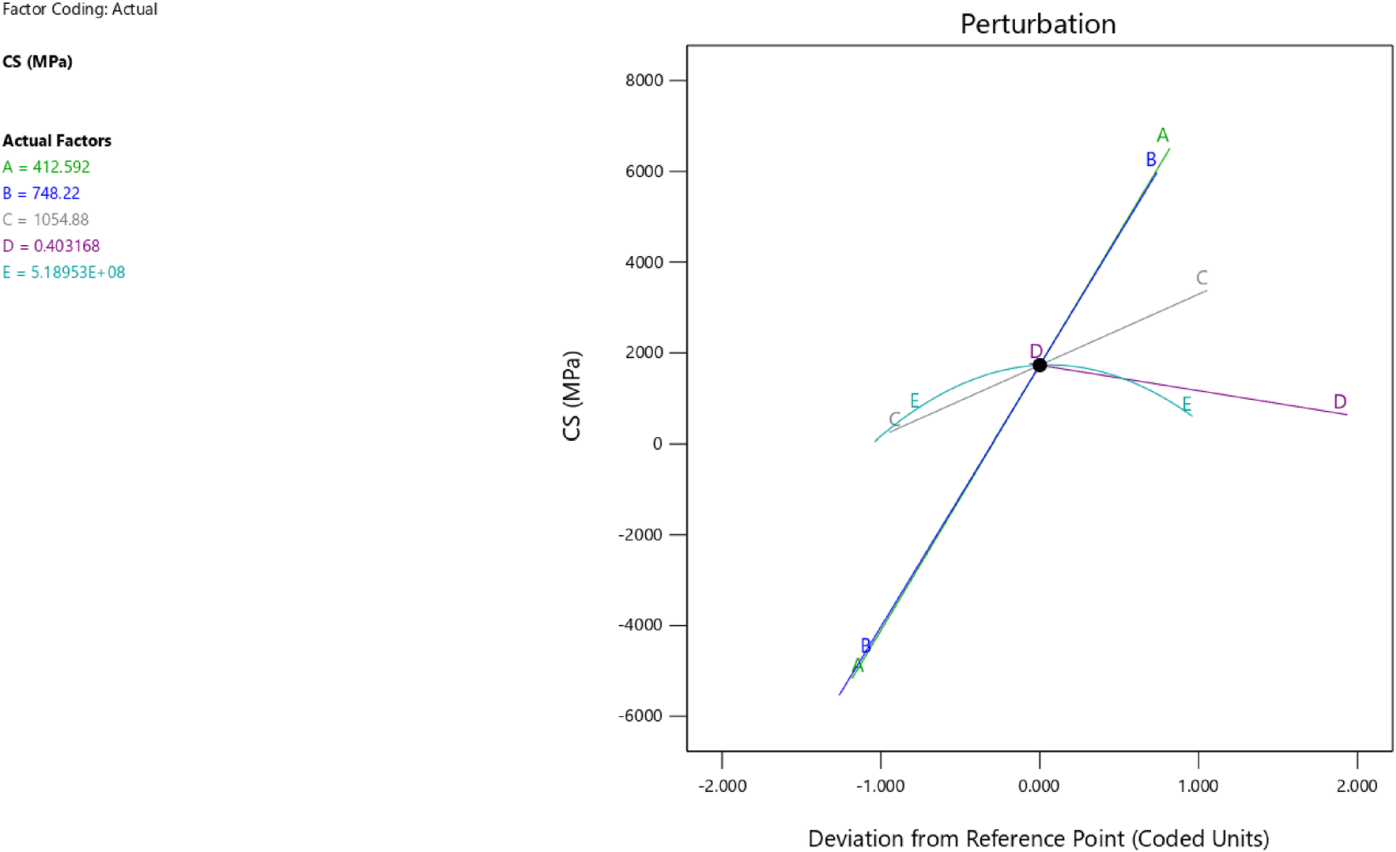
Compressive strength model perturbation.

**Figure 17:**
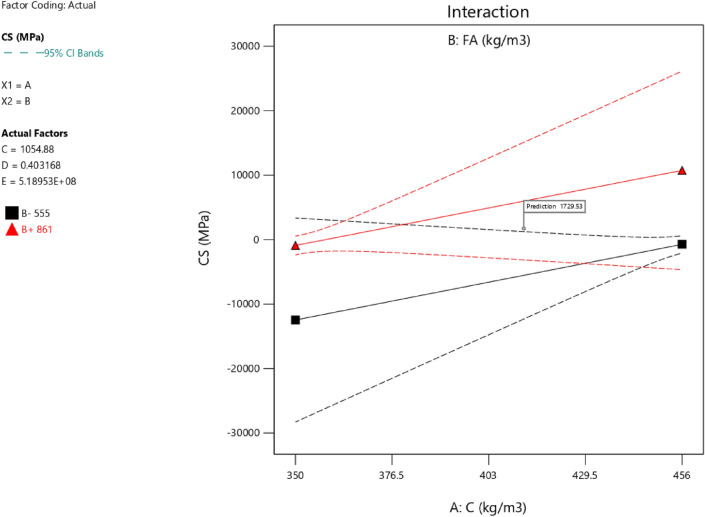
Compressive strength model interaction between the parameters.

**Figure 18:**
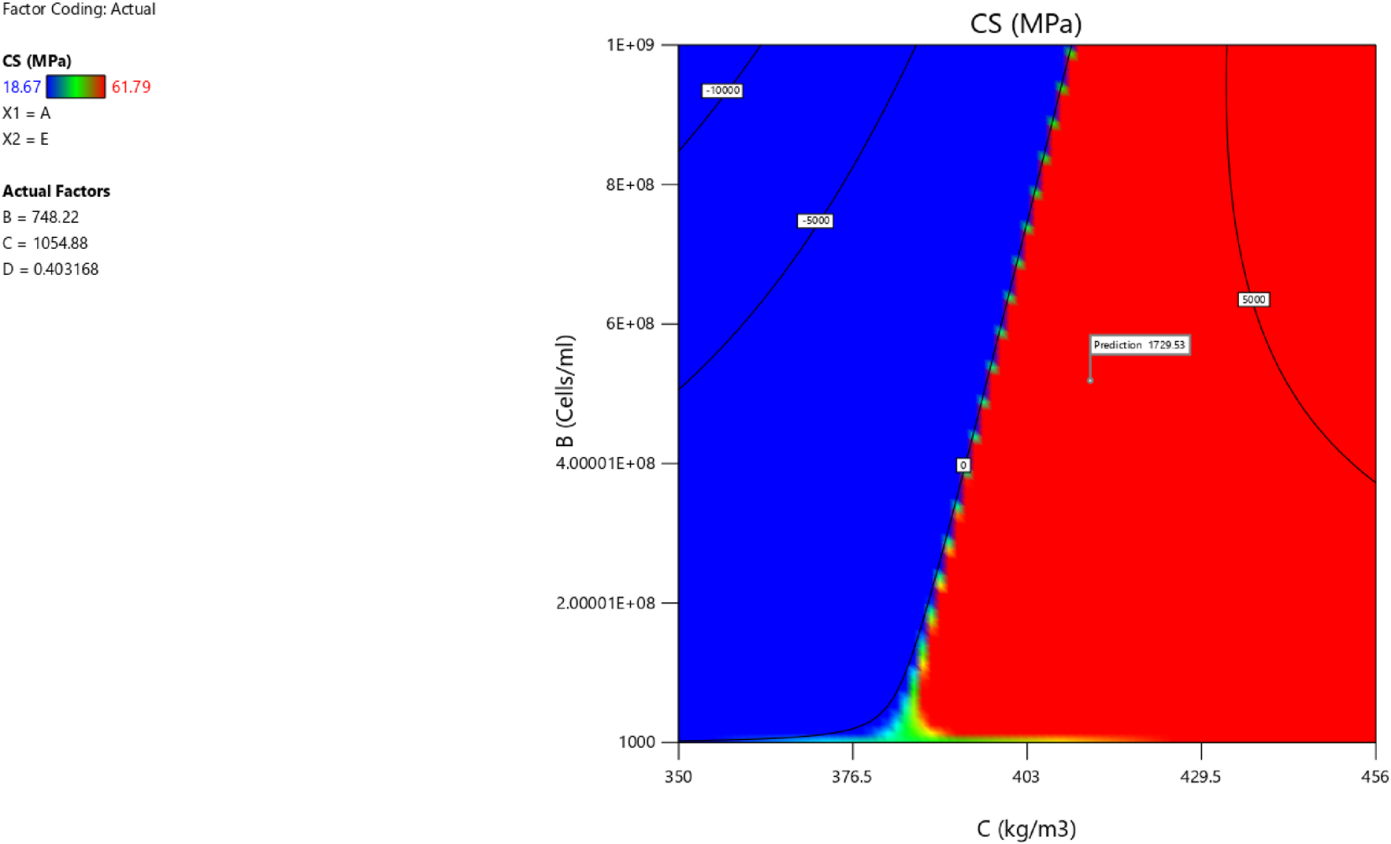
Compressive strength model contour for the concrete mixtures.

**Figure 19:**
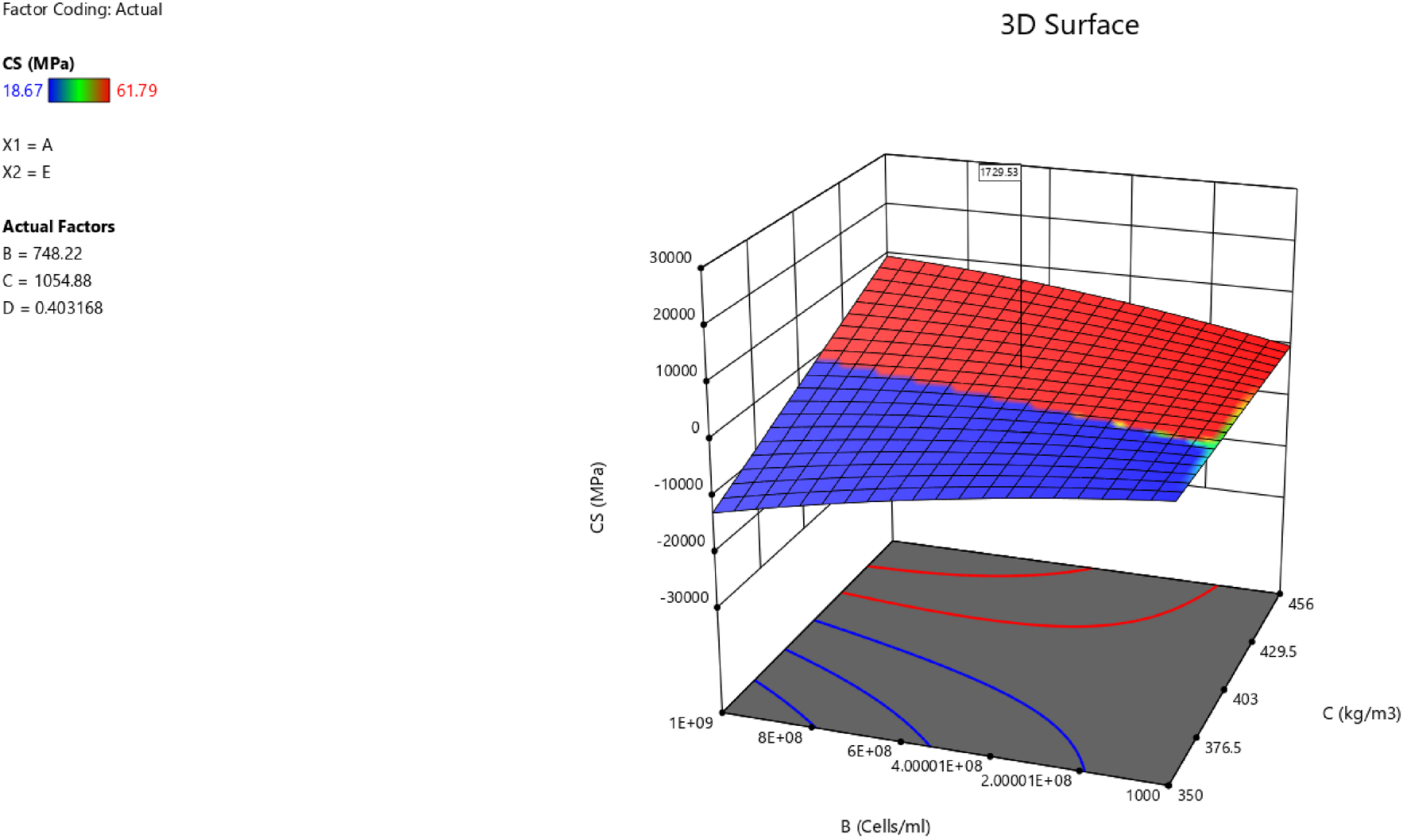
3D configuration of the compressive strength model.

Factor coding is actual. Sum of squares is Type III—Partial. The Model F-value of 59.90 implies the model is significant. There is only a 0.01% chance that an F-value this large could occur due to noise. *P*-values less than 0.0500 indicate model terms are significant. In this case A, B, C, D, AB are significant model terms. Values greater than 0.1000 indicate the model terms are not significant. If there are many insignificant model terms (not counting those required to support hierarchy), model reduction may improve your model. The Lack of Fit F-value of 0.25 implies the Lack of Fit is not significant relative to the pure error. There is an 89.82% chance that a Lack of Fit F-value this large could occur due to noise. Non-significant lack of fit is good – we want the model to fit. Adeq Precision measures the signal to noise ratio. A ratio greater than 4 is desirable. Your ratio of 22.320 indicates an adequate signal. This model can be used to navigate the design space. These can be read out from Tables 2 and 3, and Figs. 11, 12, 13 and 14. The constraints of the RSM model and the selected solution from the 100 iterations are presented in Tables 4 and 5. The desirability of the optimized compressive strength, color contour configurations and the response surface optimized configuration are presented in Figs. 15, 16, 17, 18 and 19.

**Table 2:** **CS** ANOVA for Quadratic model (Aliased).

Source	Sum of Squares	df	Mean Square	F-value	*p*-value	
Model	3790.18	12	315.85	59.90	< 0.0001	Significant
A-C	265.72	1	265.72	50.39	< 0.0001	
B-FA	601.96	1	601.96	114.16	< 0.0001	
C-CA	47.23	1	47.23	8.96	0.0151	
D-w/c	83.99	1	83.99	15.93	0.0032	
E-B	12.17	1	12.17	2.31	0.1631	
AB	827.32	1	827.32	156.90	< 0.0001	
AC	24.30	1	24.30	4.61	0.0604	
AD	0.0000	0				
AE	15.20	1	15.20	2.88	0.1238	
BC	0.0000	0				
BD	0.0000	0				
BE	14.31	1	14.31	2.71	0.1339	
CD	0.0000	0				
CE	3.41	1	3.41	0.6474	0.4418	
DE	10.35	1	10.35	1.96	0.1946	
A^2^	0.0000	0				
B^2^	0.0000	0				
C^2^	0.0000	0				
D^2^	0.0000	0				
E^2^	20.35	1	20.35	3.86	0.0810	
Residual	47.46	9	5.27			
Lack of fit	7.90	4	1.98	0.2498	0.8982	Not significant
Pure error	39.55	5	7.91			
Cor total	3837.63	21

**Table 3:** Fit Statistics for the compressive strength model.

SD	2.30	R^2^	0.9876
Mean	37.87	Adjusted R^2^	0.9711
C.V. %	6.06	Predicted R^2^	NA
		Adeq Precision	22.3200

**Table 4:** Constraints of compressive strength model.

Name	Goal	Lower Limit	Upper Limit	Lower Weight	Upper Weight	Importance
A:C	Is in range	350	456	1	1	3
B:FA	Is in range	555	861	1	1	3
C:CA	Is in range	900	1227.5	1	1	3
D:w/c	Is in range	0.4	0.5	1	1	3
E:B	Is in range	1000	1E+09	1	1	3
CS	Maximize	18.67	61.79	1	1	3
StdErr(CS)	None	1.17346	2.29627	1	1	3

**Table 5:** Selected solution out of the 100 solutions found.

Number	C	FA	CA	w/c	B	CS	StdErr (CS)	Desirability	
1	412.592	748.220	1054.878	0.403	518,953,024.749	1729.526	1554.133	1.000	Selected

### Response surface methodology analysis for the flexural strength

**Figure 20:**
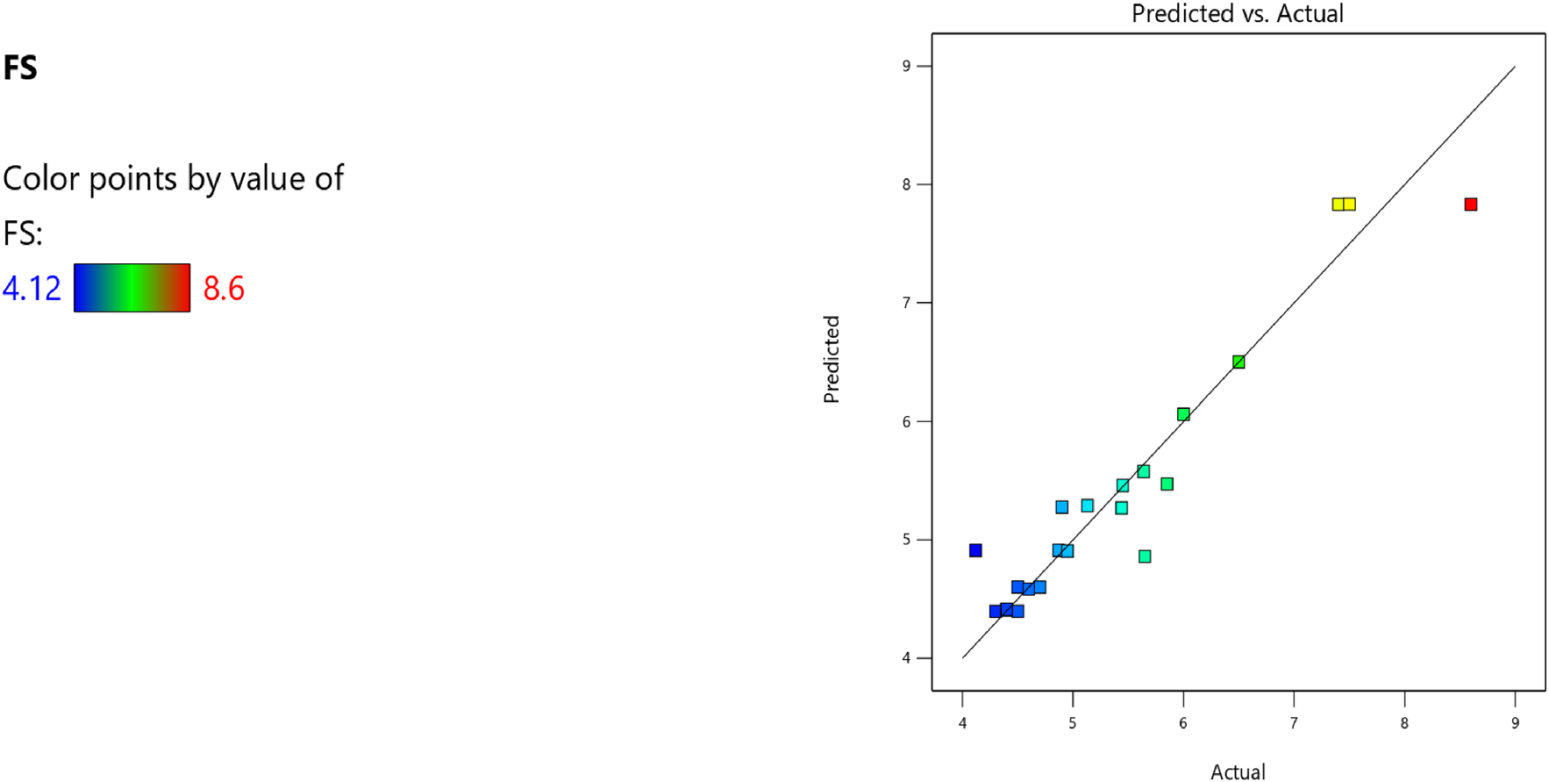
Flexural strength model predicted versus actual scatter plot.

**Figure 21:**
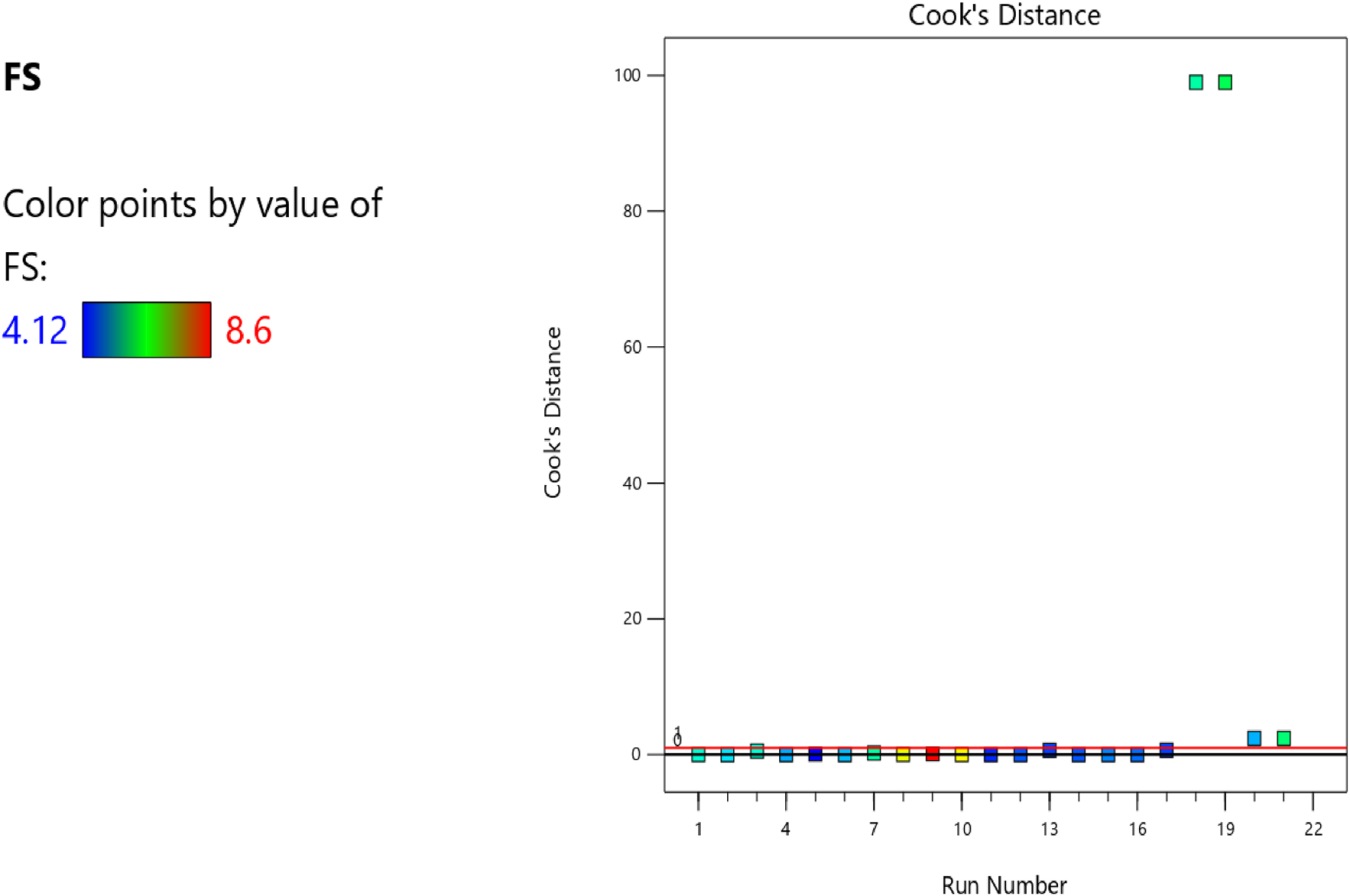
Flexural strength model cook’s distance scatter plot.

**Figure 22:**
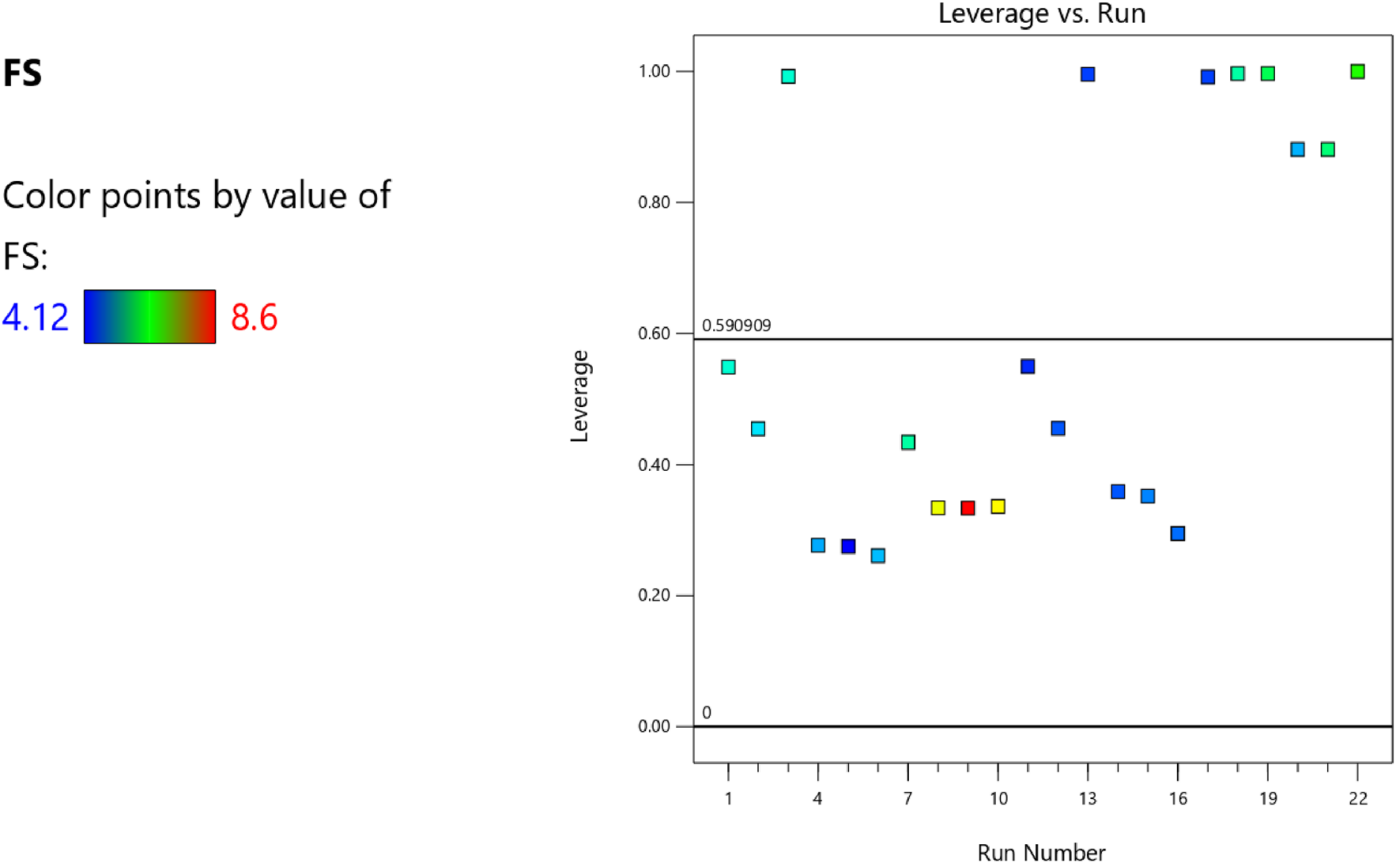
Flexural strength model leverage versus run scatter plot.

**Figure 23:**
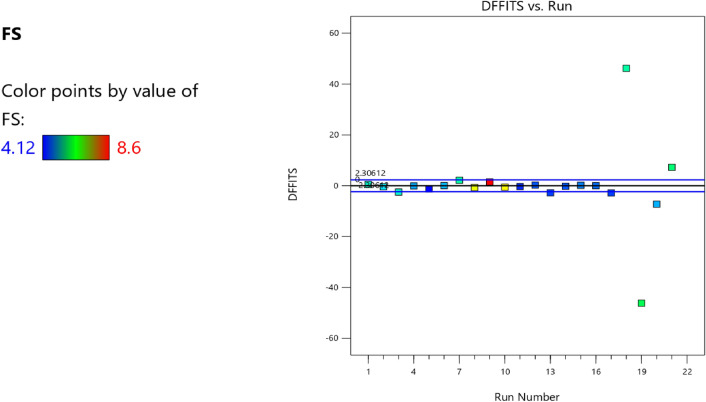
Flexural strength model DFFITS versus run scatter plot.

**Figure 24:**
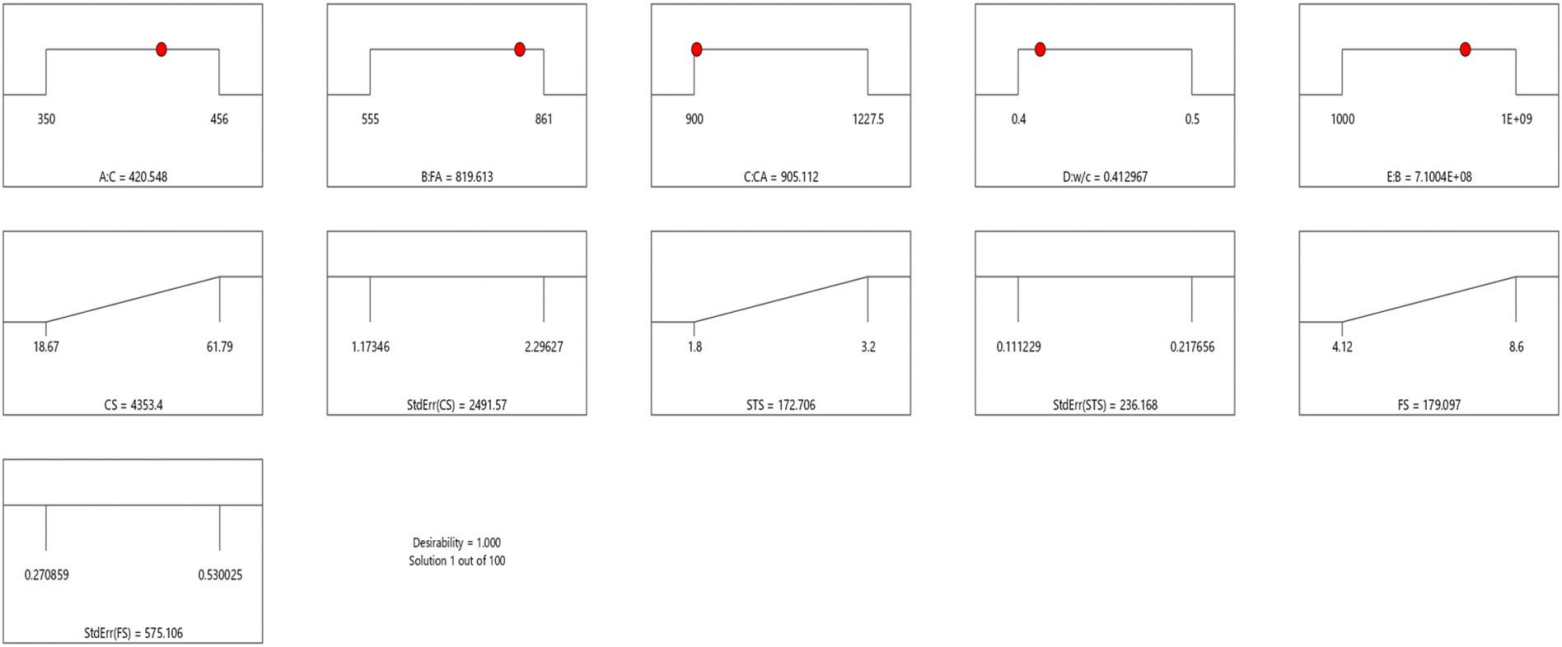
Flexural strength model desirability optimized plot.

**Figure 25:**
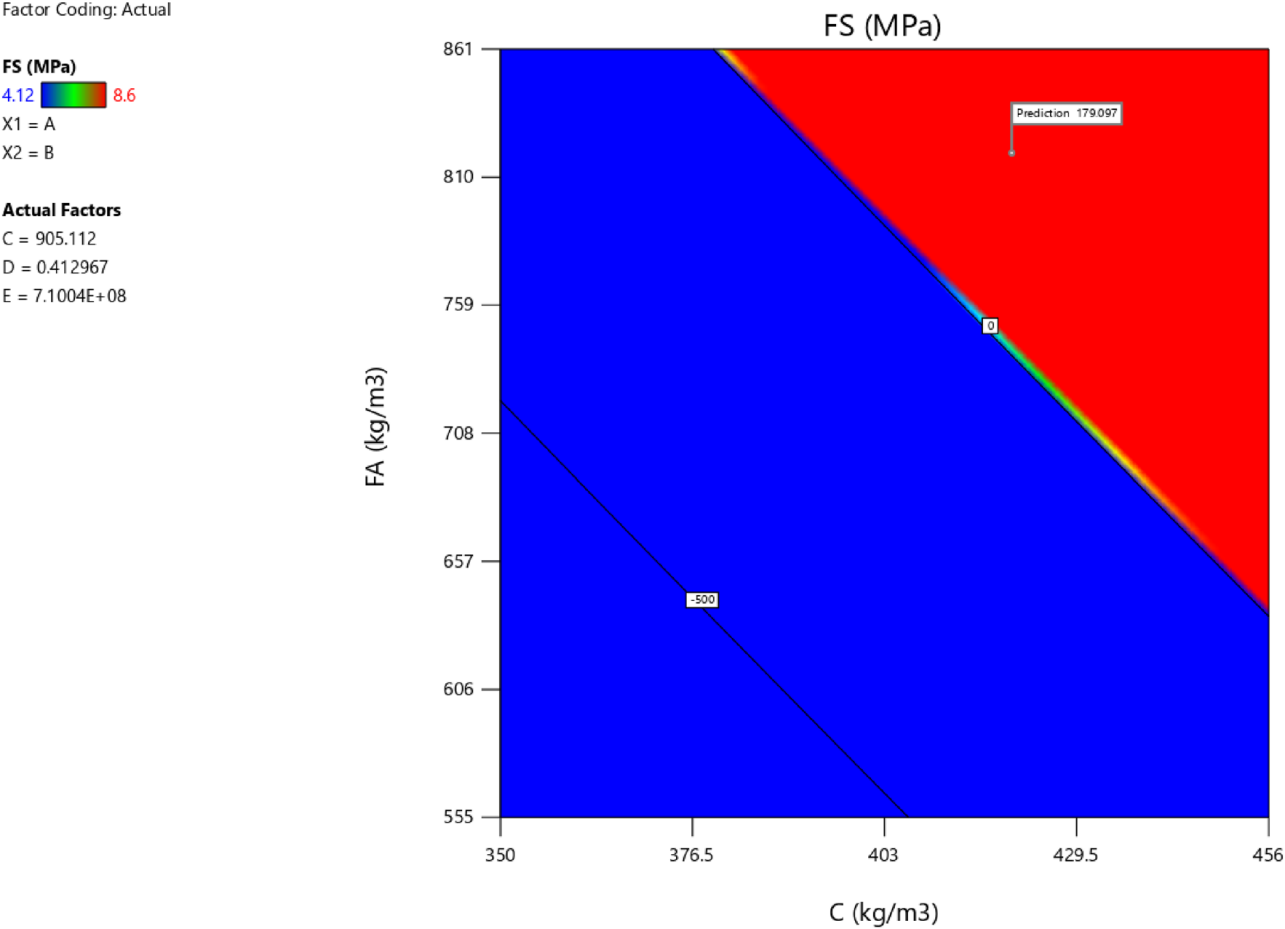
Flexural strength model contour configuration plot.

**Figure 26:**
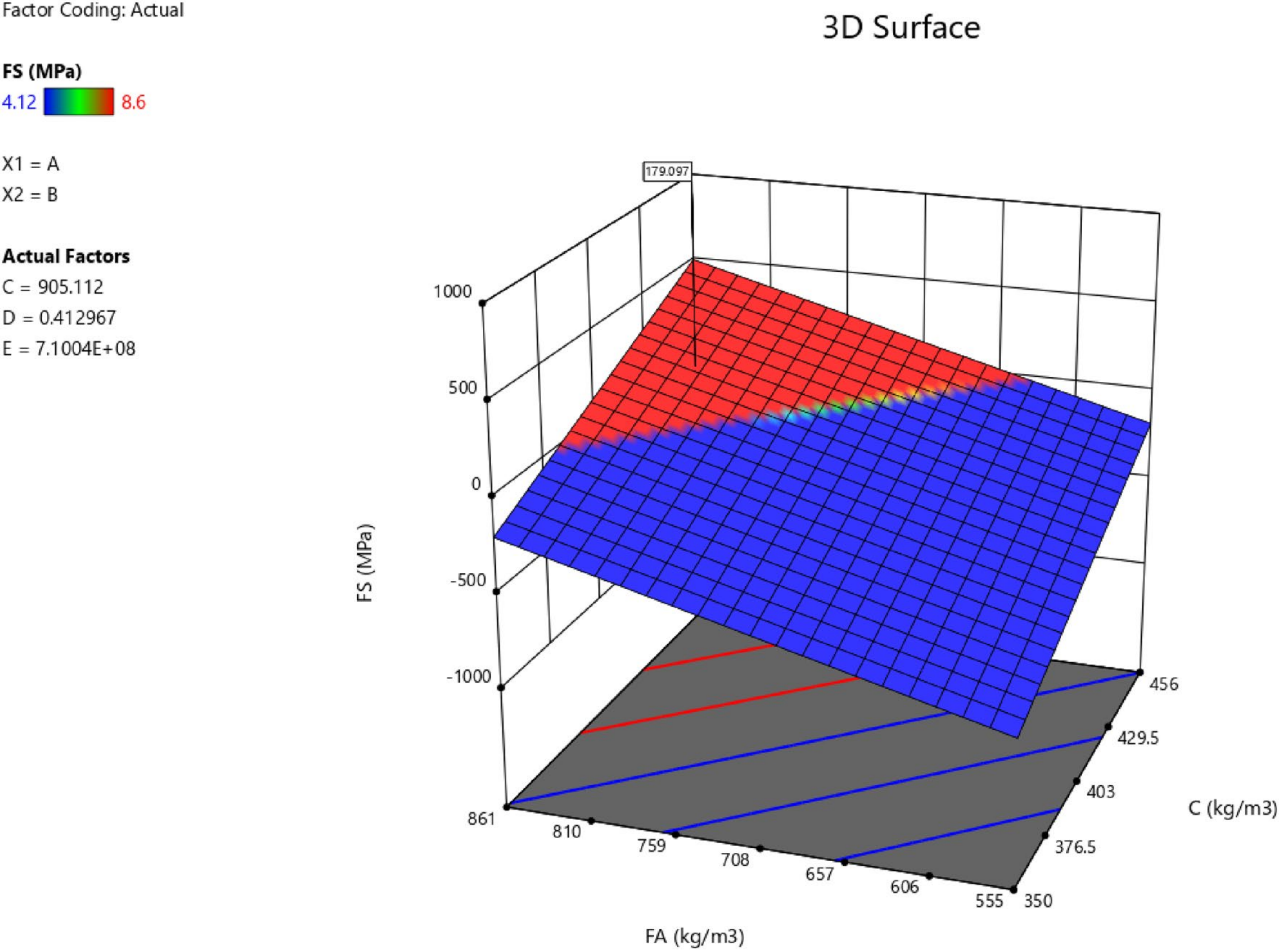
Flexural strength model 3D configuration plot.

**Figure 27:**
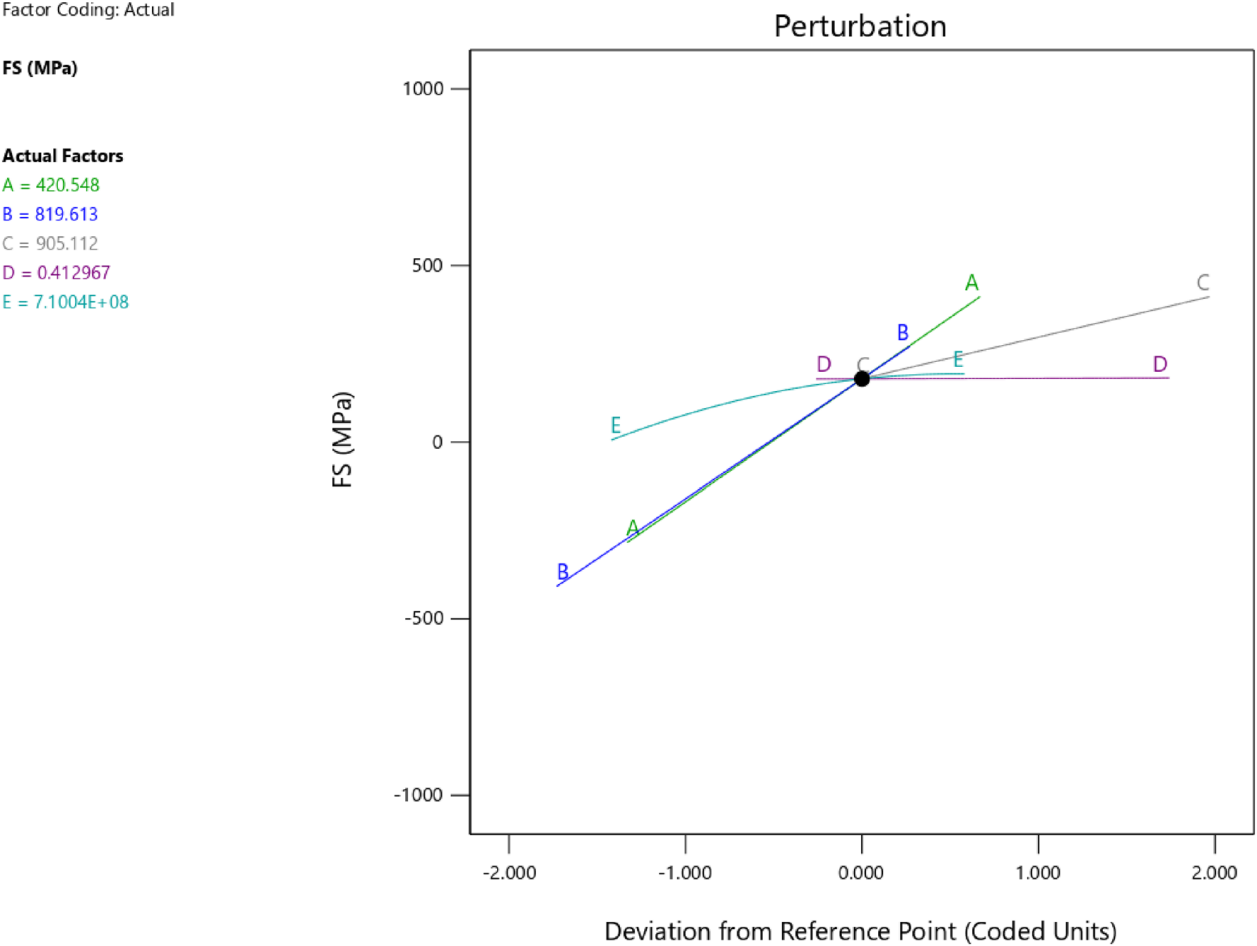
Flexural strength model perturbation configuration plot.

**Figure 28:**
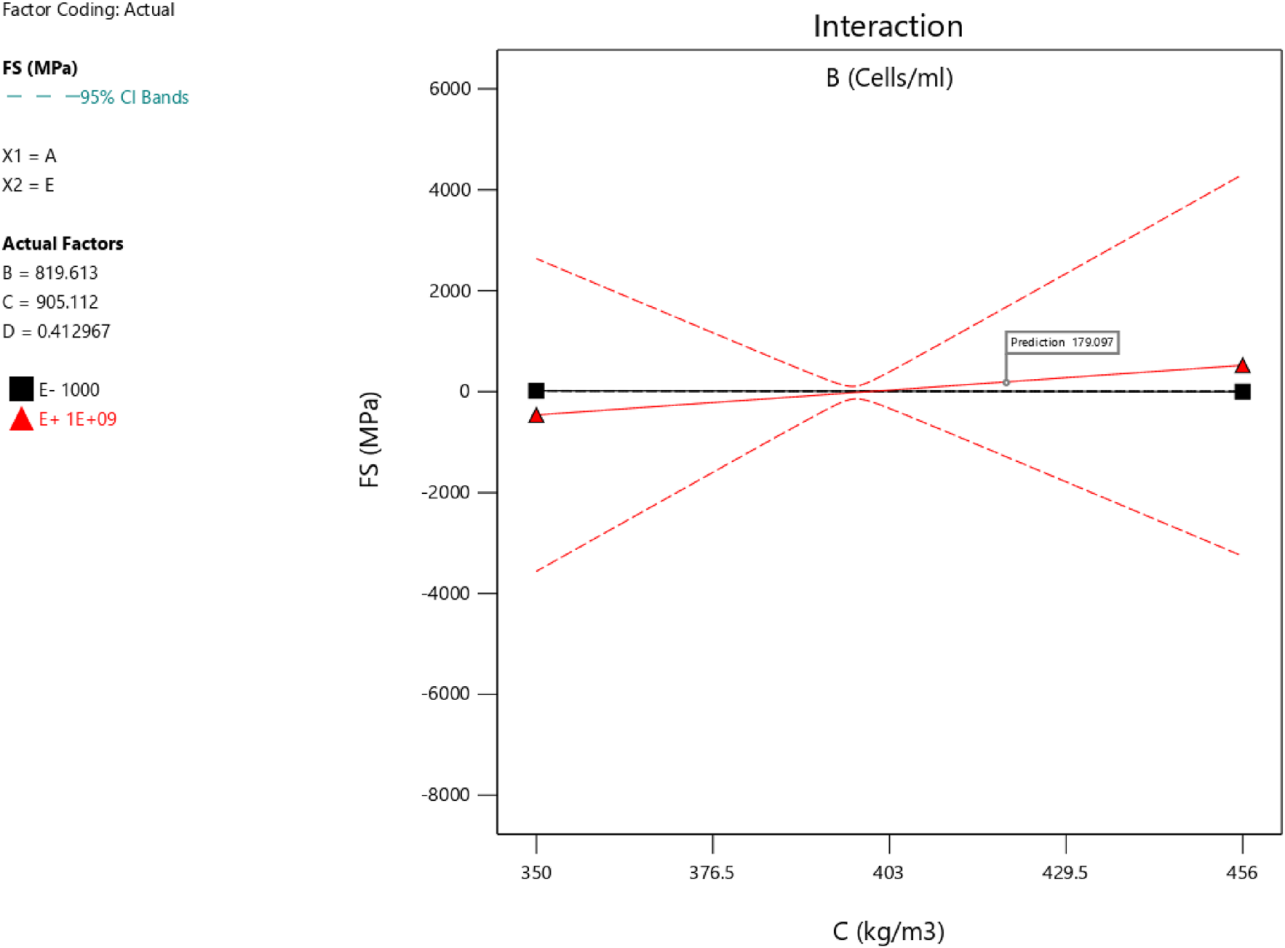
Flexural strength model interaction configuration plot.

Factor coding is actual. Sum of squares is type III—partial. The model F-value of 7.83 implies the model is significant. There is only a 0.22% chance that an F-value this large could occur due to noise. *P*-values less than 0.0500 indicate model terms are significant. In this case A, B, C, D, AB, AC are significant model terms. Values greater than 0.1000 indicate the model terms are not significant. If there are many insignificant model terms (not counting those required to support hierarchy), model reduction may improve your model. The lack of fit F-value of 1.43 implies the Lack of Fit is not significant relative to the pure error. There is a 34.68% chance that a Lack of Fit F-value this large could occur due to noise. Non-significant lack of fit is good—we want the model to fit. Adeq precision measures the signal to noise ratio. A ratio greater than 4 is desirable. Your ratio of 8.440 indicates an adequate signal. This model can be used to navigate the design space. The coefficient estimate represents the expected change in response per unit change in factor value when all remaining factors are held constant. The intercept in an orthogonal design is the overall average response of all the runs. The coefficients are adjustments around that average based on the factor settings. When the factors are orthogonal the VIFs are 1; VIFs greater than 1 indicate multi-colinearity, the higher the VIF the more severe the correlation of factors. As a rough rule, VIFs less than 10 are tolerable. The equation in terms of actual factors can be used to make predictions about the response for given levels of each factor. Here, the levels should be specified in the original units for each factor. This equation should not be used to determine the relative impact of each factor because the coefficients are scaled to accommodate the units of each factor and the intercept is not at the center of the design space. The above analyses are presented in Tables 6 and 7, Figs. 20, 21, 22 and 23, and Tables 8 and 9, while the desirability of the optimized flexural strength, color contour configurations and the response surface optimized configuration are presented in Figs. 24, 25, 26, 27 and 28.

8 $$ \begin{aligned} {\text{FS}} & = - {1}.{\text{95291E}} - {\text{16B}}^{{2}} + {\text{ C}}^{2} + {\text{FA}}^{2} + {\text{CA}}^{2} + {\text{w}}/{\text{c}}^{2} \, + {7}.{\text{26887E}} - 0{\text{8 w}}/{\text{c }}*{\text{ B}} \\ & \quad + {1}.0{5}0{\text{33E}} - 0{\text{9 CA }}*{\text{ B }} + {\text{ CA }}*{\text{ w}}/{\text{c }} + {3}.{\text{17661E}} - 0{\text{9 FA }}*{\text{ B}} \\ & \quad + {\text{ FA }}*{\text{ w}}/{\text{c }} + {\text{ FA }}*{\text{ CA }} + {9}.{44}0{\text{29E}} - 0{\text{9 C }}*{\text{ B }} + {\text{ C }}*{\text{ w}}/{\text{c }} \\ & \quad + \, 0.000{\text{513 C }}*{\text{ CA }} + \, 0.000{\text{372 C}}*{\text{FA }} - { 7}.{\text{17166E}} - 0{\text{6B}} \\ & \quad - { 26}.{5173}0{\text{w}}/{\text{c }} - \, 0.{24}0{\text{598CA }} - \, 0.{\text{193985FA }} - \, 0.{\text{912754C }} + { 453}.{62549} \\ \end{aligned} $$

**Table 6:** **FS** ANOVA for Quadratic model (Aliased).

Source	Sum of Squares	df	Mean Square	F-value	*p*-value	
Model	26.41	12	2.20	7.83	0.0022	Significant
A-C	14.99	1	14.99	53.38	< 0.0001	
B-FA	11.29	1	11.29	40.19	0.0001	
C-CA	7.59	1	7.59	27.01	0.0006	
D-w/c	3.69	1	3.69	13.13	0.0055	
E-B	0.0297	1	0.0297	0.1056	0.7527	
AB	16.01	1	16.01	57.00	< 0.0001	
AC	4.72	1	4.72	16.79	0.0027	
AD	0.0000	0				
AE	0.0304	1	0.0304	0.1082	0.7497	
BC	0.0000	0				
BD	0.0000	0				
BE	0.0279	1	0.0279	0.0994	0.7597	
CD	0.0000	0				
CE	0.0114	1	0.0114	0.0406	0.8448	
DE	0.0001	1	0.0001	0.0004	0.9840	
A^2^	0.0000	0				
B^2^	0.0000	0				
C^2^	0.0000	0				
D^2^	0.0000	0				
E^2^	0.0250	1	0.0250	0.0888	0.7724	
Residual	2.53	9	0.2809			
Lack of fit	1.35	4	0.3373	1.43	0.3468	Not significant
Pure error	1.18	5	0.2359			
Cor total	28.94	21

**Table 7:** Fit Statistics for the flexural strength model.

SD	0.5300	R^2^	0.9126
Mean	5.43	Adjusted R^2^	0.7961
C.V. %	9.77	Predicted R^2^	NA
		Adeq Precision	8.4396

**Table 8:** Constraints for the flexural strength model.

Name	Goal	Lower Limit	Upper Limit	Lower Weight	Upper Weight	Importance
A:C	Is in range	350	456	1	1	3
B:FA	Is in range	555	861	1	1	3
C:CA	Is in range	900	1227.5	1	1	3
D:w/c	Is in range	0.4	0.5	1	1	3
E:B	Is in range	1000	1E+09	1	1	3
CS	Maximize	18.67	61.79	1	1	3
StdErr (CS)	None	1.17346	2.29627	1	1	3
STS	Maximize	1.8	3.2	1	1	3
StdErr (STS)	None	0.111229	0.217656	1	1	3
FS	Maximize	4.12	8.6	1	1	3
StdErr (FS)	None	0.270859	0.530025	1	1	3

**Table 9:** Selected optimized solution from the 100 solutions found.

Number	C	FA	CA	w/c	B	CS	StdErr (CS)	STS	StdErr (STS)	FS	StdErr (FS)	Desirability	
1	420.548	819.613	905.112	0.413	710,039,868.320	4353.398	2491.575	172.706	236.168	179.097	575.106	1.000	Selected

### Response surface methodology analysis for the slump

**Figure 29:**
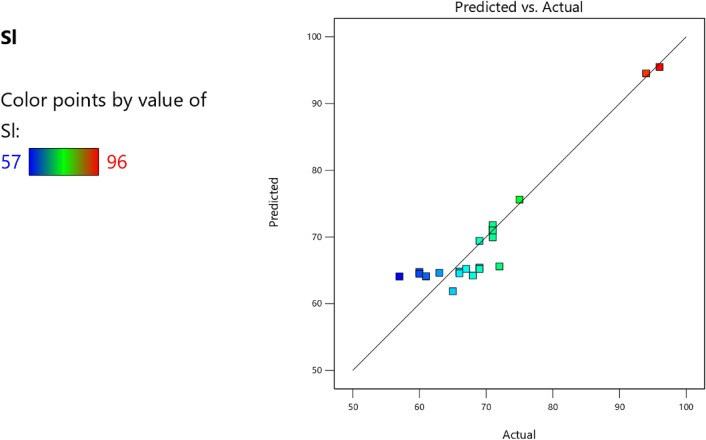
Slump model predicted versus actual values scatter plot.

**Figure 30:**
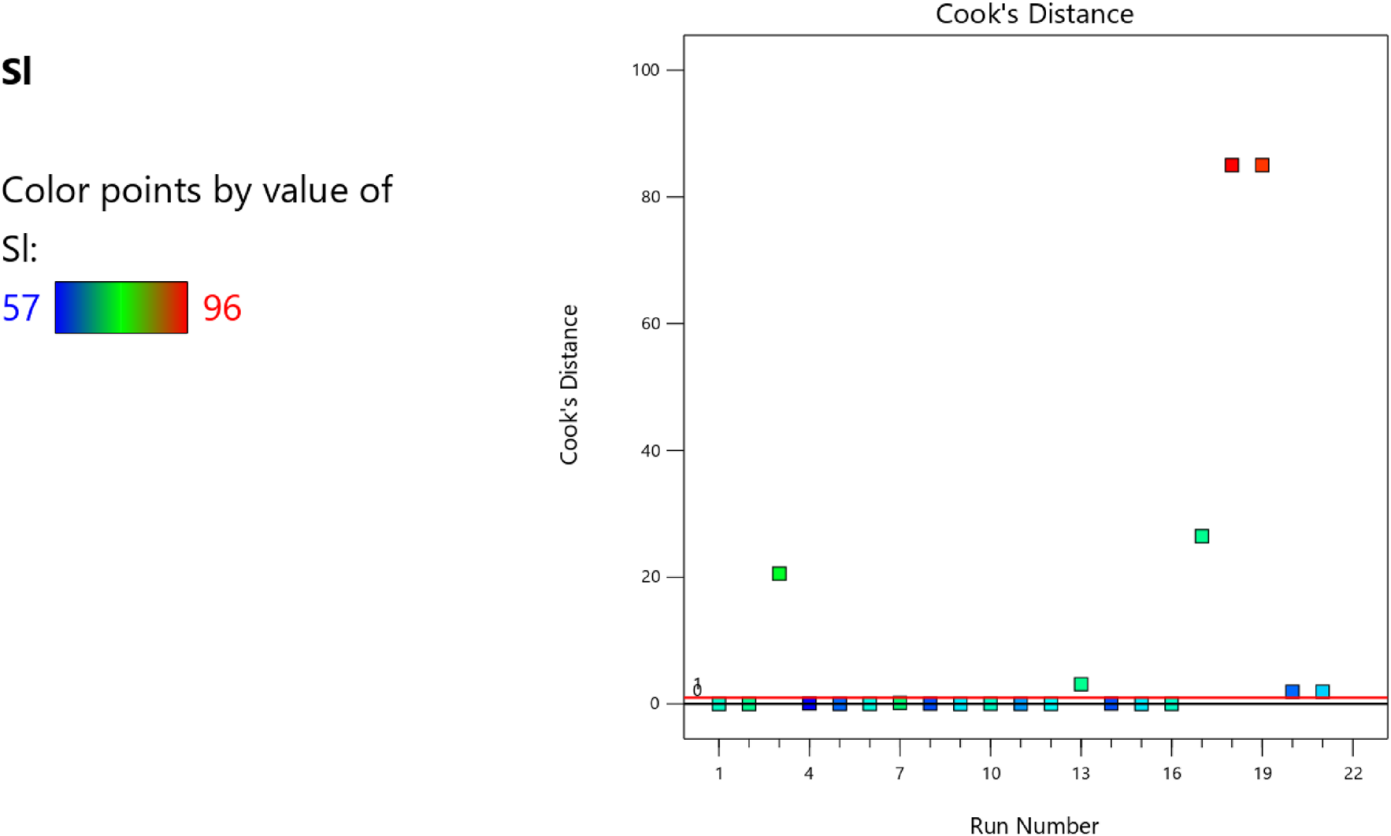
Slump model cook’s distance scatter plot.

**Figure 31:**
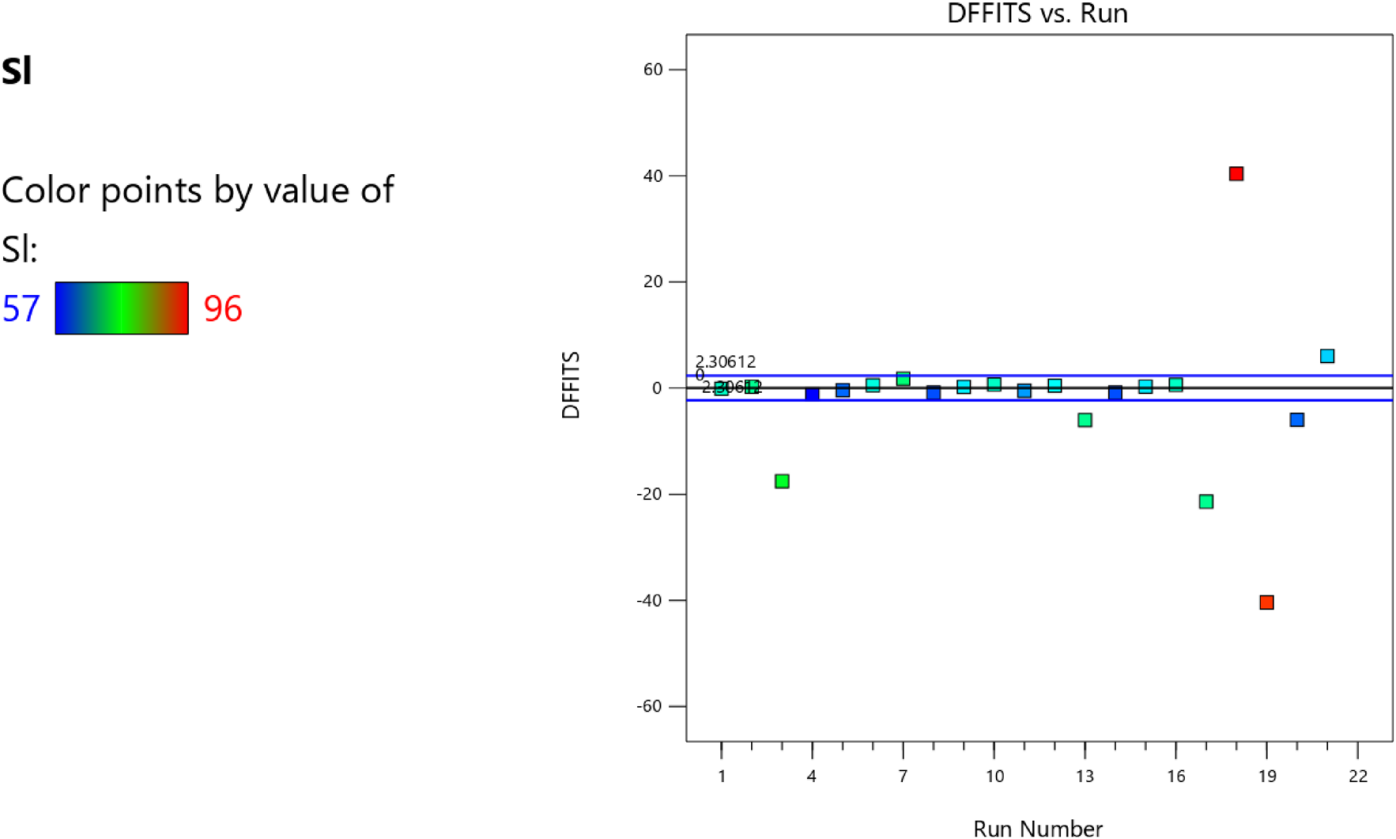
Slump model DFFITS versus run values scatter plot.

**Figure 32:**
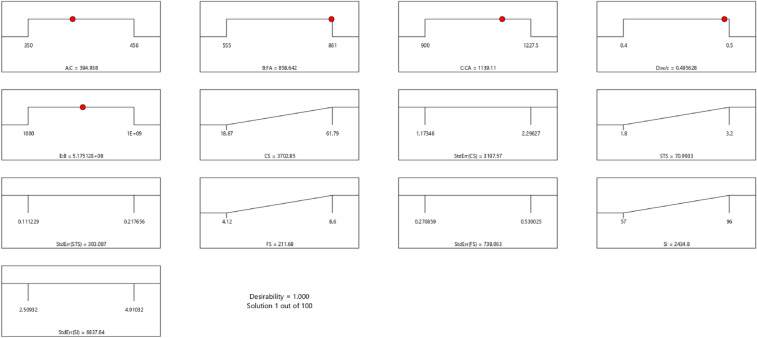
Slump model desirability optimized plot.

**Figure 33:**
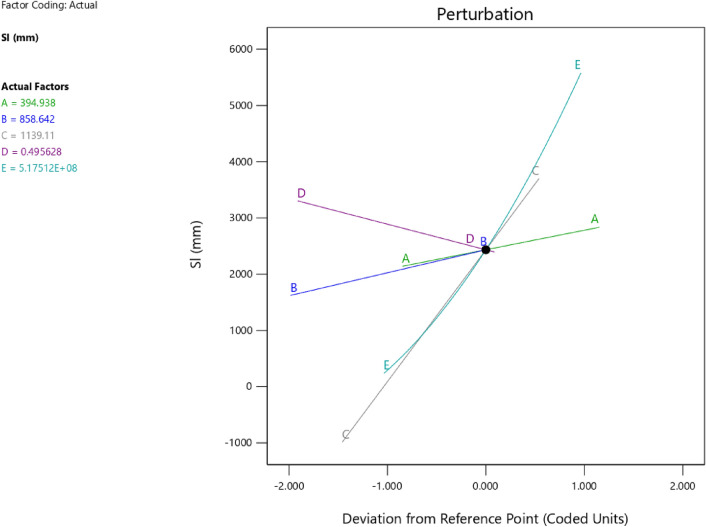
Slump model perturbation optimized plot.

**Figure 34:**
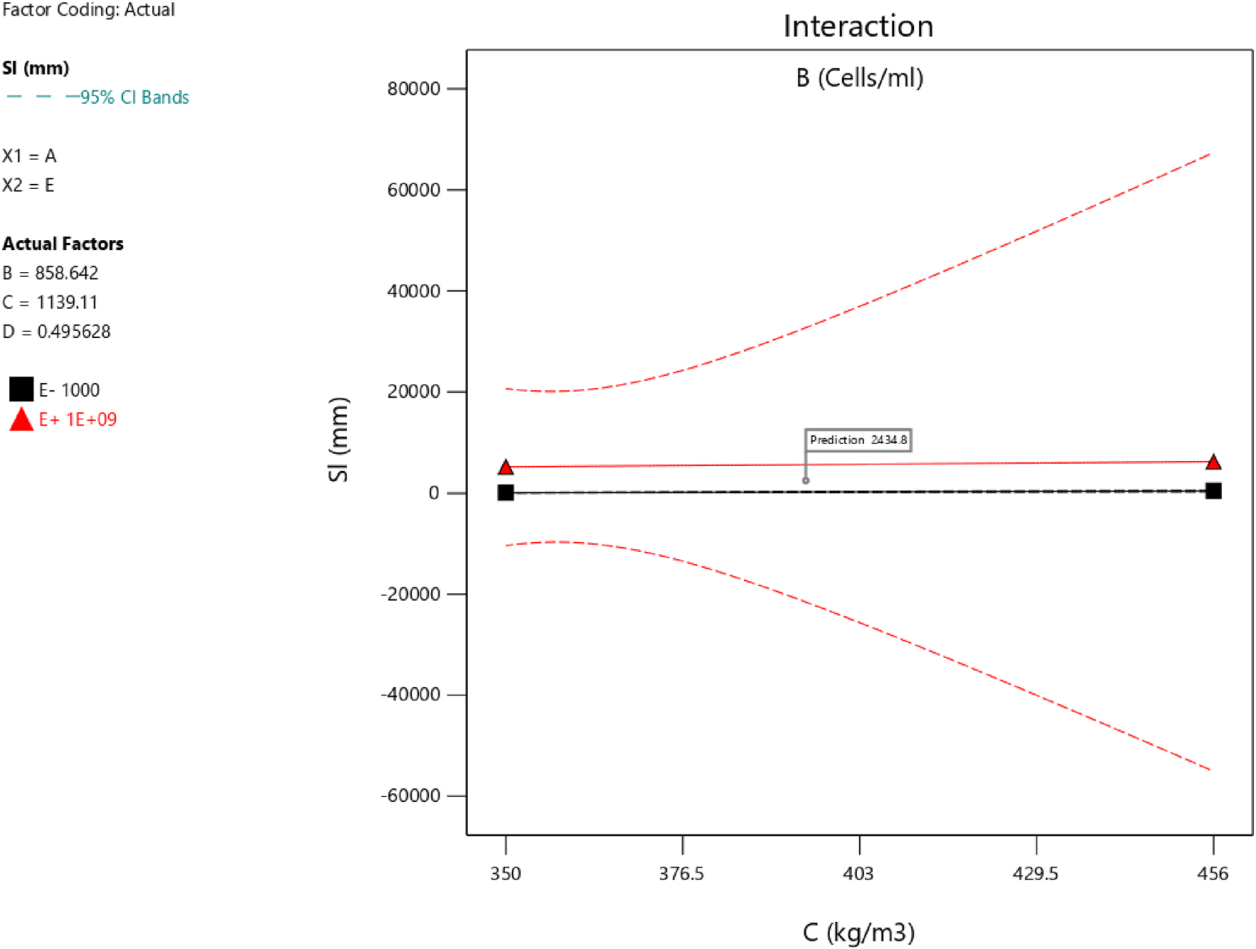
Slump model interaction optimized plot.

**Figure 35:**
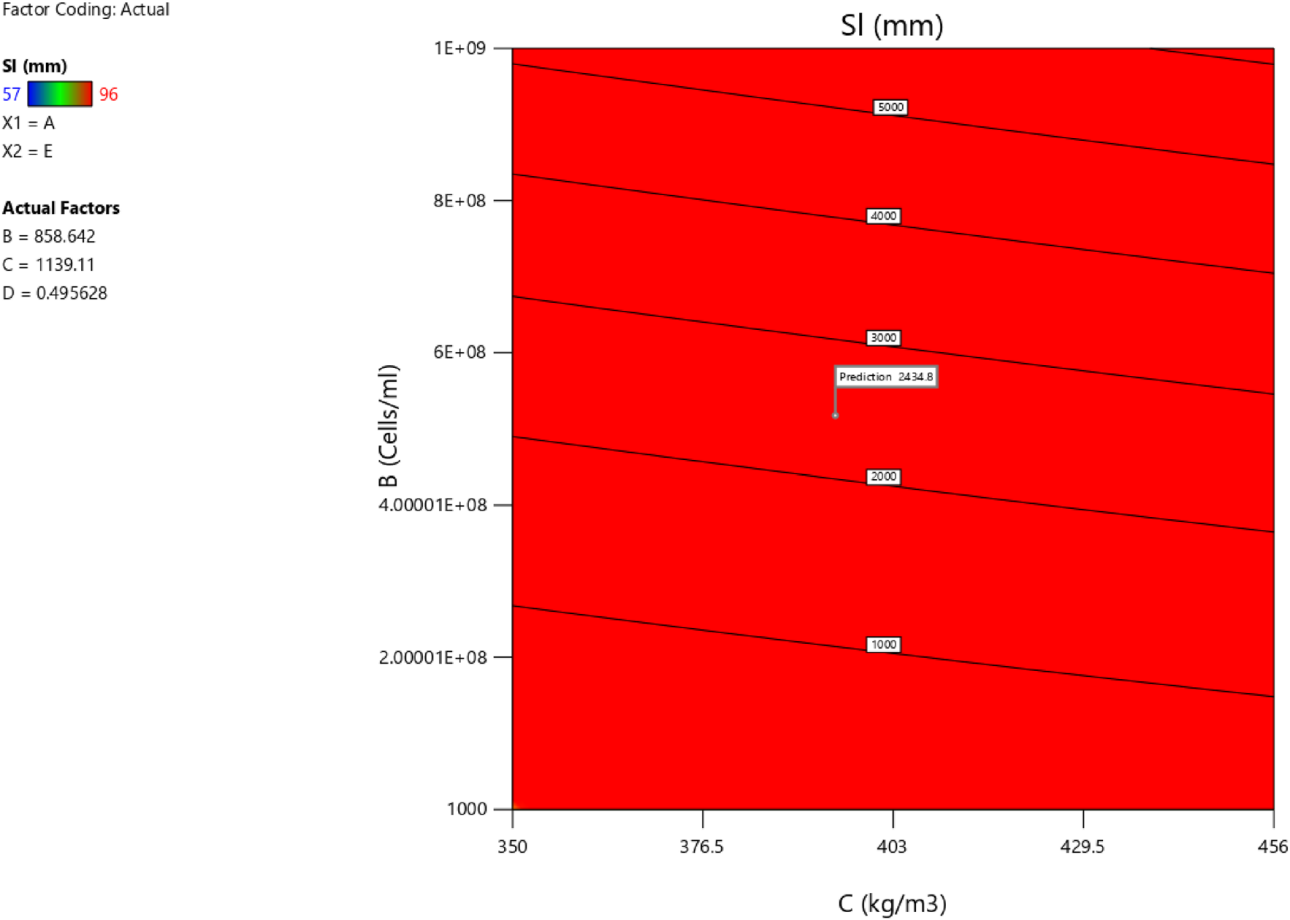
Slump model contour optimized plot.

**Figure 36:**
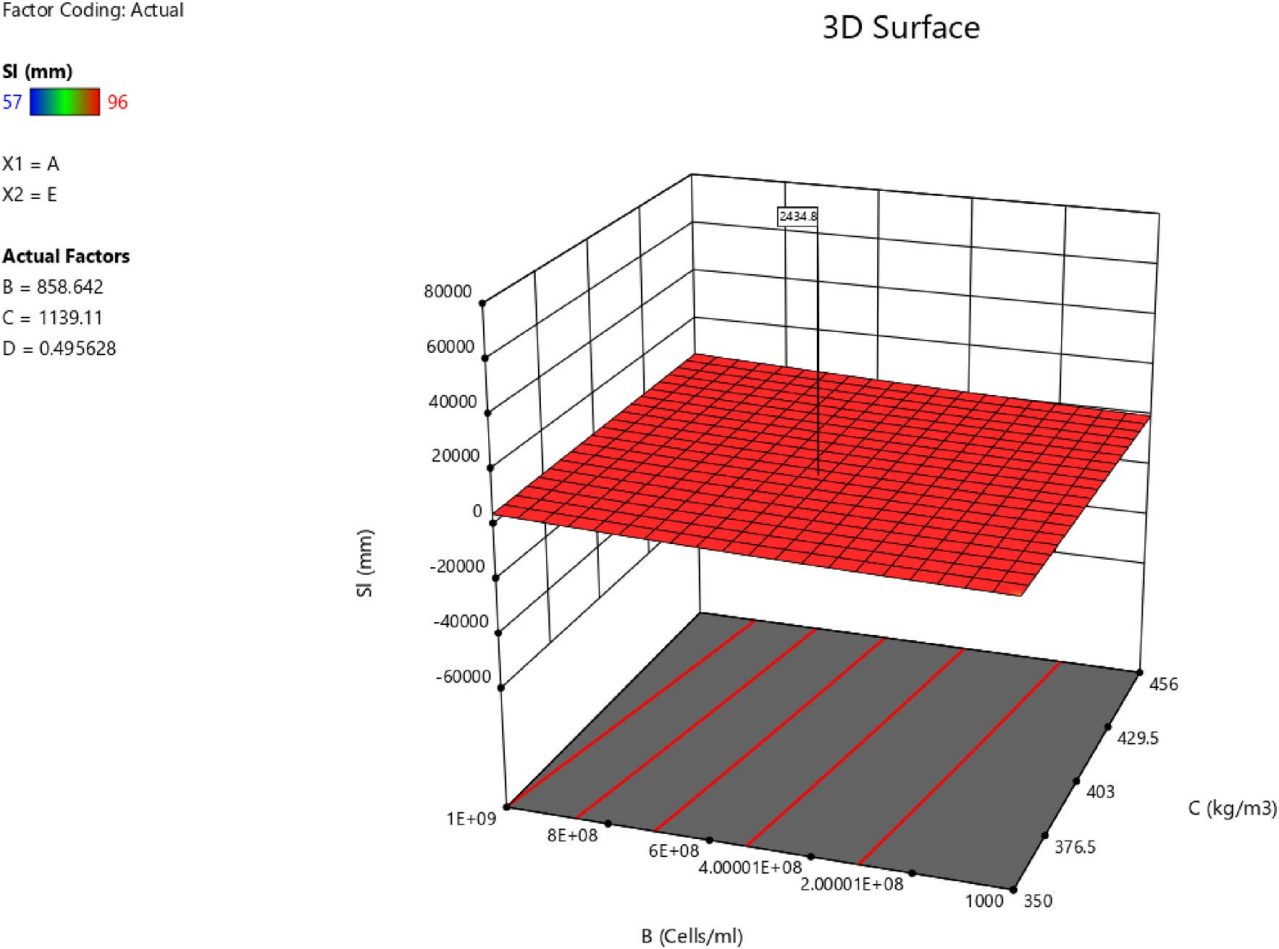
Slump model 3D surface configuration optimized plot.

Factor coding is actual. Sum of squares is Type III—Partial. The Model F-value of 5.90 implies the model is significant. There is only a 0.61% chance that an F-value this large could occur due to noise. *P*-values less than 0.0500 indicate model terms are significant. In this case B, C, D, AC are significant model terms. Values greater than 0.1000 indicate the model terms are not significant. If there are many insignificant model terms (not counting those required to support hierarchy), model reduction may improve your model. The Lack of Fit F-value of 1.31 implies the Lack of Fit is not significant relative to the pure error. There is a 38.00% chance that a Lack of Fit F-value this large could occur due to noise. Non-significant lack of fit is good—we want the model to fit. Adeq Precision measures the signal to noise ratio. A ratio greater than 4 is desirable. Your ratio of 8.902 indicates an adequate signal. This model can be used to navigate the design space. The coefficient estimate represents the expected change in response per unit change in factor value when all remaining factors are held constant. The intercept in an orthogonal design is the overall average response of all the runs. The coefficients are adjustments around that average based on the factor settings. When the factors are orthogonal the VIFs are 1; VIFs greater than 1 indicate multi-colinearity, the higher the VIF the more severe the correlation of factors. As a rough rule, VIFs less than 10 are tolerable. The equation in terms of actual factors can be used to make predictions about the response for given levels of each factor. Here, the levels should be specified in the original units for each factor. This equation should not be used to determine the relative impact of each factor because the coefficients are scaled to accommodate the units of each factor and the intercept is not at the center of the design space. The above analyses are presented in Tables 10 and 11, Figs. 29, 30, 31 and 32, and Tables 12 and 13, while the desirability of the optimized flexural strength, color contour configurations and the response surface optimized configuration are presented in Figs. 33, 34, 35 and 36.

9 $$ \begin{aligned} {\text{Sl}} & = {2}.{\text{27139E}} - {\text{15B}}^{{2}} {\text{C}}^{2} \, + {\text{ FA}}^{2} \, + {\text{ CA}}^{2} \, + {\text{ w}}/{\text{c}}^{2} \, - 0.0000{\text{18w}}/{\text{c }}*{\text{ B}} \\ & \quad + { 2}.{695}0{\text{8E}} - 0{\text{8CA}}*{\text{B }} + {\text{ CA }}*{\text{ w}}/{\text{c }} + {3}.{\text{33628E}} - 0{\text{9FA}}*{\text{B}} \\ & \quad + {\text{ FA}}*{\text{CA }} + {\text{ FA}}*{\text{w}}/{\text{c }} + { 6}.{\text{34117E}} - 0{\text{9C}}*{\text{B }} + {\text{ C}}*{\text{w}}/{\text{c}} \\ & \quad + \, 0.00{52}0{\text{7C}}*{\text{CA }} - 0.000{\text{114C}}*{\text{FA }} - \, 0.0000{\text{24B }} + { 3}0{3}.{\text{68534w}}/{\text{c}} \\ & \quad - { 1}.{6836}0{\text{CA }} + \, 0.{\text{992273FA }} - { 2}.{\text{56849C }} - { 136}.{53734} \\ \end{aligned} $$

**Table 10:** **Sl** ANOVA for Quadratic model (Aliased).

Source	Sum of Squares	df	Mean Square	F-value	*p*-value	
Model	1706.27	12	142.19	5.90	0.0061	Significant
A-C	118.74	1	118.74	4.92	0.0536	
B-FA	295.45	1	295.45	12.25	0.0067	
C-CA	371.50	1	371.50	15.41	0.0035	
D-w/c	483.74	1	483.74	20.06	0.0015	
E-B	0.3321	1	0.3321	0.0138	0.9092	
AB	1.49	1	1.49	0.0619	0.8091	
AC	485.37	1	485.37	20.13	0.0015	
AD	0.0000	0				
AE	0.0137	1	0.0137	0.0006	0.9815	
BC	0.0000	0				
BD	0.0000	0				
BE	0.0308	1	0.0308	0.0013	0.9723	
CD	0.0000	0				
CE	7.51	1	7.51	0.3116	0.5903	
DE	7.41	1	7.41	0.3074	0.5928	
A^2^	0.0000	0				
B^2^	0.0000	0				
C^2^	0.0000	0				
D^2^	0.0000	0				
E^2^	3.38	1	3.38	0.1400	0.7169	
Residual	217.00	9	24.11			
Lack of fit	111.00	4	27.75	1.31	0.3800	Not significant
Pure error	106.00	5	21.20			
Cor total	1923.27	21

**Table 11:** Fit statistics for the concrete slump (Sl).

SD	4.91	R^2^	0.8872
Mean	69.18	Adjusted R^2^	0.7367
C.V. %	7.10	Predicted R^2^	NA
		Adeq Precision	8.9021

**Table 12:** Selected optimized slump model from 100 Solutions found.

Number	C	FA	CA	w/c	B	CS	StdErr(CS)	STS	StdErr(STS)	FS	StdErr(FS)	Sl	StdErr(Sl)	Desirability	
1	394.938	858.642	1139.106	0.496	517,511,759.806	3702.854	3197.565	70.993	303.087	211.680	738.063	2434.802	6837.642	1.000	Selected

**Table 13:** Constraints for the slump model.

Name	Goal	Lower Limit	Upper Limit	Lower Weight	Upper Weight	Importance
A:C	Is in range	350	456	1	1	3
B:FA	Is in range	555	861	1	1	3
C:CA	Is in range	900	1227.5	1	1	3
D:w/c	Is in range	0.4	0.5	1	1	3
E:B	Is in range	1000	1E+09	1	1	3
CS	Maximize	18.67	61.79	1	1	3
StdErr(CS)	None	1.17346	2.29627	1	1	3
STS	Maximize	1.8	3.2	1	1	3
StdErr(STS)	None	0.111229	0.217656	1	1	3
FS	Maximize	4.12	8.6	1	1	3
StdErr(FS)	None	0.270859	0.530025	1	1	3
Sl	Maximize	57	96	1	1	3
StdErr(Sl)	None	2.50932	4.91032	1	1	3

### Response surface methodology analysis for the splitting tensile strength

**Figure 37:**
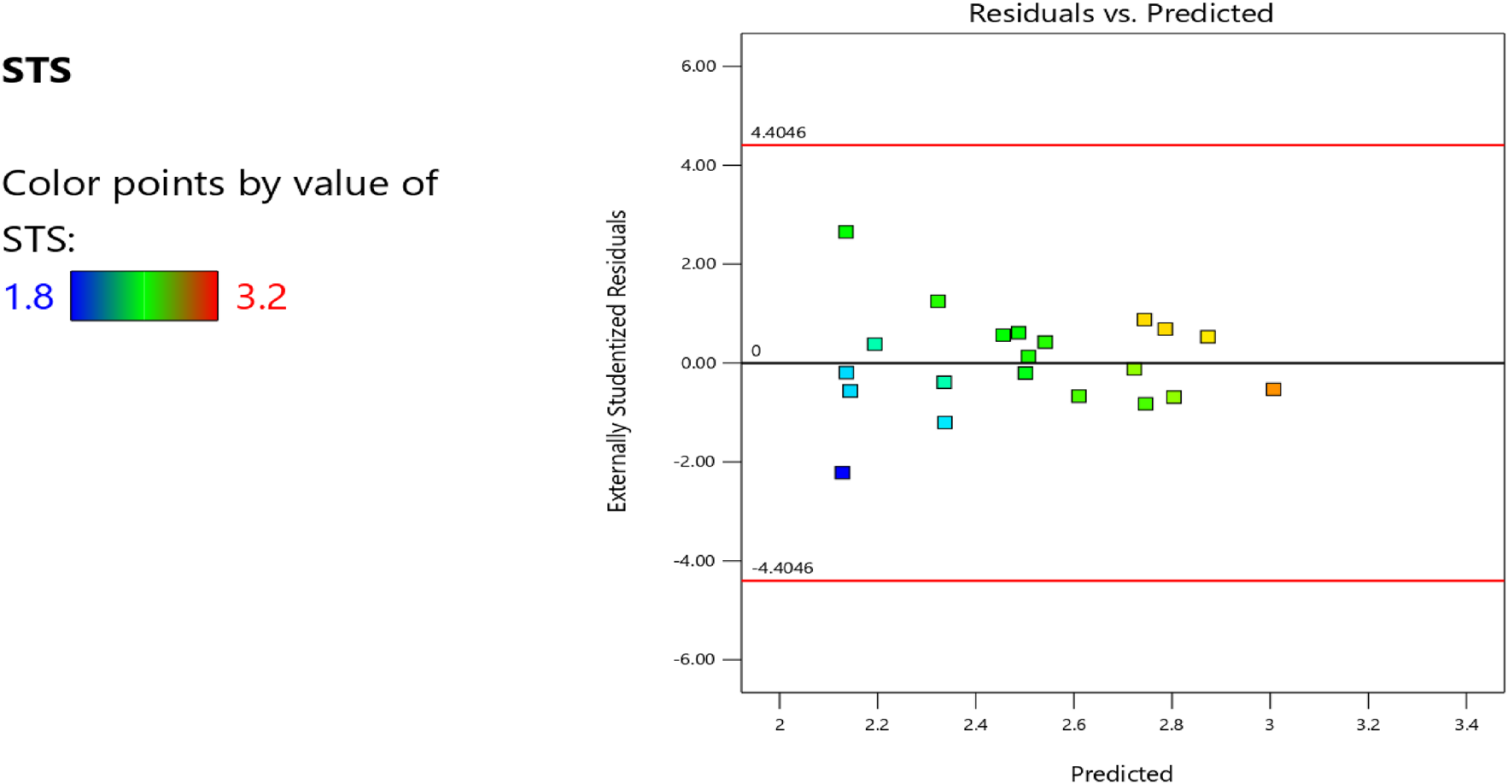
Splitting tensile strength model residual versus predicted optimized plot.

**Figure 38:**
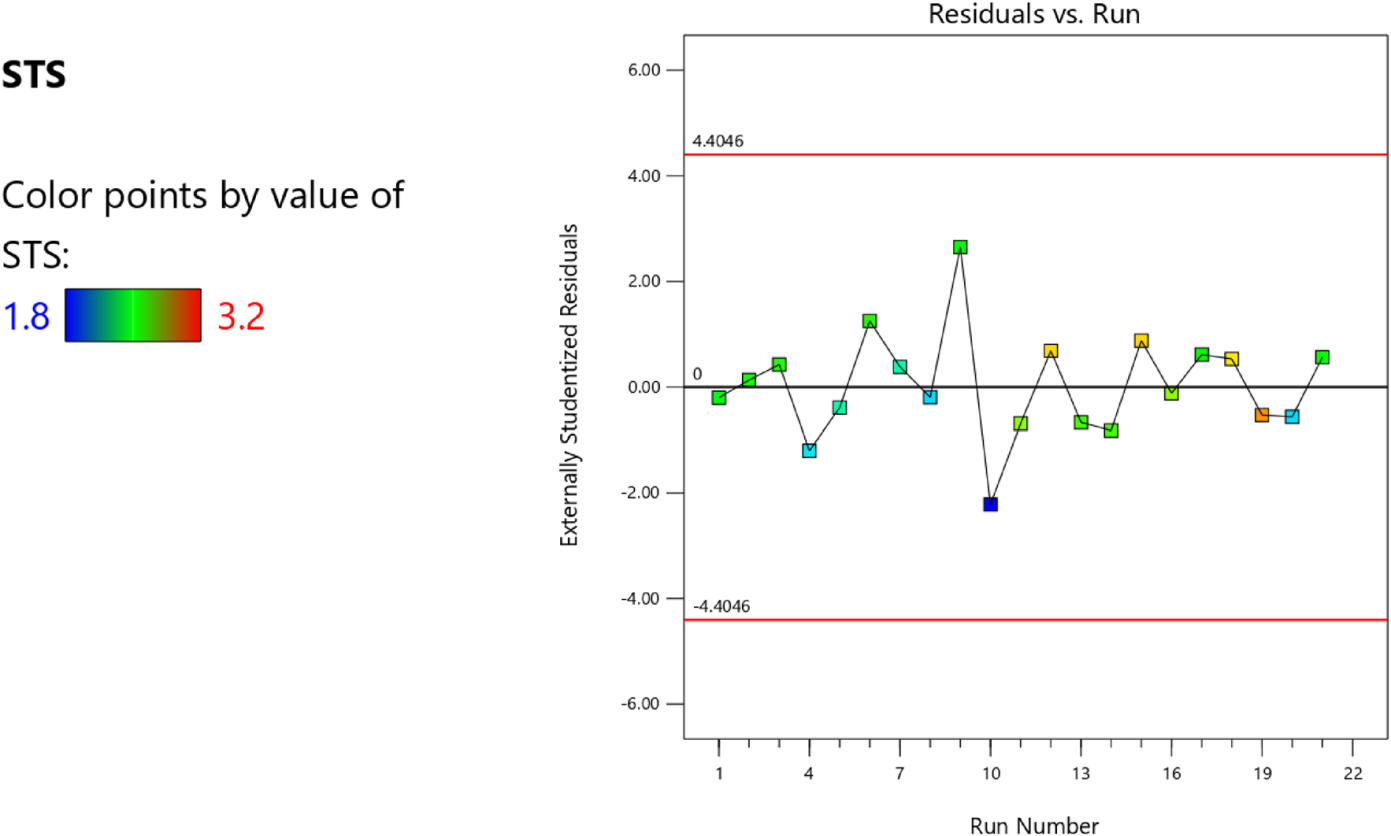
Splitting tensile strength model residual versus run optimized plot.

**Figure 39:**
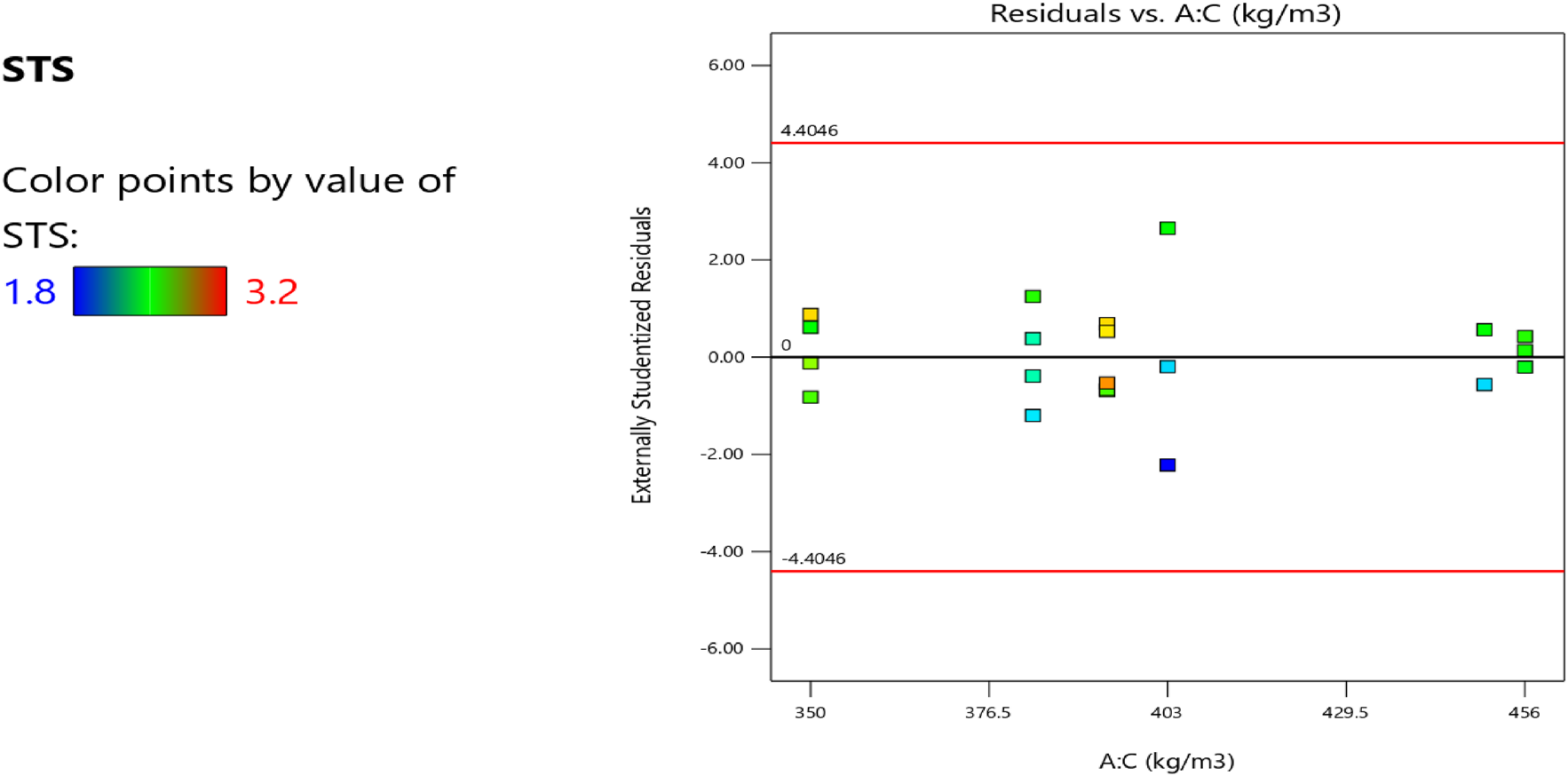
Splitting tensile strength model residual versus cement optimized plot.

**Figure 40:**
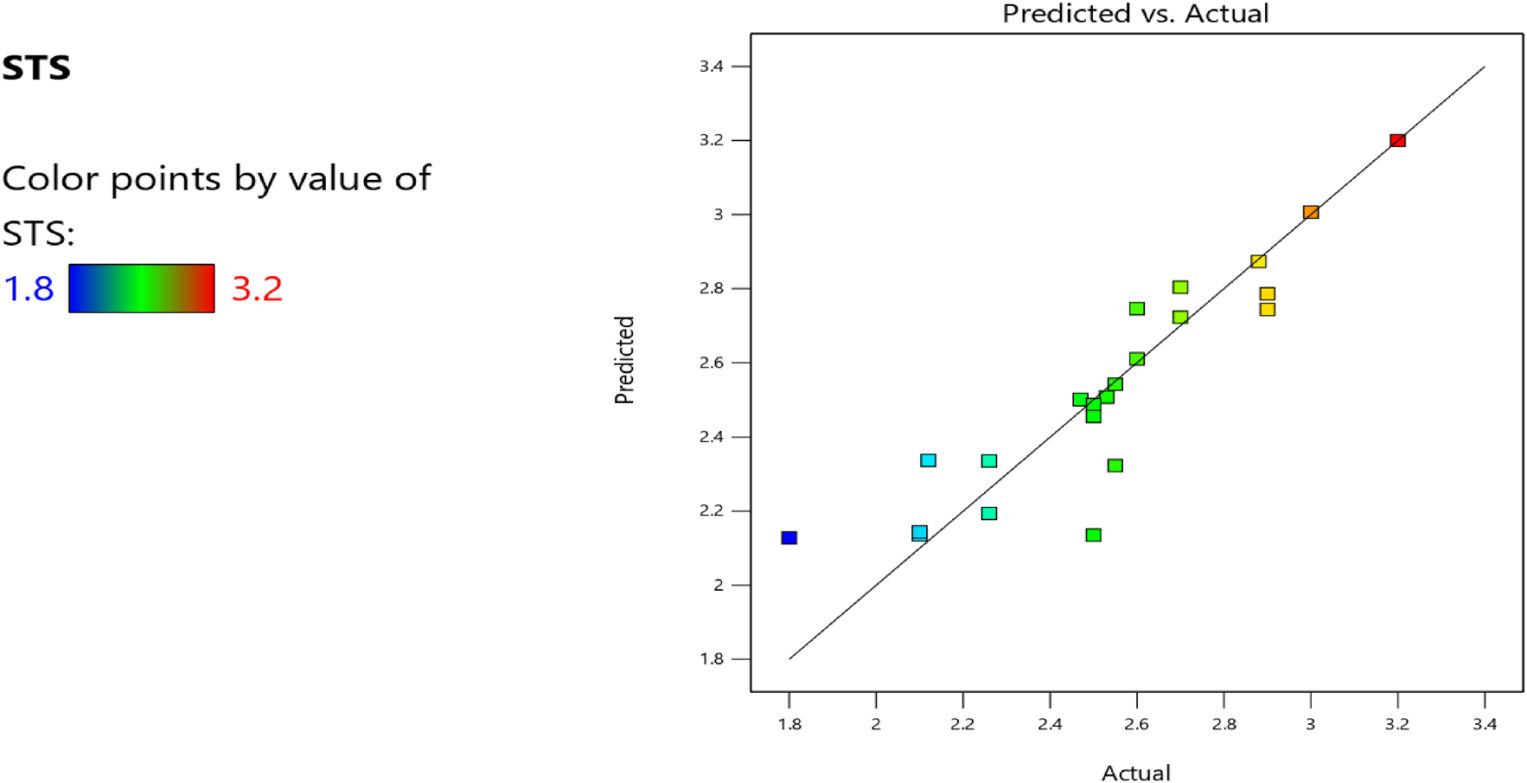
Splitting tensile strength model actual versus predicted optimized scatter plot.

**Figure 41:**
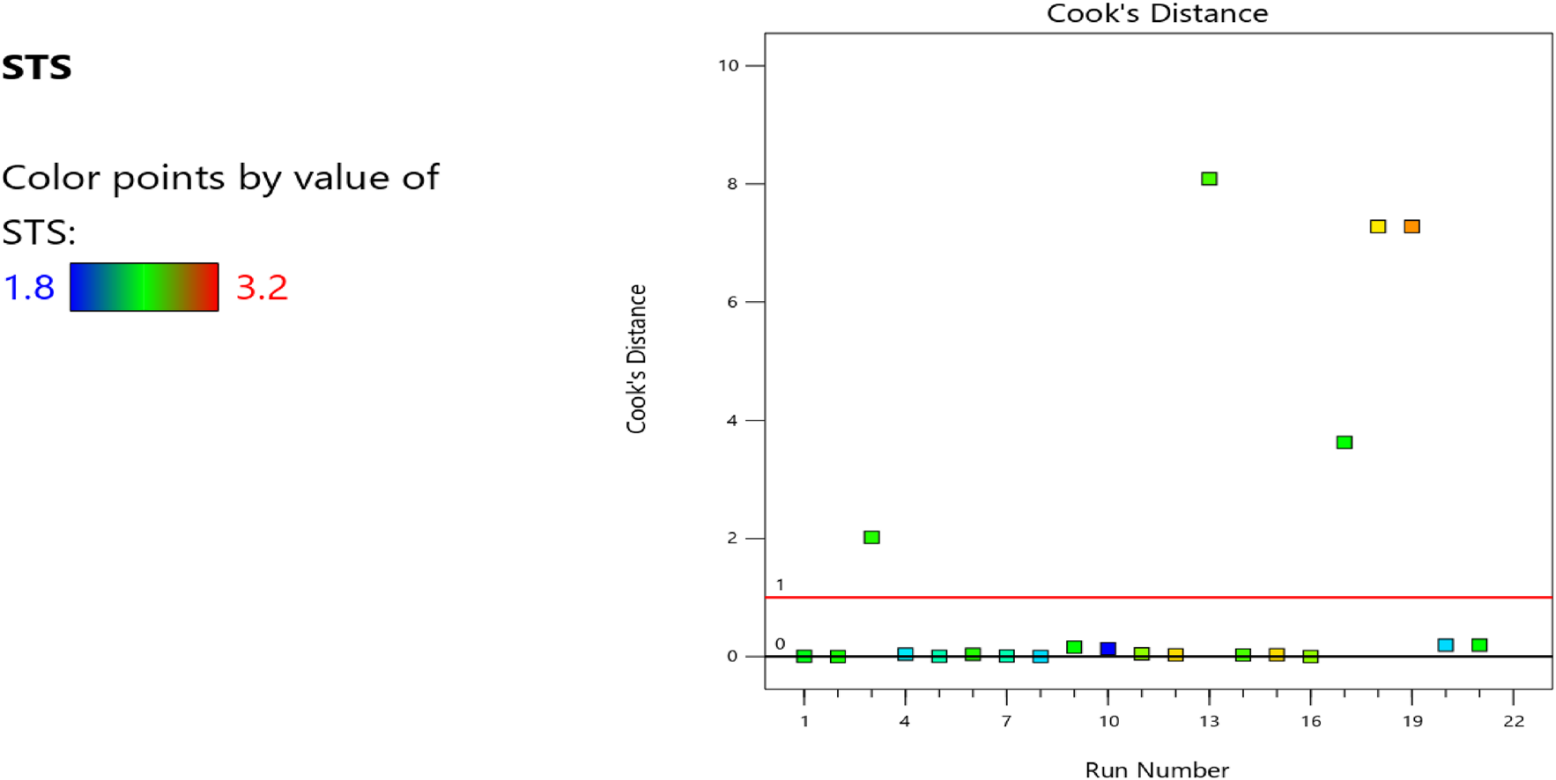
Splitting tensile strength model cook’s distance optimized scatter plot.

**Figure 42:**
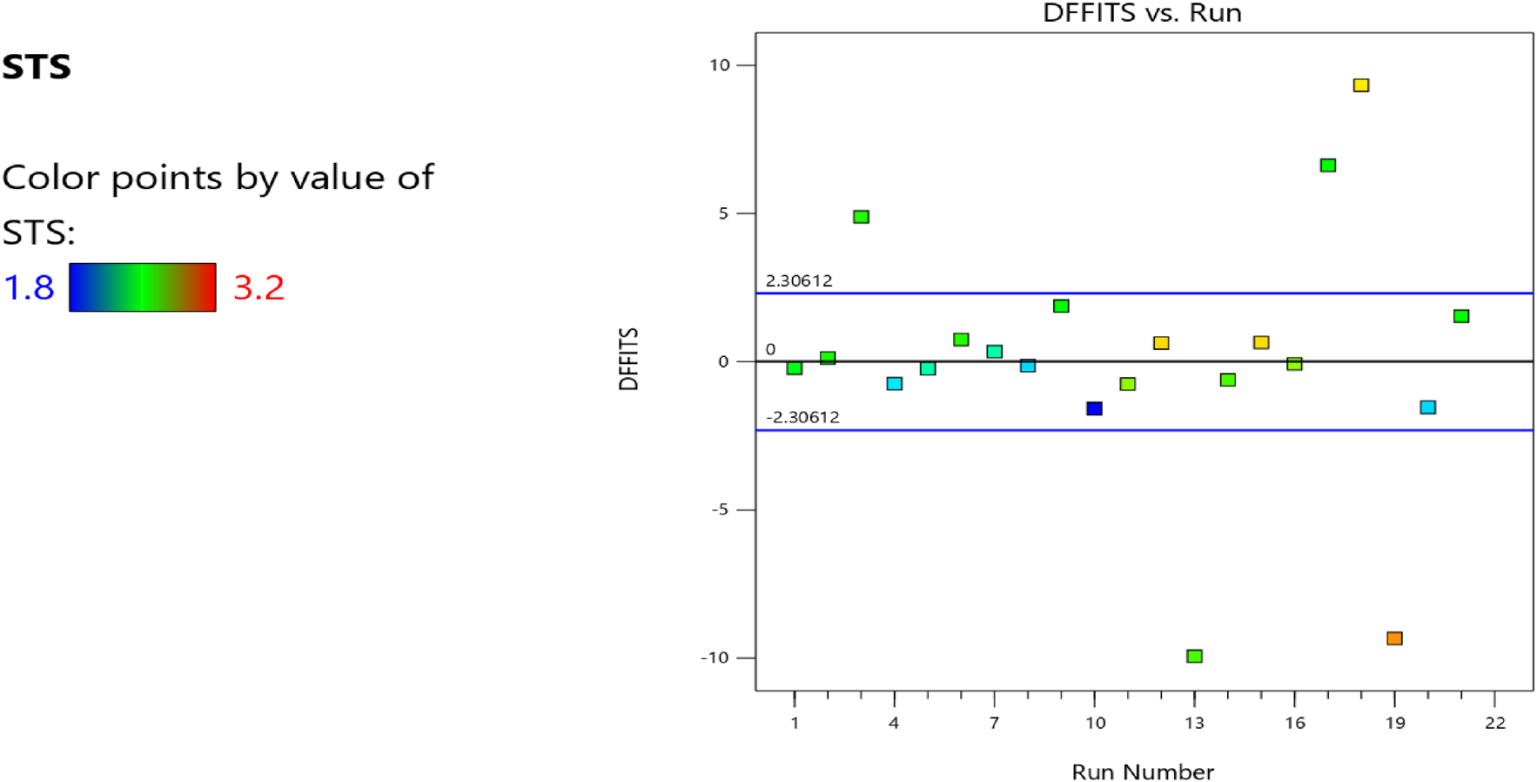
Splitting tensile strength model DFFITS versus run optimized scatter plot.

**Figure 43:**
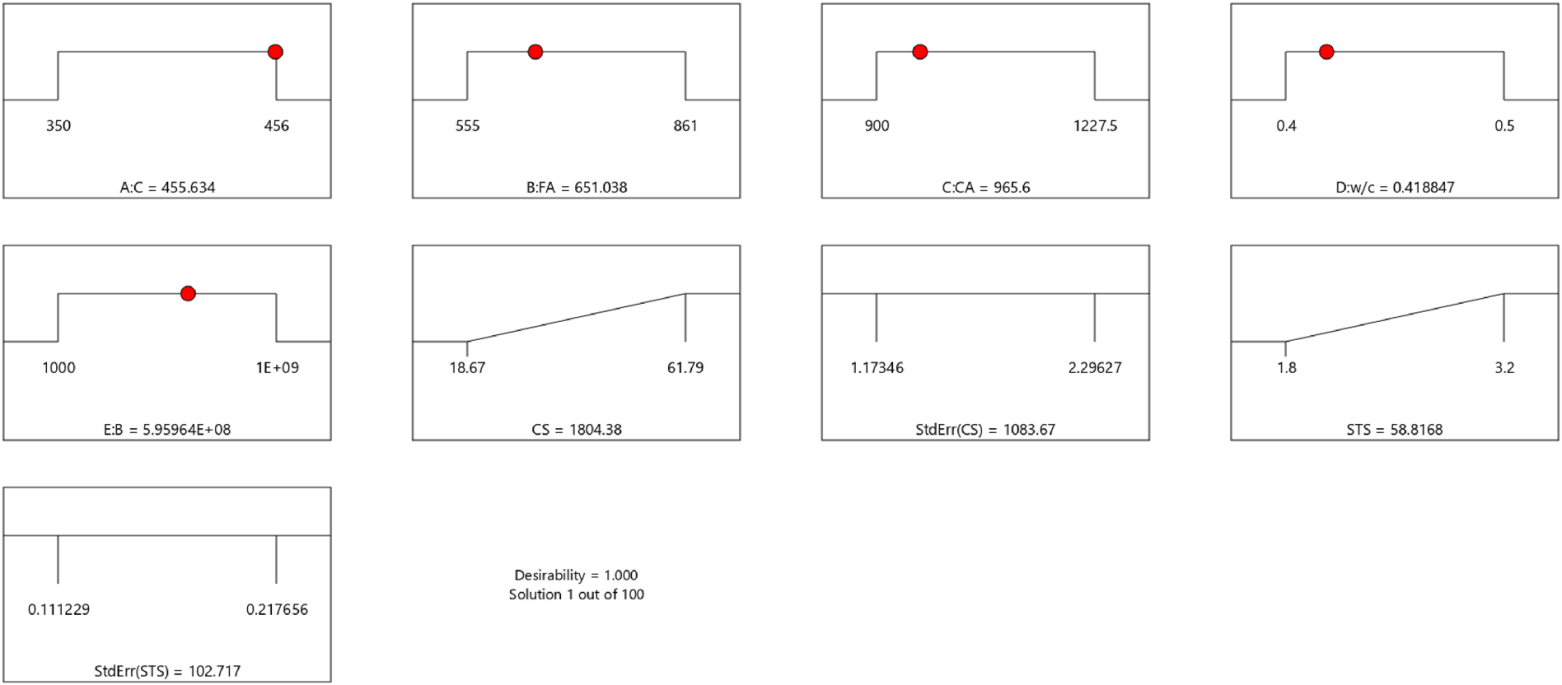
Splitting tensile strength model desirability optimized plot.

**Figure 44:**
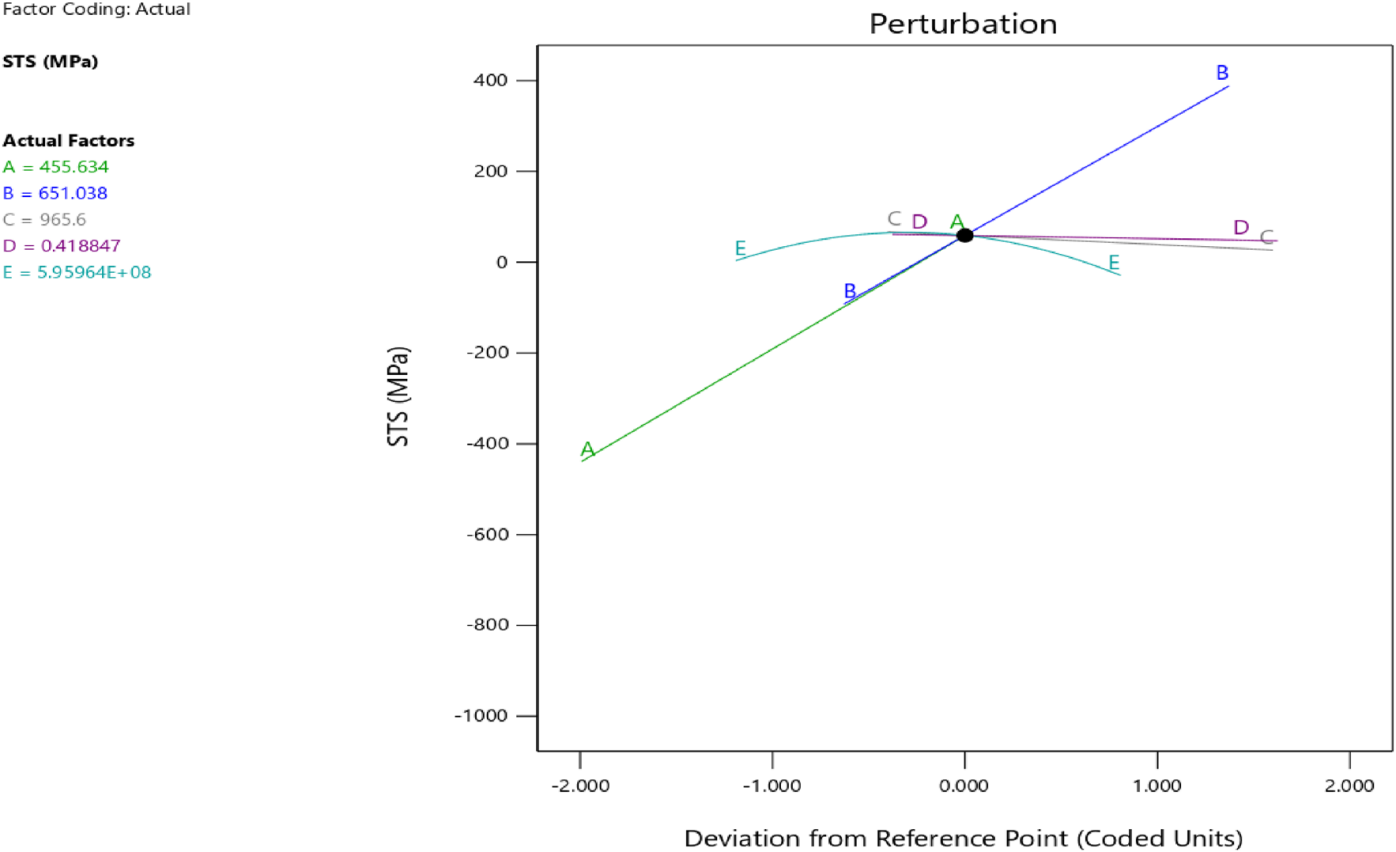
Splitting tensile strength model perturbation optimized plot.

**Figure 45:**
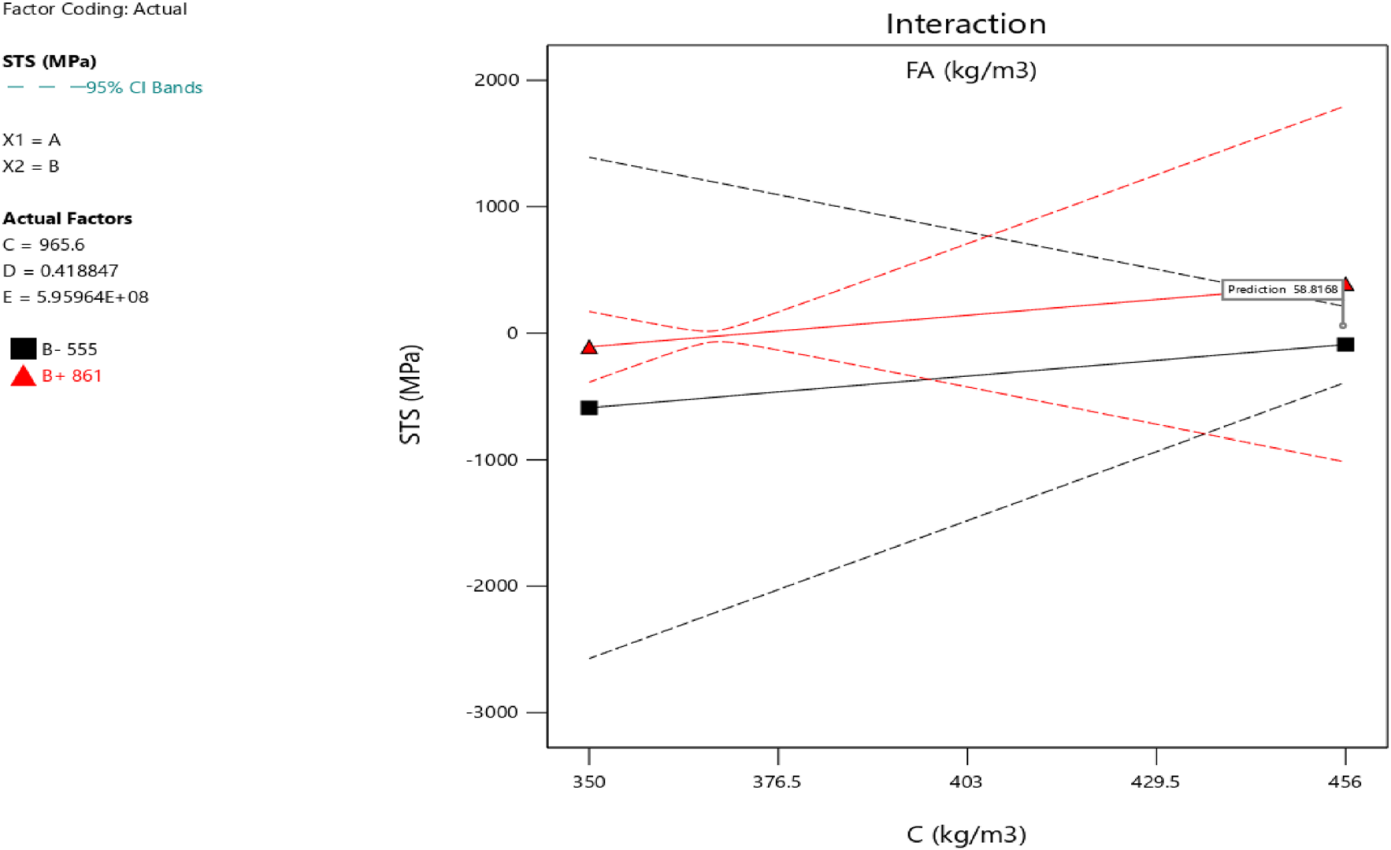
Splitting tensile strength model interaction optimized plot.

**Figure 46:**
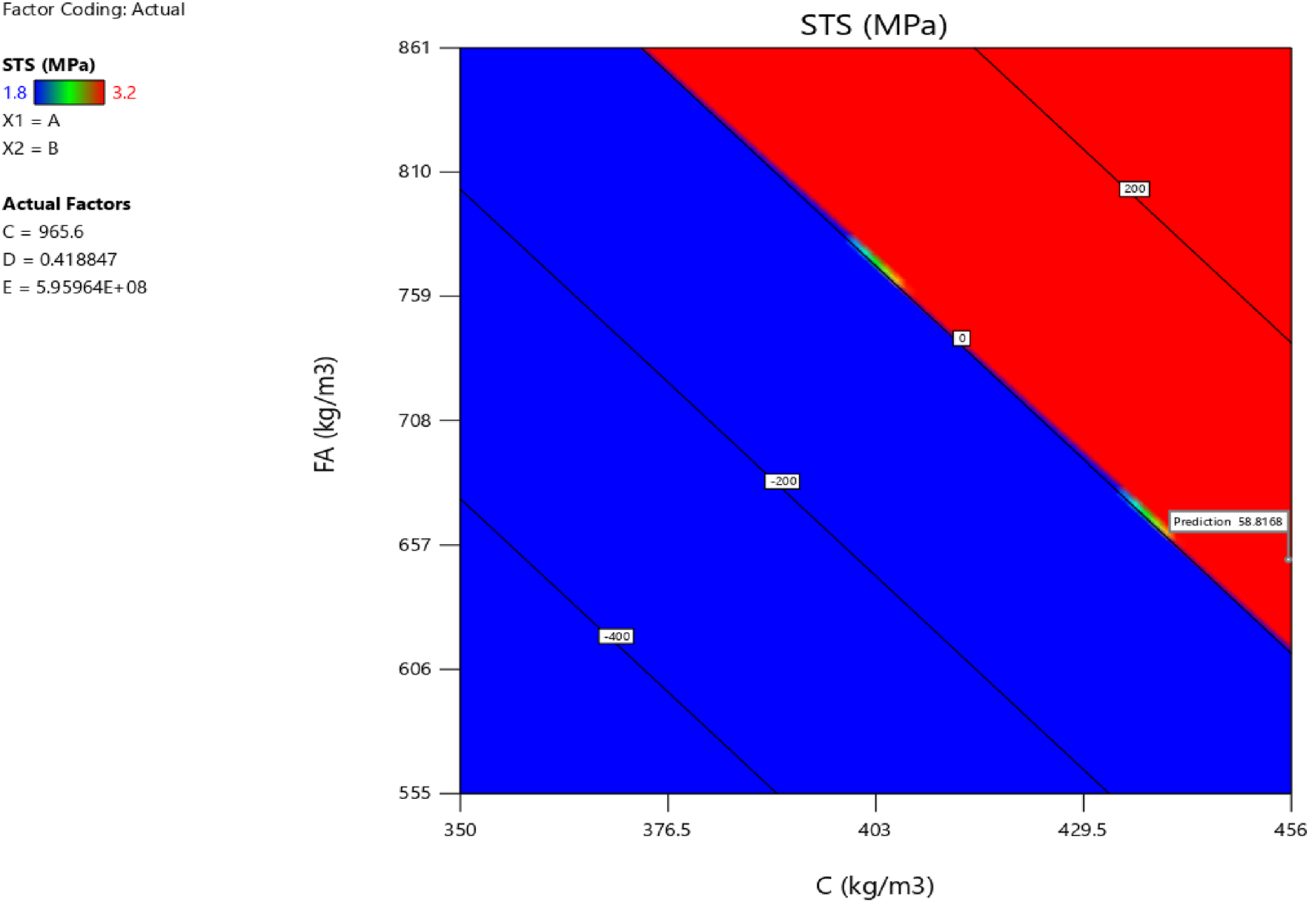
Splitting tensile strength model contour optimized plot.

**Figure 47:**
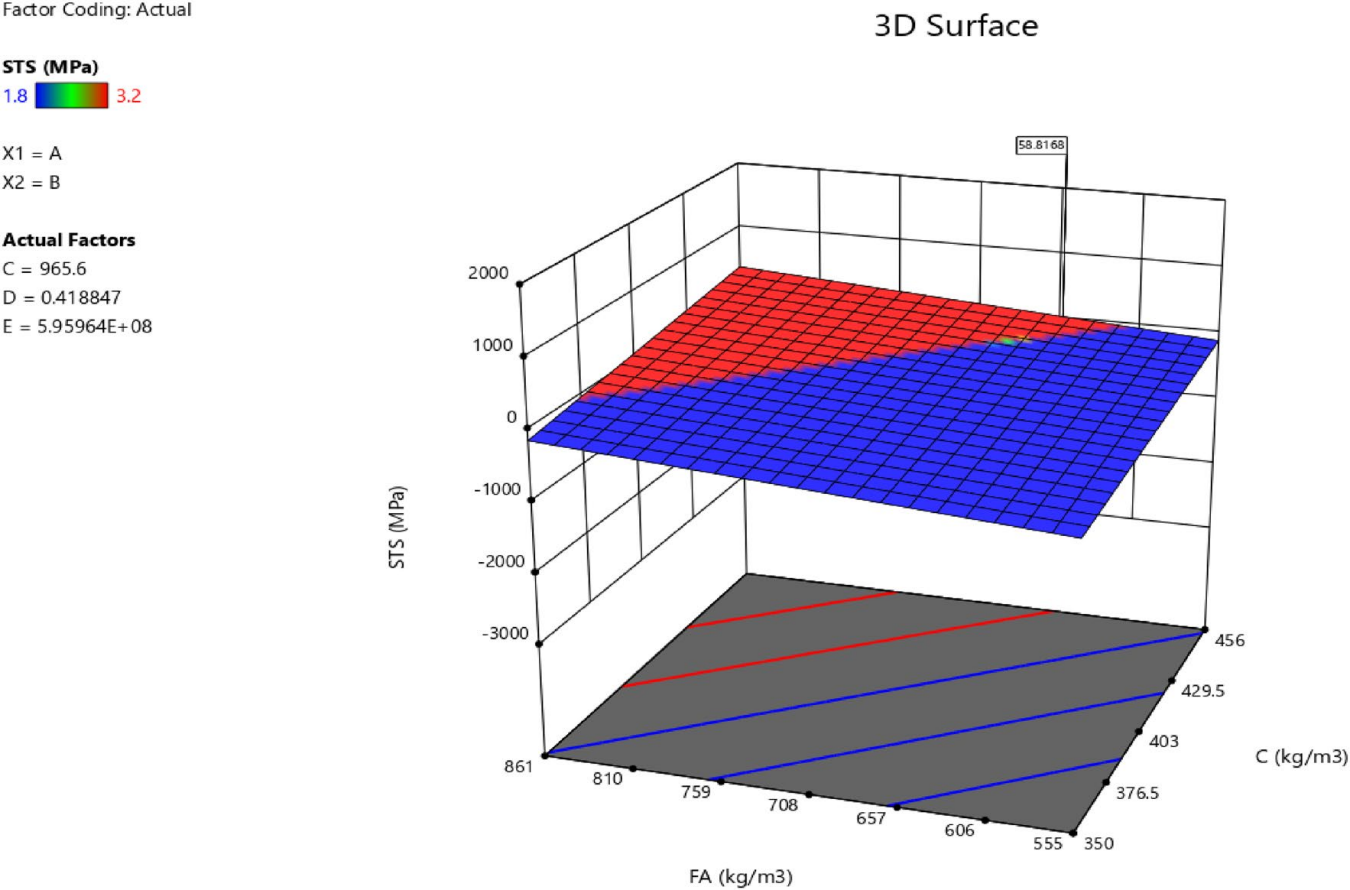
Splitting tensile strength model 3D optimized plot.

Factor coding is actual. Sum of squares is Type III—Partial. The Model F-value of 3.40 implies the model is significant. There is only a 3.73% chance that an F-value this large could occur due to noise. *P*-values less than 0.0500 indicate model terms are significant. In this case B, AB are significant model terms. Values greater than 0.1000 indicate the model terms are not significant. If there are many insignificant model terms (not counting those required to support hierarchy), model reduction may improve your model. The Lack of Fit F-value of 0.96 implies the Lack of Fit is not significant relative to the pure error. There is a 50.20% chance that a Lack of Fit F-value this large could occur due to noise. Non-significant lack of fit is good—we want the model to fit. Adeq Precision measures the signal to noise ratio. A ratio greater than 4 is desirable. Your ratio of 6.406 indicates an adequate signal. This model can be used to navigate the design space. The coefficient estimate represents the expected change in response per unit change in factor value when all remaining factors are held constant. The intercept in an orthogonal design is the overall average response of all the runs. The coefficients are adjustments around that average based on the factor settings. When the factors are orthogonal the VIFs are 1; VIFs greater than 1 indicate multi-colinearity, the higher the VIF the more severe the correlation of factors. As a rough rule, VIFs less than 10 are tolerable. The equation in terms of actual factors can be used to make predictions about the response for given levels of each factor. Here, the levels should be specified in the original units for each factor. This equation should not be used to determine the relative impact of each factor because the coefficients are scaled to accommodate the units of each factor and the intercept is not at the center of the design space. The above analyses are presented in Tables 14 and 15, Figs. 37, 38, 39, 40, 41 and 42, and Tables 16 and 17, while the desirability of the optimized flexural strength, color contour configurations and the response surface optimized configuration are presented in Figs. 43, 44, 45, 46 and 47.

10 $$ \begin{aligned} {\text{STS}} & = - {3}.{1142}0{\text{E}} - {\text{16B}}^{2} \, + {\text{ C}}^{2} \, + {\text{ FA}}^{2} \, + {\text{ CA}}^{2} \, + {\text{ w}}/{\text{c}}^{2} \, - {2}.{5}0{18}0{\text{E}} - 0{\text{7w}}/{\text{c}}*{\text{B}} \\ & \quad - {2}.{\text{33168E}} - {1}0{\text{CA}}*{\text{B }} + {\text{ CA}}*{\text{w}}/{\text{c }} + {2}.{\text{59949E}} - 0{\text{9FA}}*{\text{B}} \\ & \quad + {\text{ FA}}*{\text{CA }} + {\text{ FA}}*{\text{w}}/{\text{c }} + {7}.{8}0{\text{711E}} - 0{\text{9C}}*{\text{B }} + {\text{ C}}*{\text{w}}/{\text{c}} \\ & \quad + 0.0000{\text{74C}}*{\text{CA }} - \, 0.0000{\text{58C }}*{\text{ FA }} - { 4}.{64}0{\text{45E}} - 0{\text{6B}} \\ & \quad + {5}.{382}0{\text{1w}}/{\text{c }} - 0.0{\text{16963CA }} + \, 0.0{47}00{\text{1FA }} + 0.0{\text{29667C }} - { 42}.{16944} \\ \end{aligned} $$

**Table 14:** **STS** ANOVA for Quadratic model (Aliased).

Source	Sum of Squares	df	Mean Square	F-value	*p*-value	
Model	1.93	12	0.1609	3.40	0.0373	Significant
A-C	0.0158	1	0.0158	0.3344	0.5773	
B-FA	0.6629	1	0.6629	13.99	0.0046	
C-CA	0.0377	1	0.0377	0.7960	0.3955	
D-w/c	0.1519	1	0.1519	3.21	0.1069	
E-B	0.0124	1	0.0124	0.2621	0.6210	
AB	0.3868	1	0.3868	8.16	0.0189	
AC	0.0971	1	0.0971	2.05	0.1861	
AD	0.0000	0				
AE	0.0208	1	0.0208	0.4389	0.5242	
BC	0.0000	0				
BD	0.0000	0				
BE	0.0187	1	0.0187	0.3948	0.5454	
CD	0.0000	0				
CE	0.0006	1	0.0006	0.0119	0.9156	
DE	0.0014	1	0.0014	0.0297	0.8670	
A^2^	0.0000	0				
B^2^	0.0000	0				
C^2^	0.0000	0				
D^2^	0.0000	0				
E^2^	0.0635	1	0.0635	1.34	0.2769	
Residual	0.4264	9	0.0474			
Lack of fit	0.1852	4	0.0463	0.9596	0.5020	Not significant
Pure error	0.2412	5	0.0482			
Cor total	2.36	21

**Table 15:** Fit statistics for the concrete splitting tensile strength.

SD	0.2177	R^2^	0.8191
Mean	2.53	Adjusted R^2^	0.5780
C.V. %	8.59	Predicted R^2^	NA
		Adeq Precision	6.4058

**Table 16:** Constraints for the concrete splitting tensile strength model.

Name	Goal	Lower Limit	Upper Limit	Lower Weight	Upper Weight	Importance
A:C	Is in range	350	456	1	1	3
B:FA	Is in range	555	861	1	1	3
C:CA	Is in range	900	1227.5	1	1	3
D:w/c	Is in range	0.4	0.5	1	1	3
E:B	Is in range	1000	1E+09	1	1	3
CS	Maximize	18.67	61.79	1	1	3
StdErr(CS)	None	1.17346	2.29627	1	1	3
STS	Maximize	1.8	3.2	1	1	3
StdErr(STS)	None	0.111229	0.217656	1	1	3

**Table 17:** Selected optimized STS solution from 100 solutions found.

Number	C	FA	CA	w/c	B	CS	StdErr(CS)	STS	StdErr(STS)	Desirability	
1	455.634	651.038	965.600	0.419	595,963,905.366	1804.376	1083.670	58.817	102.717	1.000	Selected

### Metaheuristic models and sensitivity analysis

**Figure 48:**
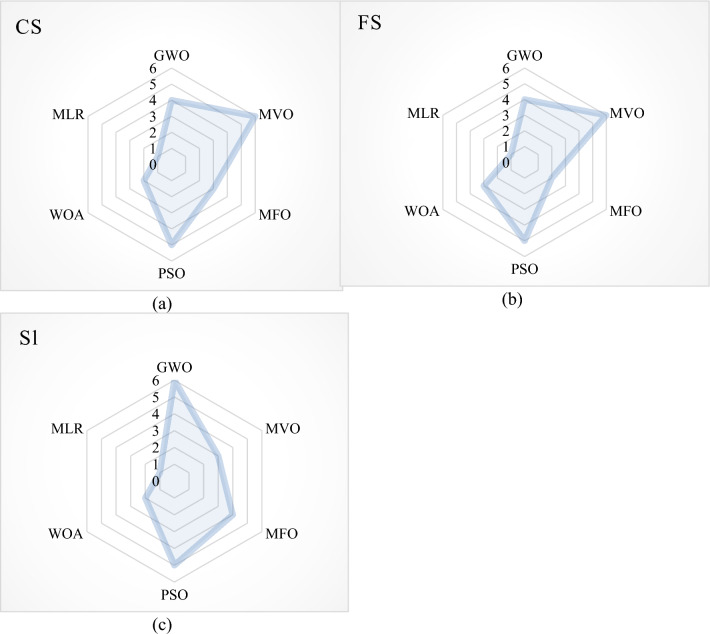
Comparing the results based on their rank.

The GWO, MVO, MFO, PSO, and WOA are the metaheuristic techniques applied in the optimization of the compressive strength, flexural strength, and slump of the SHC and these results are compared with the baseline regression; multilinear regression (MLR). Table 18 shows the detailed prediction performance evaluation of the metaheuristic models alongside the MLR. The performance indices used as shown in Table 18 are the VAF, MSE, RMSE, MAE, CE, and R^2^. In Table 19, the performance indices are ranked in a score analysis with respect to the outputs modeled in this extensive exercise. The score analysis was conducted to identify the most suitable model for each output of the dataset, both during the training and testing phases. To achieve this, a score of "n" was assigned to each model, where "n" represents the total number of proposed models (6 in this case). This score was used to determine the optimal value for each performance indicator. The models were subsequently ranked based on their individual performance indices, which are elaborated in Table 19 and Fig. 48. By aggregating the training and testing scores, an overall score was calculated for each model, providing a comprehensive evaluation of its performance. It can be shown that MVO is ranked 1^st^ in the prediction of the CS and FS of the SHC, while GWO is ranked 1^st^ in the prediction of the Sl of the SHC. Comparatively, it can be deduced from previous literature^49^, that the presented metaheuristic techniques present models, which have performed better than the novel ANN used previously.

**Table 18:** The models’ prediction performance evaluation.

Methods		Train						Test					
		VAF	MSE	RMSE	MAE	CE	R^2^	VAF	MSE	RMSE	MAE	CE	R^2^
CS	GWO	98.421	2.926	1.710	1.142	0.984	0.984	94.464	7.878	2.807	2.196	0.956	0.966
	MVO	99.770	0.427	0.653	0.447	0.998	0.998	95.498	7.016	2.649	2.040	0.952	0.958
	MFO	98.754	2.426	1.558	1.132	0.986	0.988	83.511	31.888	5.647	4.730	0.863	0.941
	PSO	98.790	2.245	1.498	1.100	0.988	0.988	91.438	12.246	3.499	2.764	0.936	0.954
	WOA	97.896	3.901	1.975	1.407	0.978	0.979	89.733	17.667	4.203	3.297	0.884	0.906
	MLR	82.438	32.548	5.705	3.964	0.787	0.824	44.906	91.672	9.575	7.446	0.386	0.540
FS	GWO	98.603	0.011	0.103	0.075	0.986	0.986	81.242	0.469	0.685	0.535	0.488	0.910
	MVO	98.890	0.008	0.092	0.066	0.989	0.989	85.312	0.340	0.583	0.426	0.738	0.885
	MFO	98.437	0.012	0.109	0.091	0.984	0.984	74.184	0.617	0.786	0.646	0.207	0.852
	PSO	98.905	0.008	0.091	0.075	0.989	0.989	78.545	0.568	0.754	0.523	0.240	0.937
	WOA	98.382	0.012	0.111	0.093	0.983	0.984	82.107	0.416	0.645	0.490	0.531	0.934
	MLR	47.646	0.495	0.704	0.428	-0.300	0.477	27.508	2.024	1.423	0.949	-14.439	0.474
Sl	GWO	99.751	0.179	0.423	0.330	0.997	0.998	92.987	8.770	2.961	1.809	0.952	0.989
	MVO	99.608	0.282	0.531	0.395	0.996	0.996	83.921	19.348	4.399	3.749	0.900	0.932
	MFO	98.427	1.215	1.102	0.880	0.981	0.986	96.591	6.471	2.544	1.953	0.950	0.969
	PSO	99.574	0.307	0.554	0.445	0.996	0.996	95.177	5.897	2.428	1.768	0.964	0.982
	WOA	96.112	2.816	1.678	1.263	0.955	0.963	94.340	6.867	2.621	2.337	0.947	0.948
	MLR	59.030	29.710	5.451	4.023	0.299	0.590	77.903	32.955	5.741	5.028	0.669	0.780

**Table 19:**
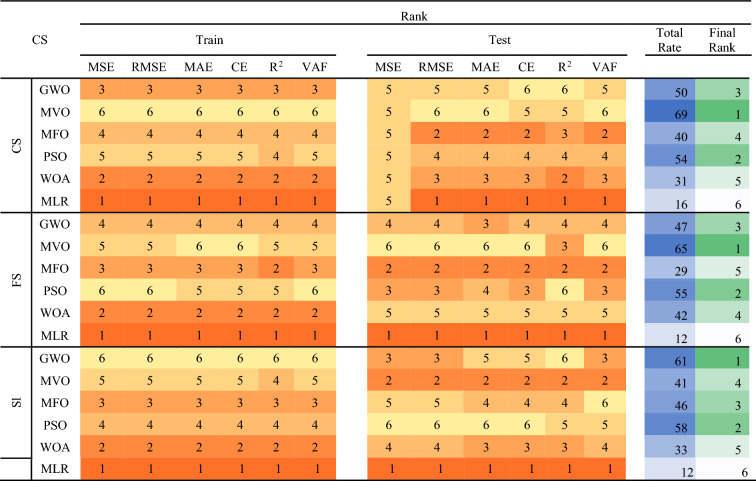
Score analysis for the developed all models.

The ranking color scale of the 6 techniques and the corresponding performance indices with rank 1 having the deepest of the color scales and rank 6 showing the lightest scale of the color ranks.

### Sensitivity analysis

The study employed sensitivity analysis to determine the relative influence of each parameter on the output within the model, utilizing the cosine domain method as proposed by Yang and Zang^50^. In order to implement this method, all data pairs were transformed into a shared X-space. To facilitate this technique, it was necessary to construct a data array X by incorporating all available data pairs according to the following procedure^51,52^:

11 $$ X = \left\{ {x_{1} ,x_{2} ,x_{3} , \ldots ,x_{i} , \ldots ,x_{n} } \right\} $$

Each of the elements, xi, in the data array X is a vector of lengths of m, that is:

12 $$ {\text{X}} = \left\{ {{\text{x}}_{1} 1,{\text{x}}_{2} 2,{\text{x}}_{3} 3, \ldots ,{\text{x}}_{{{\text{im}}}} } \right\} $$

The strength of the relation between the dataset, xi and xj, is presented as follows:

13 $$ {\text{r}}_{{{\text{ij}}}} = \frac{{\mathop \sum \nolimits_{{{\text{k}} = 1}}^{{\text{m}}} {\text{x}}_{{{\text{ik}}}} {\text{x}}_{{{\text{jk}}}} }}{{\sqrt {\mathop \sum \nolimits_{{{\text{k}} = 1}}^{{\text{m}}} {\text{x}}_{{{\text{ik}}}}^{2} \mathop \sum \nolimits_{{{\text{k}} = 1}}^{{\text{m}}} {\text{x}}_{{{\text{ik}}}}^{2} } }} $$

**Figure 49:**
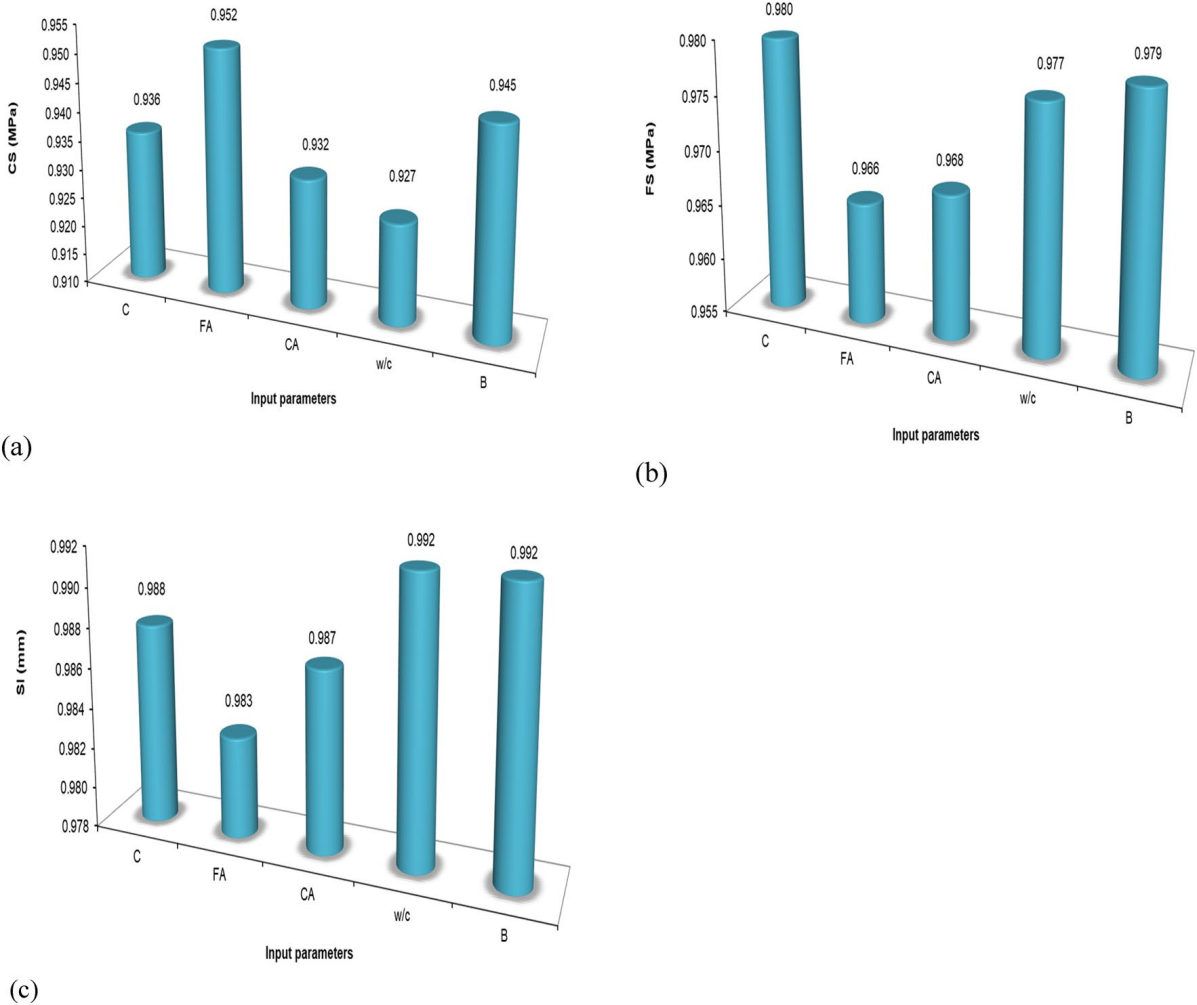
Sensitivity analysis to determine the impact of each data on the output.

The results as presented in Fig. 49 show that the FA is more sensitive than B, which followed closely to the behavior of the compressive strength (CS) of the SHC with an insignificant difference. For the flexural strength (FS), the cement shows to be more influential and again followed by the bacterial concentration (B). For the SHC slump, the most influential are the water/cement content again the bacterial concentration (B), which is at par with the w/c. These results for the CS, FS, and Sl model sensitivity analysis, the bacterial concentration (B) showed to be the second most influential parameter in the production design and behavior of the SHC and should be taken as one of the major decisive constituents needed to produce a reliable healing potential in concrete. Further these outcomes agree with the results of a previous research work^49^ which had utilized the same data entry capacity. It is understandable to note that w/c ratio is one of the most influential variables in the model as workability relies mostly on water content, water to cement or water to binder relationship.

Overall, the studies into the self-healing technology in concrete has been conducted previously by researchers, which had used capsules, microfibres, glass capsules, epoxy amines, etc. to trigger self-healing processes in concrete^29,30,42^. Subsequently, extensive reviews have been conducted^6,7^ on the self-healing processes in concrete enhancing durability index in concrete structures. Mentioned also was made of the utilization of both microencapsulated-based and bio-based^6,7^. Many research papers on the other hand mentioned the application bacterial-inspired self-healing processes in concrete^35,36,38,43–45^, but hardly apply intelligent predictions in the estimation of the concrete strengths. It was one closely related research work^49^, which applied the genetic programming (GP), evolutionary polynomial regression (EPR), and the artificial neural networks (ANN) to study the strengths of the bacterial-based self-healing concrete. The models compared well with the models of the present work but were outperformed due to the ability of the metaheuristic techniques used in this work to overcome overfitting. Finally, comparing the metaheuristic techniques and the symbolic regression method (RSM), the metaheuristics showed their superiority over the RSM, even though the RSM did not produce any predicted R-squared values rather it used adequate precision to judge its ability to predict the mechanical properties of the studied concrete. The RSM show adequate precisions, which are above the standard value (˃ 7.0). However, the RSM outperformed the LMR. The metaheuristic techniques are superior in their prediction capabilities due to the nature-based technology and ability to predict without being affected by overfitting.

## Conclusions

In this research work, the influence of bacteria concentration on the mechanical properties of self-healing concrete (SHC) for sustainable bio-concrete structures has been studied with the intelligent metaheuristic techniques, which include Gray Wolf Optimization (GWO), Multi-Verse Optimization (MVO), Moth-Flame Optimization (MFO), Particle Swarm Optimization (PSO) and Whale Optimization Algorithm (WOA) and the Response Surface Methodology (RSM). The concrete parameters considered in addition to the bacteria concentration in this model exercise include cement, fine aggregate, coarse aggregate, and water-cement ratio and these were utilized as input variables to predict the outputs; compressive strength, flexural strength and the slump. The performance of the models was also tested by using the coefficient of determination (R^2^), root mean squared errors (RMSE), mean absolute errors (MAE), mean squared errors (MSE), variance accounted for (VAF) and the coefficient of error (CE). The following can be concluded;

- The classified metaheuristic techniques outclassed the RSM due their ability to mimic human and animal genetics of mutation providing highly acceptable values of R^2^ and error metrics.
- The GWO outclassed the other methods in predicting the concrete slump (Sl) with R^2^ of 0.998 and 0.989 for the train and test, respectively.
- The PSO outclassed the rest in predicting the flexural strength (FS) with R^2^ of 0.989 and 0.937 for train and test, respectively.
- The MVO outclassed the others in predicting the compressive strength (CS) with R^2^ of 0.998 and 0.958 for train and test, respectively.
- The CS, FS, and Sl model sensitivity analysis shows that the bacterial concentration (B) showed to be the second most influential parameter in the production design and behavior of the SHC and should be taken as one of the major decisive constituents needed to produce a reliable healing potential in concrete.
- Overall, the GWO, PSO, and MVO having performed within acceptable limits are considered superior to the other models.
- The RSM did not generate R^2^ value rather the adequate precision in computation has been used to judge its ability to predict the mechanical properties of the bacterial-inspired self-healing concrete, which is considered adequate above 7.0. It further proposed closed-form polynomial relationships between the regressors and the outputs, which can be applied manually in the prediction of the SHC mechanical properties.

## Data Availability

The data supporting the outcome of this research work is available on reasonable request from the corresponding author.
